# Preparing fourth year medical students to care for patients with opioid use disorder: how this training affects their intention to seek addiction care opportunities during residency

**DOI:** 10.1080/10872981.2022.2141602

**Published:** 2022-11-04

**Authors:** Katharine F. Marshall, Patricia A. Carney, Kathryn J. Bonuck, Patricio Riquelme, Jonathan Robbins

**Affiliations:** Division of General Internal Medicine & Geriatrics, Section of Addiction Medicine, Wise Fellow in General Internal Medicine, Portland, OR, USA

**Keywords:** Undergraduate medical education, transition to residency, medication for opioid use disorder, addiction medicine

## Abstract

**Background & Objectives:**

In 2021, the USA recorded 100,000 annual deaths from drug overdose, representing the most frequent cause of death in adults under age 55. The integration of care for substance use disorders (SUDs) into undergraduate medical education is not well established. It is unclear whether a short course on management of opioid use disorder (OUD) offered to fourth year medical students could increase graduating students’ knowledge and preparedness to treat these disorders.

**Methods:**

We designed a 2-hour interactive case-based session on patient care for OUD and delivered it virtually as part of a Transition to Residency course. A retrospective pre-/post-test assessment instrument determined the impact of this session on students’ perceived knowledge, confidence, and intention to seek further educational opportunities for OUD.

**Results:**

Of 144 participants, 58 students (40.3%) completed the retrospective pre-/post- survey. There were statistically significant improvements in perceived knowledge and attitudes on the 12-item survey. The largest gains in perceived knowledge on a 5-point scale occurred in the categories regarding buprenorphine induction (*pre 2.9; post 4.22; p* < 0.001), managing inpatient opioid withdrawal (*pre 2.84; post 4.27; p* < 0.001), and the role of methadone in treating withdrawal (*pre 3.16; post 4.29; p* < 0.001). All (n = 58) survey respondents would recommend the training to a colleague and felt that the session would benefit their professional practice. Over 90% (93.1%) of respondents planned on seeking additional SUD learning opportunities during residency.

**Conclusions:**

A 2-hour interactive case-based teaching session delivered to medical students improved perceived knowledge, attitudes, and future interest in obtaining education around OUD. As the opioid epidemic shows no sign of abating, we would advocate for the inclusion SUD education as part of Transition to Residency courses.

## Introduction

In April 2021, for the first time, the USA recorded 100,000 annual deaths from drug overdose. Accidental overdose now represents the most frequent cause of death in adults under age 55, with more than 70% of these deaths due to opioid use and, in particular, synthetic opioids such as fentanyl.^1–3^ Despite recent decreases in opioid prescribing, Oregon currently ranks above the national average in per capita opioid prescriptions and reports the highest rate of prescription pain reliever misuse among all 50 states.^4,5^ Additionally, while nearly 1 in 5 adult Oregonians reported an active substance use disorder (SUD) within the last year, Oregon ranks highest in the nation for patients needing, but not receiving, specialty addictions care.^5,6^ As a result, physicians across all specialties can expect to frequently encounter patients with active opioid use disorder (OUD) and its complications.

Medication for opioid use disorder (mOUD) is recommended for all patients with moderate-to-severe OUD, with U.S. Food and Drug Administration (FDA)-approved options consisting of methadone, buprenorphine, or injectable naltrexone.^7^ Under federal law, methadone for OUD can only be dispensed through separately licensed Opioid Treatment Programs (OTPs) or in hospital settings.^8^ The Drug Addiction Treatment Act of 2000 (DATA-2000) opened the door for an alternative therapy, buprenorphine, in primary care or other office-based settings provided prescribing practitioners apply for a Drug Enforcement Agency (DEA) waiver (‘X-waiver’). Prior to 2021, physicians were required to obtain 8 hours of dedicated education before prescribing.^9^ The required buprenorphine education was designed for providers already in practice and licensed under State law, however few eligible physicians have obtained the X-waiver and most who have obtained it treat just a handful of patients.^10–12^ The COVID-19 pandemic has since exacerbated the opioid crisis, and in April 2021 the federal government waived the educational mandate for practicing physicians, instead requiring only that a notice of intent be submitted to the DEA for a provider to treat up to 30 concurrent patients with buprenorphine.^13,14^ As of this writing, the Mainstreaming Addiction Treatment Act of 2021 is under review by the USA Congress and would eliminate the need for an X-waiver for practitioners to prescribe buprenorphine for OUD, provided they are qualified to prescribe other controlled substances.^15^

Fourth-year medical students matriculating into residency in this climate face a unique challenge because the integration of care for SUD into undergraduate medical education (UME) and graduate medical education (GME) is not well established.^16,17^ One statewide survey of fourth-year medical students found that only 15.9% ‘strongly agreed’ that they had been adequately trained in care of SUD before entering residency, and fewer than 15% ‘strongly agreed’ that they felt prepared to screen for OUD or counsel a patient on options for mOUD.^18^ And while 75% of residency program directors in psychiatry, family medicine and internal medicine reported their residents ‘frequently’ care for patient with OUD, fewer than 25% reported that their program actively encourages or requires training in the use of buprenorphine for OUD.^19^

In recent years ‘Transition to Residency’ courses have become an increasingly popular offering for fourth year medical students, with over 70% of medical schools offering this to all graduating students in 2020–2021 and 45% offering specialty-specific courses.^20,21^ The courses typically review or introduce high-yield material deemed critical for students before matriculation, frequently covering topics such as working with electronic health records, patient safety and error disclosure, communication skills and overviews of specific disease management.^20^ We sought to determine whether a 2-h session on the management of OUD offered during transition to residency could achieve improvements in student knowledge and confidence around the treatment of this commonly encountered disease.

In early 2022, we designed a 2-h interactive case-based session on patient care for OUD and delivered it virtually to fourth-year medical students as part of a Transition to Residency course. We designed a retrospective pre-/post- assessment instrument to determine the impact of this session on students’ perceived knowledge and intention to attain additional educational experience when they enter residency.

## Methods

### Setting and Instructional Development of the Session

Oregon Health & Science University (OHSU) is the only allopathic medical school in the state of Oregon. Approximately 150 medical students matriculate into its time-varying competency-based curriculum each year. There are three ‘transitions’ courses in the curriculum: Transition to Medical School, Transition to Core Clinical Training, and Transition to Residency. By 2021, the UME didactic curriculum included 2 hours of mandatory material on substance use disorders (1 hour on neurobiology and 1 hour on diagnosis and management), with the option for interested students to also enroll in a preclinical elective and/or a selective intersession. We presented a curricular plan that would add an additional 2 hours of dedicated SUD instruction to the required Transition to Residency course for fourth-year students, which was approved by OHSU’s Curriculum Committee in May 2021.

Content of the session included instruction in the diagnosis of SUD according to criteria from the Diagnostic and Statistical Manual of Mental Disorders, Fifth Edition (DSM-5), a review of the FDA-approved medications for OUD and their mechanisms of action, management of acute opioid withdrawal in the emergency department and inpatient settings, review of buprenorphine initiation strategies, an approach to acute pain management in a patient with OUD, an approach to high-risk behaviors such as in-hospital use of substances, and a review of harm reduction strategies for patients who use substances. It was delivered virtually by two faculty members using didactic and case-based learning and incorporating an online audience response tool (Mentimeter; mentimeter.com), affording students an opportunity to ‘choose their own adventure’ by selecting the next management step in realistic clinical cases (see **Appendix A** for educational materials).

### Assessment Instrument and Data Collection

A 12-item assessment tool was developed based on the learning objectives of the training. It assessed students’ perceived knowledge about recognizing, screening, and treating patients with SUD using a retrospective pre-/post- survey design and a 5-point Likert scale (1 = Strongly disagree; 2 = Disagree; 3 = Neither agree nor disagree; 4 = Agree; 5 = Strongly agree). The same 5-point Likert scale was also used to assess 10 items related to students’ satisfaction with the training, their confidence levels, and intention to seek additional learning opportunities caring for patients with SUD during residency. Two open-ended questions asked students what they liked best about the session and what would make it better. The survey was built in Qualtrics and administered via the Internet to students using an anonymous web-based link at the end of the session. A reminder email was sent once to non-responders. OHSU’s Institutional Review Board (IRB) approved all activities (IRB# 10,873).

### Data Analyses

Analyses were performed using IBM SPSS Version 28. For the post-program student satisfaction survey, we collapsed ‘strongly disagree’ and ‘disagree’ categories into a single category and collapsed ‘agree and strongly agree’ into one agreement category and reported frequencies. Means, 95% confidence intervals (CI), skewness, kurtosis, z scores and r values were calculated for the self-reported knowledge differences pre and post oud training. We removed four responses from students who did not complete both sections of the retrospective pre-/post assessment. We used the Wilcoxon Signed Rank test to compare differences in perceived knowledge among students for the retrospective pre-/post- survey, since the data is non-normally distributed. Statistical tests were 2-tailed with alpha set at 0.05 to determine statistical significance. Openended comments were analyzed using classical content analysis.^22^

## Results

The assessment instrument was sent to 144 fourth-year medical students. Fifty-eight students returned surveys with the program evaluation section completed (40.3%) and 51 completed the retrospective pre-/post- questions (35.4%). Mean pre-training knowledge scores ranged from *2.84 (95% CI = 2.61–3.08)* for understanding how to manage opioid withdrawal among patients with OUD in the emergency department and inpatient settings to *3.78* for both knowing how to recognize SUDs and understanding the respectful communication strategies needed to work with patients with SUDs All 12 knowledge items improved statistically between pre- and post-training (p < 0.001) with the highest scores post training for understanding the respectful communication strategies needed to work with patients with SUDs (*mean = 4.51; 95%CI = 4.37–4.65; p < 0.001*). The effect size was *r* = −.60 for understanding how to manage opioid withdrawal among patients with substance use disorder in the ED and inpatient settings (pre *mean = 2.84*, post *mean* = 4.27; *z* = −6.06, *p* < 0.001). Table 1

**Table 1.** Students self-reported knowledge differences pre and post the opioid use disorder training (n = 51)

Question:	Pre-Training				Post Training				*Z*	*r*	*p-*
	Mean	*95%* *CI*	Skewness (SE)	Kurtosis (SE)	Mean	*95%* *CI*	Skewness (SE)	Kurtosis (SE)	Score	Value	Value*
I understand how to **recognize** substance use disorders	3.78	3.58– 3.99	−1.57 (.33)	4.14 (.66)	4.35	4.20– 4.51	−.10 (.33)	−.74 (.66)	−4.37	.43	<.001
I understand how to **screen** patients for substance use disorders	3.49	3.25– 3.73	−.56 (.33)	.42 (.66)	4.35	4.21– 4.50	.19 (.33)	−1.02 (.66)	−5.39	.53	<.001
I understand how to **diagnose** patients with substance use disorders	3.59	3.36– 3.82	−.84 (.33)	1.05 (.66)	4.31	4.16– 4.47	.04 (.33)	−.61 (.66)	−4.90	.49	<.001
I understand how to evaluate patients for opioid withdrawal	3.65	3.40– 3.90	−.64 (.33)	.57 (.66)	4.43	4.29– 4.57	.29 (.33)	−2.00 (.66)	−5.12	.51	<.001
I understand how to use the Clinical Opiate Withdrawal Scale (COWS) with patients	3.33	3.03– 3.63	−.21 (.33)	−.64 (.66)	4.39	4.21– 4.57	−1.04 (.33)	2.40 (.66)	−5.49	.54	<.001
I understand the pharmacology of medications for opioid use disorder	3.63	3.40– 3.86	−.99 (.33)	1.34 (.66)	4.35	4.21– 4.50	.19 (.33)	−1.02 (.66)	−5.11	.51	<.001
I understand how to manage opioid withdrawal among patients with substance use disorder in emergency inpatient settings	2.84	2.61– 3.08	.09 (.33)	.01 (.66)	4.27	4.11– 4.43	−.04 (.33)	−.42 (.66)	−6.06	.60	<.001
I understand buprenorphine induction	2.90	2.61– 3.19	−.26 (.33)	−.80 (.66)	4.22	4.06– 4.37	.14 (.33)	−.05 (.66)	−5.96	.59	<.001
I understand the use of methadone for opioid withdrawal	3.16	2.92– 3.40	−.31 (.33)	−.58 (.66)	4.29	4.14– 4.45	.11 (.33)	−.52 (.66)	−5.52	.55	<.001
I understand how to manage pain in patients with substance use disorders	3.08	2.82– 3.34	−.01 (.33)	−.87 (.66)	4.27	4.11– 4.44	−.19 (.33)	−.50 (.66)	−5.92	.59	<.001
I understand the respectful communication strategies needed to work with patients with substance use disorders	3.78	3.55– 4.02	−.87 (.33)	1.63 (.66)	4.51	4.37– 4.65	−.04 (.33)	−2.08 (.66)	−5.24	.52	<.001
I understand how to reduce harms associated with substance use disorders in emergency and inpatient settings and upon discharge	3.49	3.26– 3.72	−.51 (.33)	.62 (.66)	4.41	4.27– 4.55	.37 (.33)	1.94 (.66)	−5.52	.55	<.001

*Wilcoxon Rank Sign Test

CI: Confidence Interval for Mean, Lower Bound and Upper Bound

Removed seven who did not fully complete pre- and post-test surveys

†Scale: 1 = Strongly disagree; 2 = Disagree; 3 = Neither agree nor disagree; 4 = Agree; 5 = Strongly agree

The post-program satisfaction survey indicated that 100% of students agreed or strongly agreed that they would recommend this training to a colleague and that it would benefit their professional development or practice Table 2. Over 90% (93.1%) agreed or strongly agreed that they feel confident in applying what they learned in their work with patients with SUD, and this same percentage agreed or strongly agreed that they plan on seeking more learning opportunities in the care of patients with SUDs when they enter residency training. Students felt that they had sufficient interaction with the instructors (100%) and that there was enough time in the session to have all of their questions answered (98%).

**Table 2.** Post program student satisfaction assessment (n = 58).

	Strongly Disagree/Disagree	Neutral	Agree/Strongly Agree
	n (%)	n (%)	n (%)
The case studies really helped me understand how to apply what I have learned when treating patients with substance use disorder	0 (0.00)	3 (5.2)	55 (94.8)
I feel knowledgeable about how best to work with patients with substance use disorder	0 (0.00)	5 (8.6)	53 (91.4)
I received tools that will help me succeed in my work with patients who have substance use disorder	0 (0.00)	1 (1.7)	57 (98.3)
I feel confident in applying what I learned today in my work with patients with substance use disorder	0 (0.00)	4 (6.9)	54 (93.1)
I would recommend undertaking this training to a colleague	0 (0.00)	0 (0.00)	58 (100)
Those who facilitated the sessions were highly effective	0 (0.00)	1 (1.7)	57 (98.3)
The presentations provided the right balance between providing information and interacting with participants	0 (0.00)	1 (1.7)	57 (98.3)
There was enough time to have all my questions answered	0 (0.00)	0 (0.00)	58 (100)
I expect this event to benefit my professional development and/or practice	0 (0.00)	0 (0.00)	58 (100)
I plan on seeking more learning opportunities in the care of patients with substance use disorders when I enter residency training	0 (0.00)	4 (6.9)	54 (93.1)

Qualitative analyses identified 2 emergent themes Table 3 for favorable aspects of the training session, which included characteristics of the facilitators and the case-based learning strategies. Highlights include the high level of engagement undertaken by facilitators who were passionate about the topic, while conveying the importance of being non-judgmental advocates for patients. The case-based learning strategies were noted to allow learners to interact with complex clinical cases based on real patients and used questions in ways that reinforced the challenges involved in caring for patients with OUD. Participants indicated that including a quick reference handout to provide readily accessible information for learners could improve the session, and they also indicated they wanted more information on medication management. Lastly, the session was held at the end of the day and some participants indicated they prefer it was held earlier in the day.

**Table 3.** Aspects of the session students liked best and areas for improvement.

Emergent Theme	Description/Frequency
**Favorable Aspects of the Session**	
Characteristics of Facilitators	Educators who facilitated the session were very engaging, passionate about the topic, and conveyed the importance of being non-judgmental advocates for patients. They provided very helpful answers to learner questions. (n = 16 mentions)
Case-based Learning	This technique allowed learners to interact around complex clinical cases built upon real patients and used questions in ways that reinforced the challenges involved in caring for patients with Opioid Use Disorder (OUD). (n = 28 mentions)
**Areas to Improve**	
Handout	Including a one page handout would help to provide readily accessible information for learners. (n = 3 mentions)
Medication Management	Learners indicated that additional information on managing medications would be helpful, such as dosing, morphine eqs calculation, management of long-term buprenorphine, pharmacology differences between drugs, and medication management for alcohol use disorder (n = 6 mentions)
Timing of Session	Placement of this sessions was felt to be too late in the day with screen fatigue being a concern. (n = 2 mentions)

## Discussion

Our study is among the first to show that a 2-hour training session on OUD increases both self-reported knowledge and confidence to treat OUD within a multispecialty Transition to Residency course. Universal improvement in reported knowledge across all 20 items, especially when combined with the high number of students reporting satisfaction with the effectiveness of teaching, suggests that even a brief course can improve learning.

Researchers at Wake Forest School of Medicine reported on an 11-hour course on recognition and treatment of OUD, including completion of required education to obtain an X-waiver, in its Transition to Residency curriculum. They included 8 hours of asynchronous online modules developed by the Providers and Clinical Support System (PCSS) and 3 in-person seminars (3 hours). They saw knowledge gains, high course satisfaction, and that students reported timing of the course was optimal.^23^ In their study, they included pre- and post- knowledge asessments and a survey 3 months into intern year; in the future our group hopes to implement these methods.

Many of the students at OHSU had already completed their x-waiver prior to the Transition to Residency Course, and the direct, interactive instructional time was similar between the Wake Forest and OHSU curriculum (3 hours vs 2 hours). Given competing curricular demands, particularly during a high yield transition to residency course, UME curriculum directors may wish to implement a similar but shorter course to reinforce mOUD skills prior to graduation. It may also be harder to obtain curricular time with the recent elimination of the requirement for 8 hours of education to obtain an X-waiver.

Many graduating students are entering specialties that offer little additional training in addiction medicine as part of GME curriculum, yet the high prevalence of disease indicates that most physicians will encounter it in practice. Another key finding of this study is the high number of students indicating their intention to seek further education in the care of OUD and SUD, which is particularly heartening for those entering non-generalist specialties who might not otherwise provide it.

In 2020–2021, 96.8% of responding medical schools reported that students received instruction in the treatment of substance use disorders; however, only 40% of schools reported any required didactics on the topic during clinical years.^24^ Transition to Residency courses offer an opportunity to emphasize certain topics as part of a greater transition strategy between UME and GME programs.^25^ This type of course has been associated with improvement in knowledge and an increased overall perception of readiness for residency.^26,27^ Dedicated curricular time for opioid stewardship and buprenorphine waiver training during this transition has been shown to improve knowledge and is associated with high satisfaction among students.^23^ Other studies have shown that brief sessions can lead to improved learning and learner satisfaction in medical training.^28,29^ Our research similarly suggests that the Transition to Residency course may be an opportune setting for a brief, high-yield training covering many of the same topics but requiring less curricular time than formal X-waiver training, depending on the needs of the specific program.

One strength of this study is the post- + retrospective pre-test design, which avoids the effects of pre-test sensitization and response-shift bias as well as other validity concerns with traditional pre-/post- survey designs. Other strengths are that the course was required for all graduating medical students and is potentially adaptable to either in-person or virtual instruction as needed.

There were several limitations to this study. First, the lower-than-expected response rate may have led to response bias, as students more interested in this topic may have been more likely to complete the survey. The retrospective, self-reported pre-/post- survey format cannot capture measurable change in actual behavior or objective knowledge. However, applying our study to Kirkpatrick’s modified training/evaluation model results in our addressing Level 1 (Participant Reaction) and Level 2a (Changes in Attitudes).^30^ It was not possible for us to assess Level 2b (Changes in Knowledge or Skills), Level 3 (Behavioral Change) or Levels 4a (Change in Healthcare Delivery or 4 b (Change in Patient Outcomes) because these were beyond the scope of this study. Nevertheless, we know from the Theory of Planned Behavior Change, which been successfully used to predict and explain a wide range of health behaviors and intentions including smoking, drinking, health services utilization, breastfeeding, and substance use, that behavioral achievement depends on both motivation (intention) and ability (behavioral control).^31^ We did assess both intention and confidence associated with our brief intervention and we believe this is valuable to report, given the population relevance and clinical importance of this topic. To address this issue in future research, we will add outcome assessments across more than one student class to determine the efficacy of the 2-h session on students’ knowledge retention of OUD and SUD. In addition, though students served as their own comparison group in the retrospective pre-post design, the study lacked randomization to a control versus intervention group. However, this topic is so important that restricting training in a control group is not desired. The use of an outcome measure tailored specifically to this training limits comparisons of our results to similar studies. Finally, given that the course was taught by specialist instructors at a single institution, the results are not generalizable to other institutions.

Future directions for research could include administration of a follow-up survey to assess durability of the reported knowledge gains and opportunities for further training for patients SUD. Studies using more rigorous measures such as simulation, assessments of prescribing patterns, reporting on the number of X-waivers obtained by participants, or reporting on actual care provided to this population would be beneficial.

 In conclusion, a 2-h interactive case-based teaching session delivered during a Transition to Residency course resulted in improvements in perceived knowledge, attitudes, and future interest in obtaining education around OUD. Students had low pre-test scores in perceived knowledge about buprenorphine initiation and managing opioid withdrawal in inpatient settings, indicating a critical need for targeted education prior to starting residency. As the opioid epidemic shows no sign of abating, we would advocate for the inclusion of OUD and SUD education as part of UME Transition to Residency courses.

## Supplementary Material

Supplemental Material
