# Supplementary material for: Preparing fourth year medical students to care for patients with opioid use disorder: how this training affects their intention to seek addiction care opportunities during residency
Source: Med Educ Online. 2022 Nov 4;28(1):2141602. doi: 10.1080/10872981.2022.2141602 (PMC9645269; doi:10.1080/10872981.2022.2141602)
Supplement: Supplemental Material [file ZMEO_A_2141602_SM1645.pptx]

## Slide 1
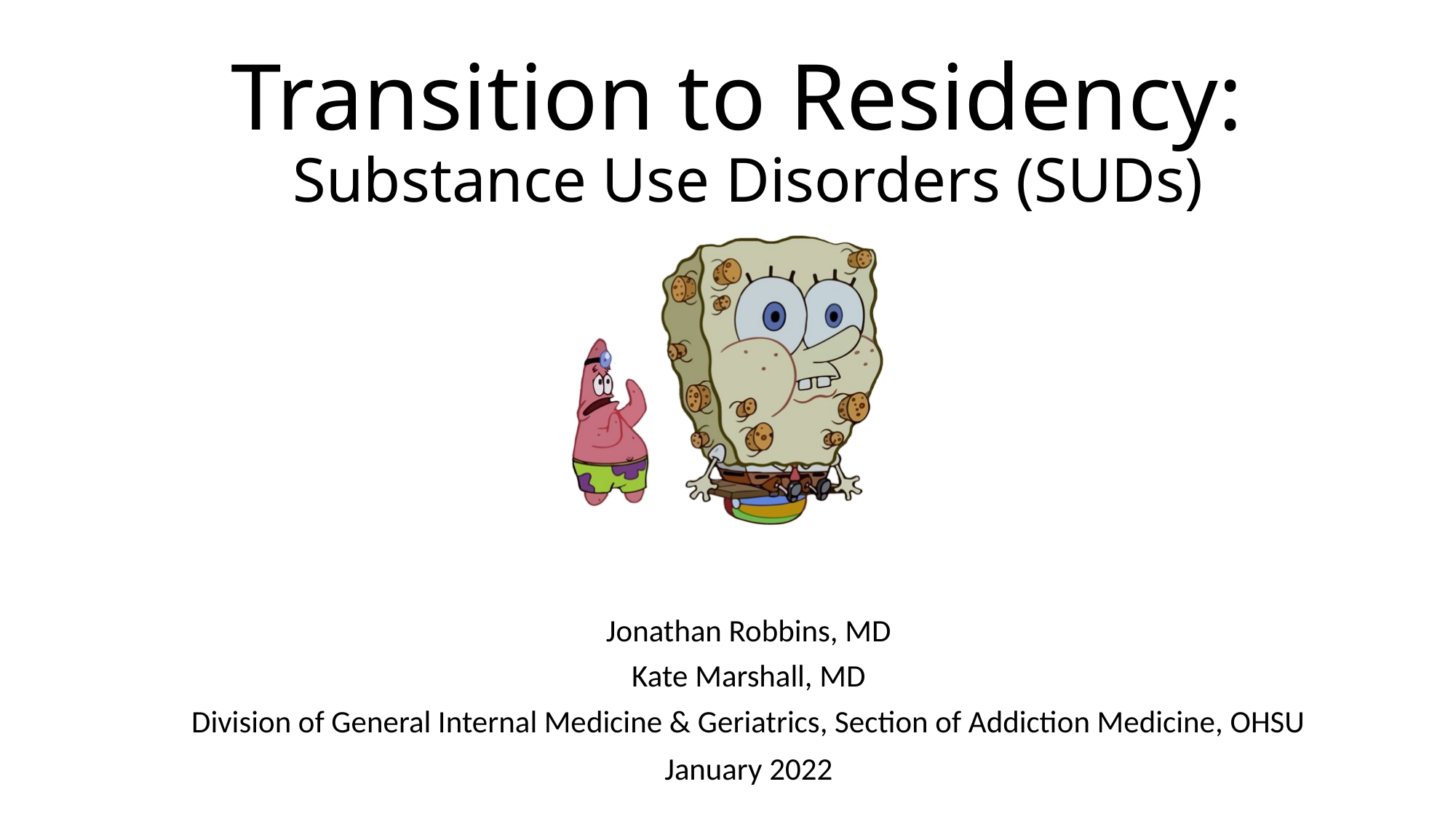

# Transition to Residency: Substance Use Disorders (SUDs)
Jonathan Robbins, MD
Kate Marshall, MD
Division of General Internal Medicine & Geriatrics, Section of Addiction Medicine, OHSU
January 2022

## Slide 2
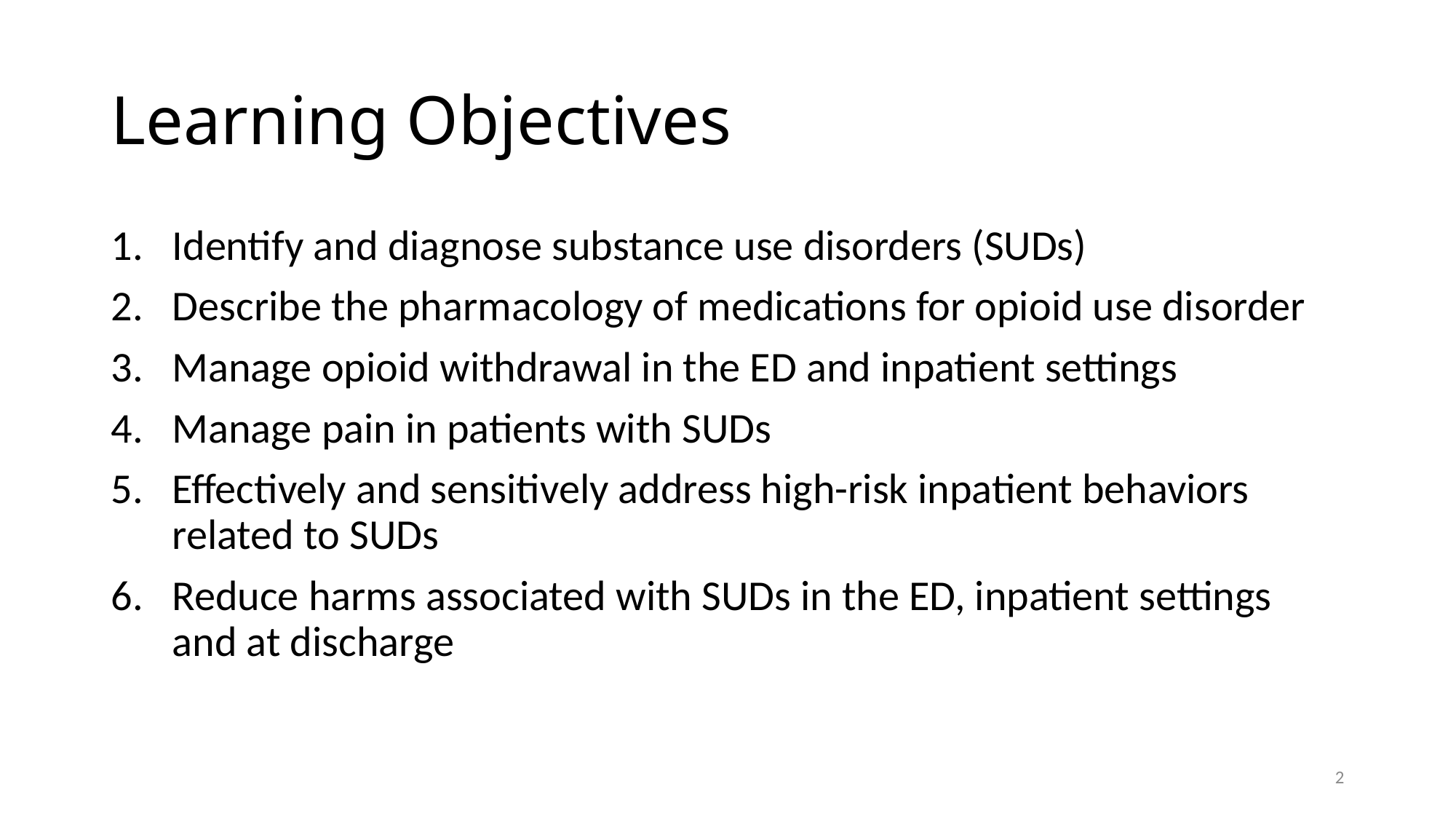

# Learning Objectives
Identify and diagnose substance use disorders (SUDs)
Describe the pharmacology of medications for opioid use disorder
Manage opioid withdrawal in the ED and inpatient settings
Manage pain in patients with SUDs
Effectively and sensitively address high-risk inpatient behaviors related to SUDs
Reduce harms associated with SUDs in the ED, inpatient settings and at discharge
2

## Slide 3
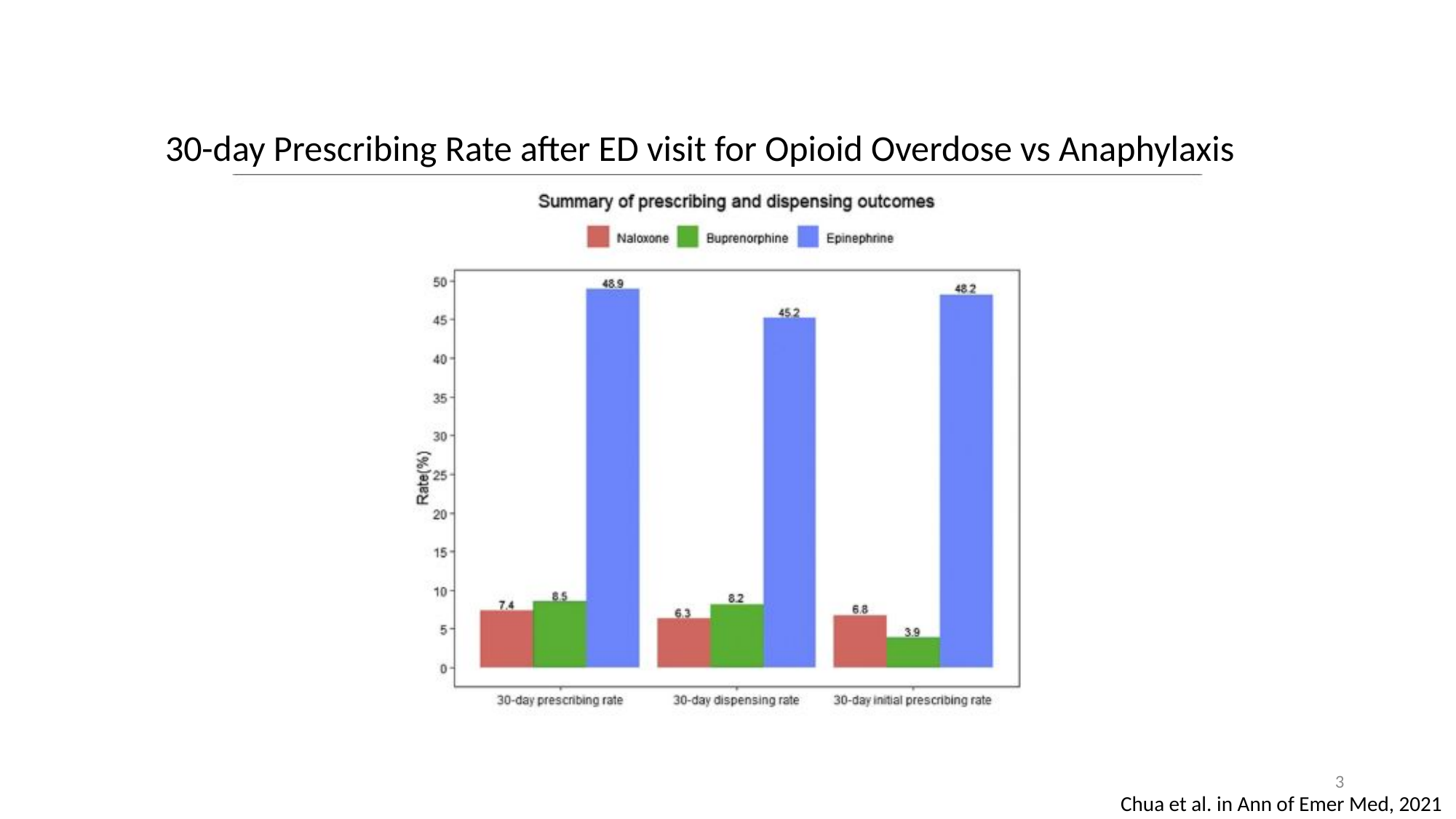

30-day Prescribing Rate after ED visit for Opioid Overdose vs Anaphylaxis
3
Chua et al. in Ann of Emer Med, 2021

## Slide 4
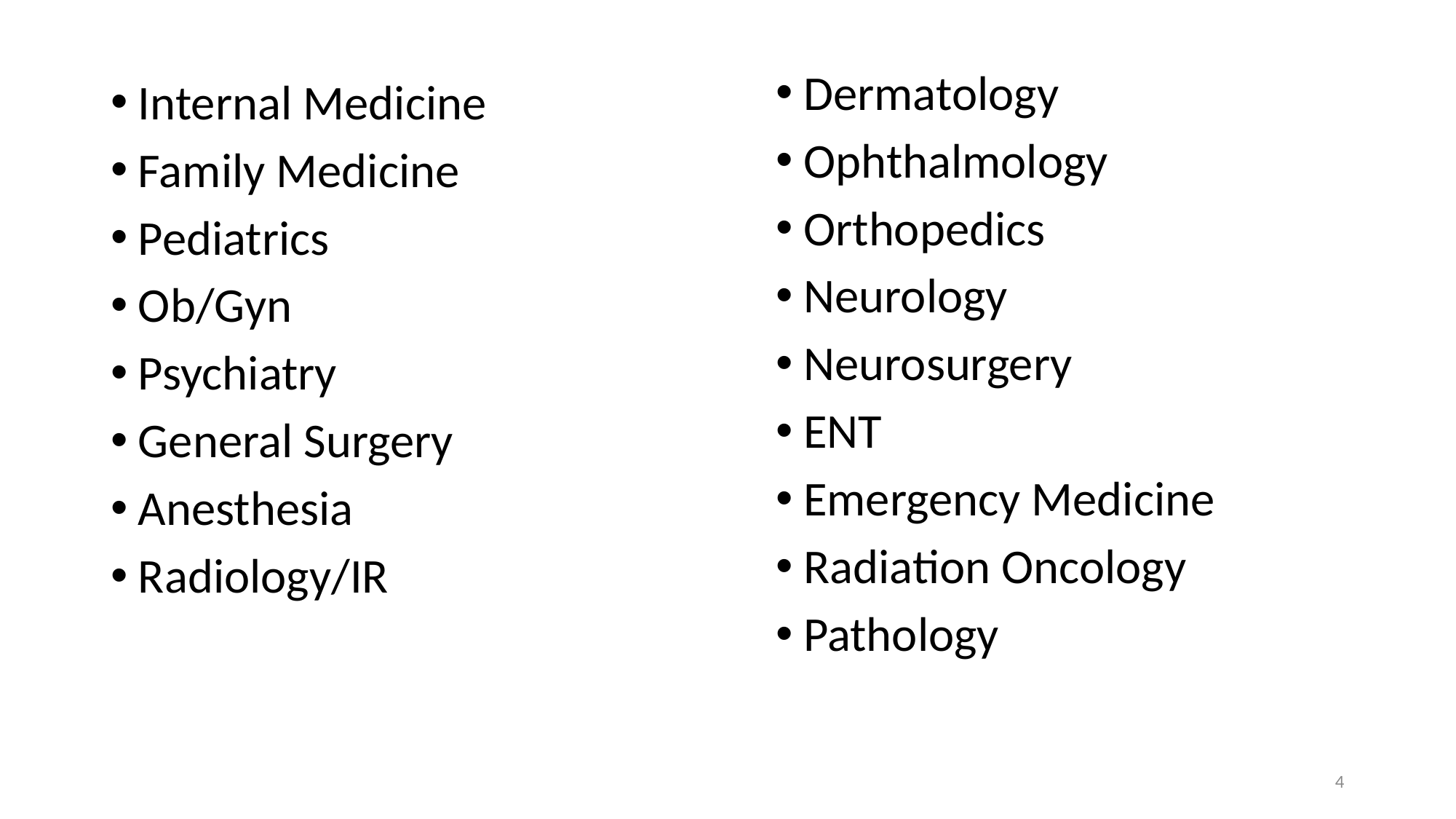

Dermatology
Ophthalmology
Orthopedics
Neurology
Neurosurgery
ENT
Emergency Medicine
Radiation Oncology
Pathology
Internal Medicine
Family Medicine
Pediatrics
Ob/Gyn
Psychiatry
General Surgery
Anesthesia
Radiology/IR
4

## Slide 5
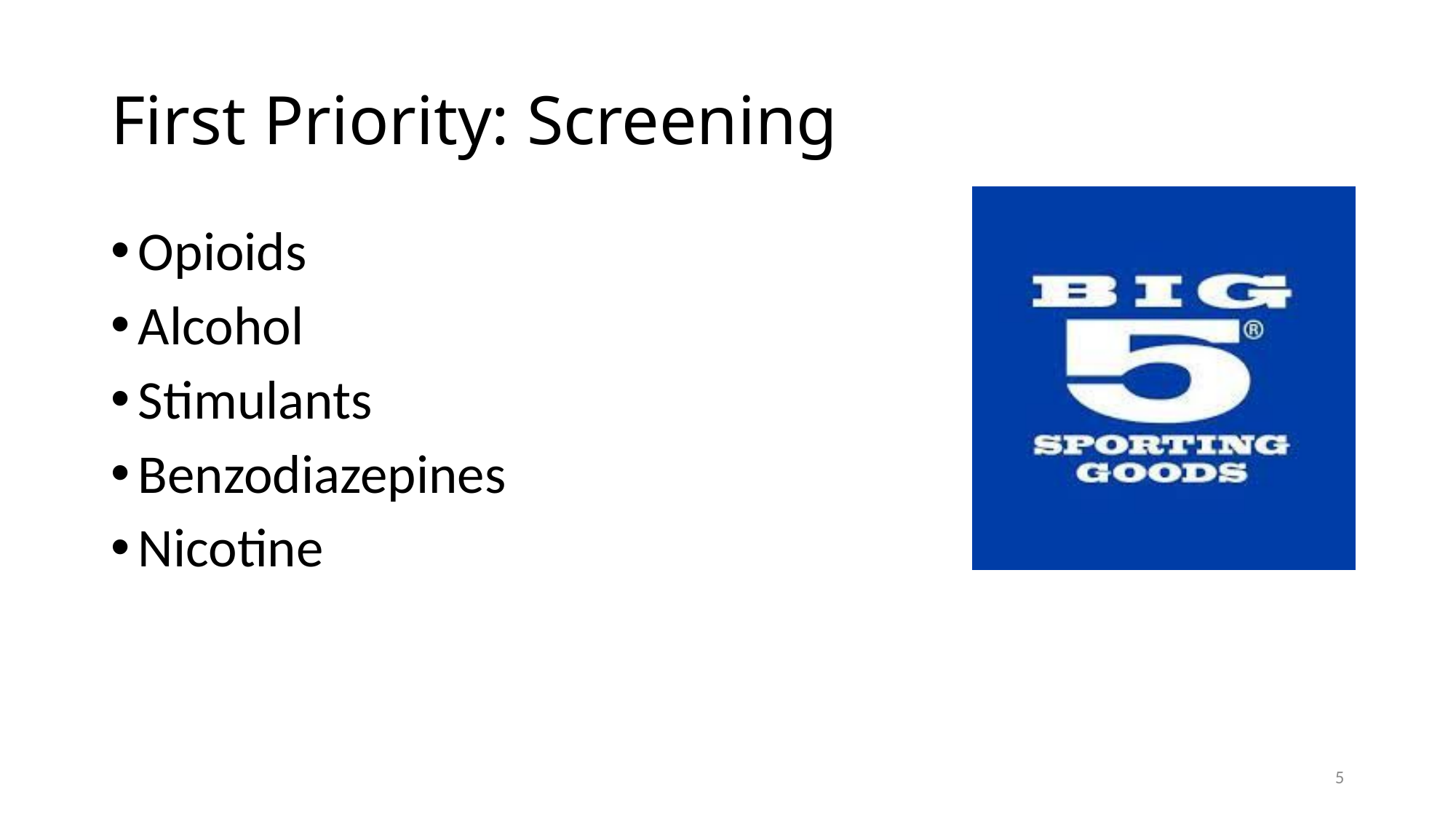

# First Priority: Screening
Opioids
Alcohol
Stimulants
Benzodiazepines
Nicotine
5

## Slide 6
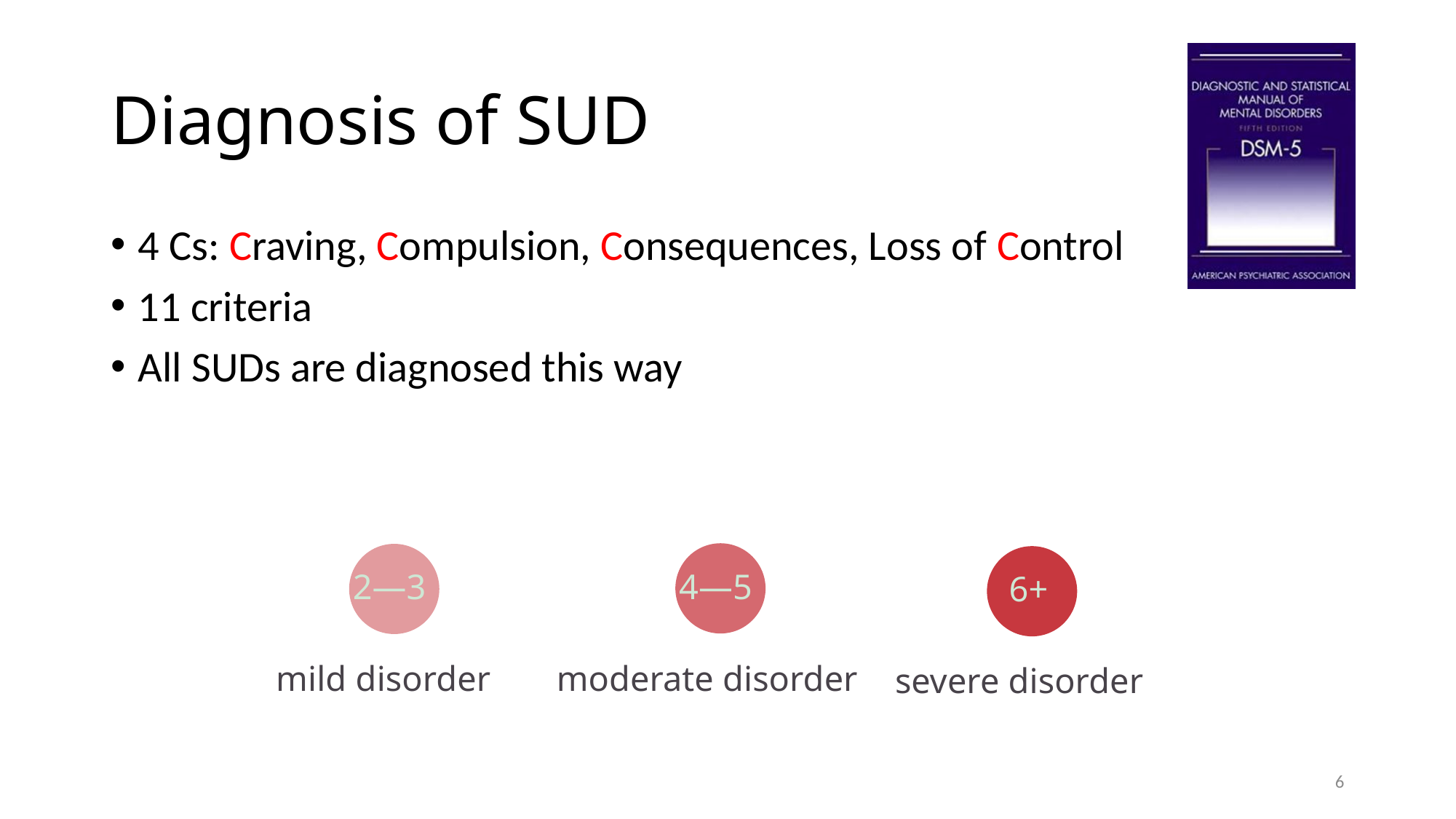

# Diagnosis of SUD
4 Cs: Craving, Compulsion, Consequences, Loss of Control
11 criteria
All SUDs are diagnosed this way
4—5
moderate disorder
2—3
mild disorder
6+
severe disorder
6

## Slide 7
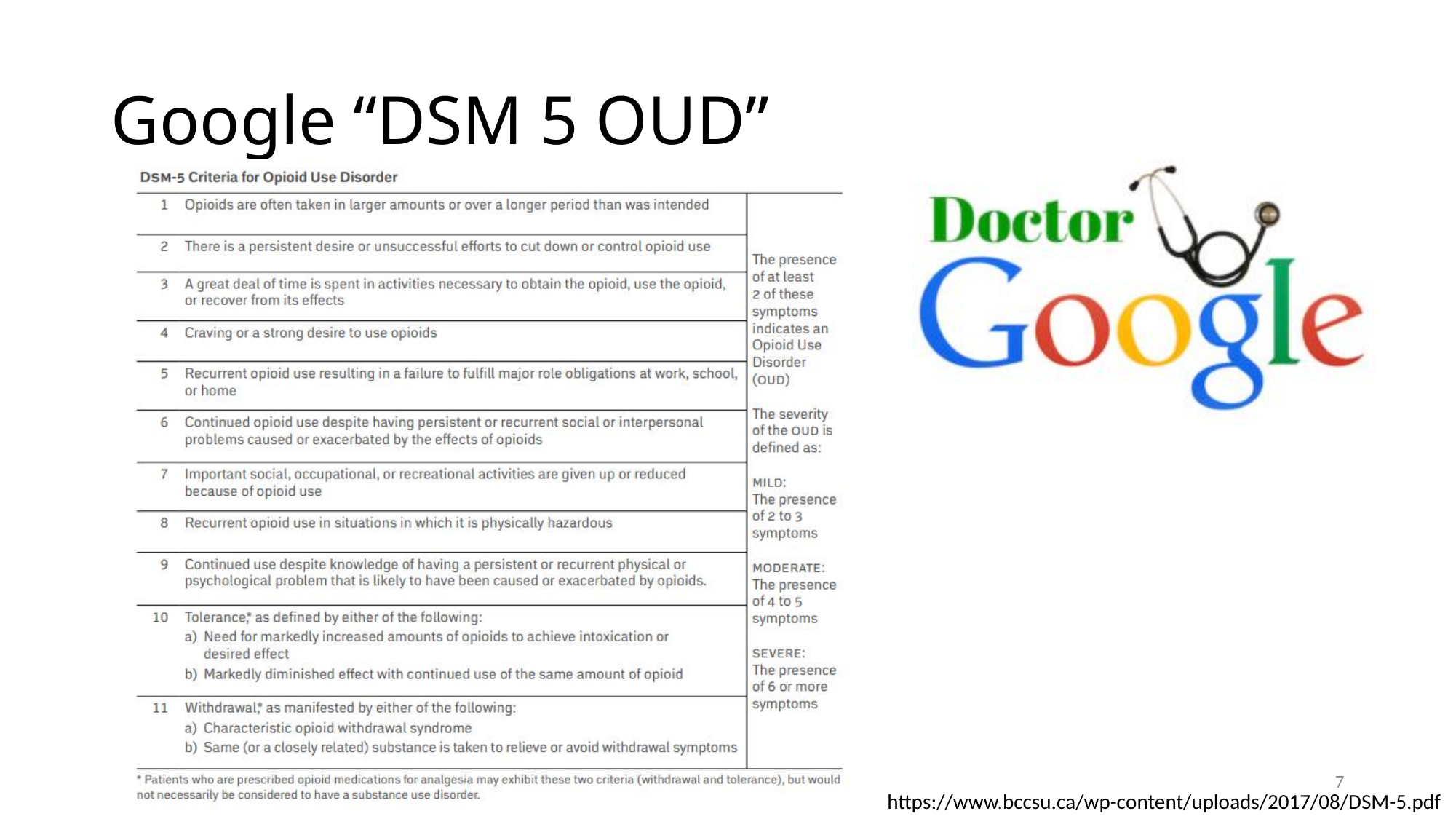

# Google “DSM 5 OUD”
7
https://www.bccsu.ca/wp-content/uploads/2017/08/DSM-5.pdf

## Slide 8
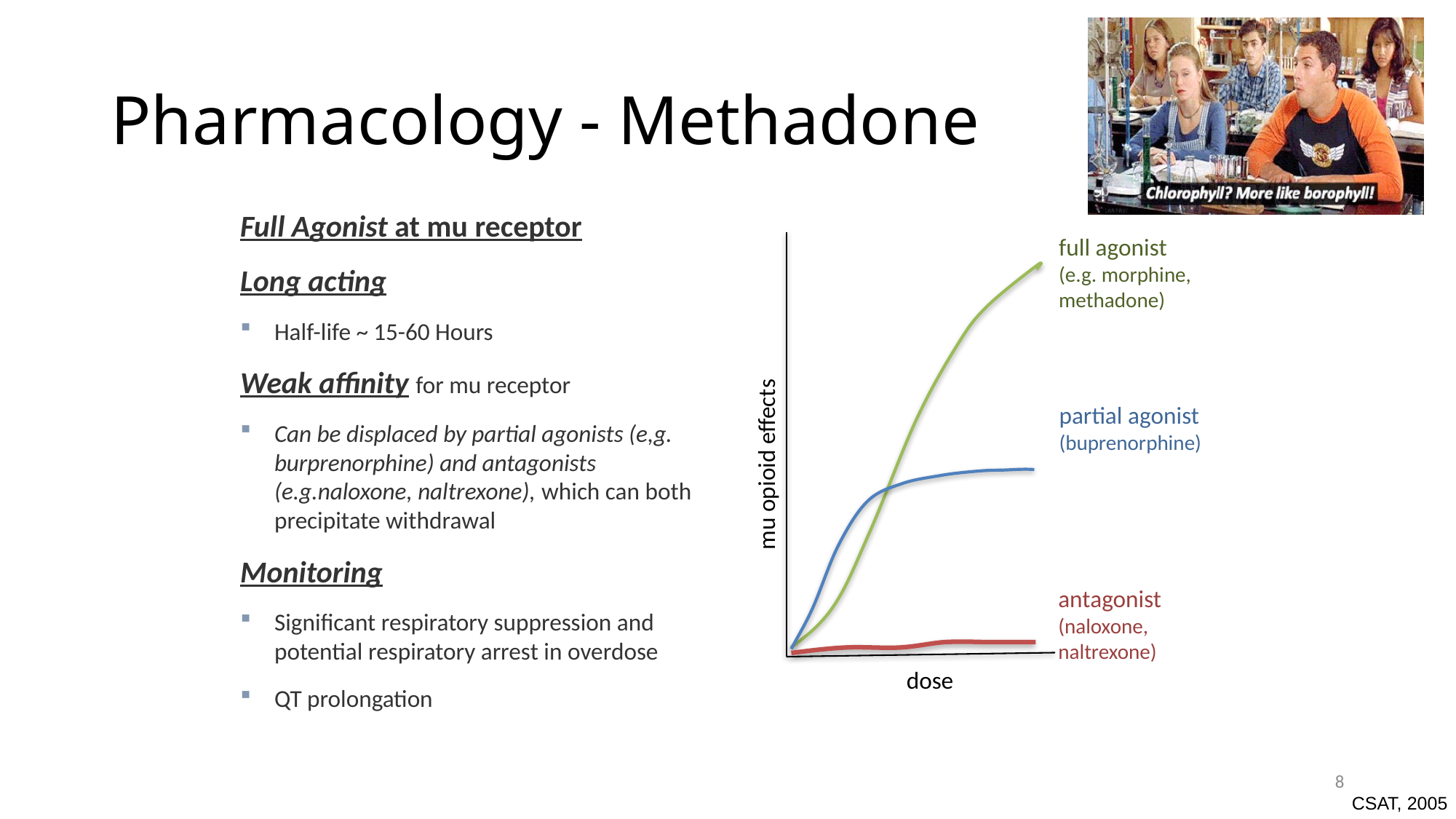

# Pharmacology - Methadone
Full Agonist at mu receptor
Long acting
Half-life ~ 15-60 Hours
Weak affinity for mu receptor
Can be displaced by partial agonists (e,g. burprenorphine) and antagonists (e.g.naloxone, naltrexone), which can both precipitate withdrawal
Monitoring
Significant respiratory suppression and potential respiratory arrest in overdose
QT prolongation
full agonist
(e.g. morphine,
methadone)
mu opioid effects
dose
partial agonist
(buprenorphine)
antagonist
(naloxone,
naltrexone)
8
CSAT, 2005

## Slide 9
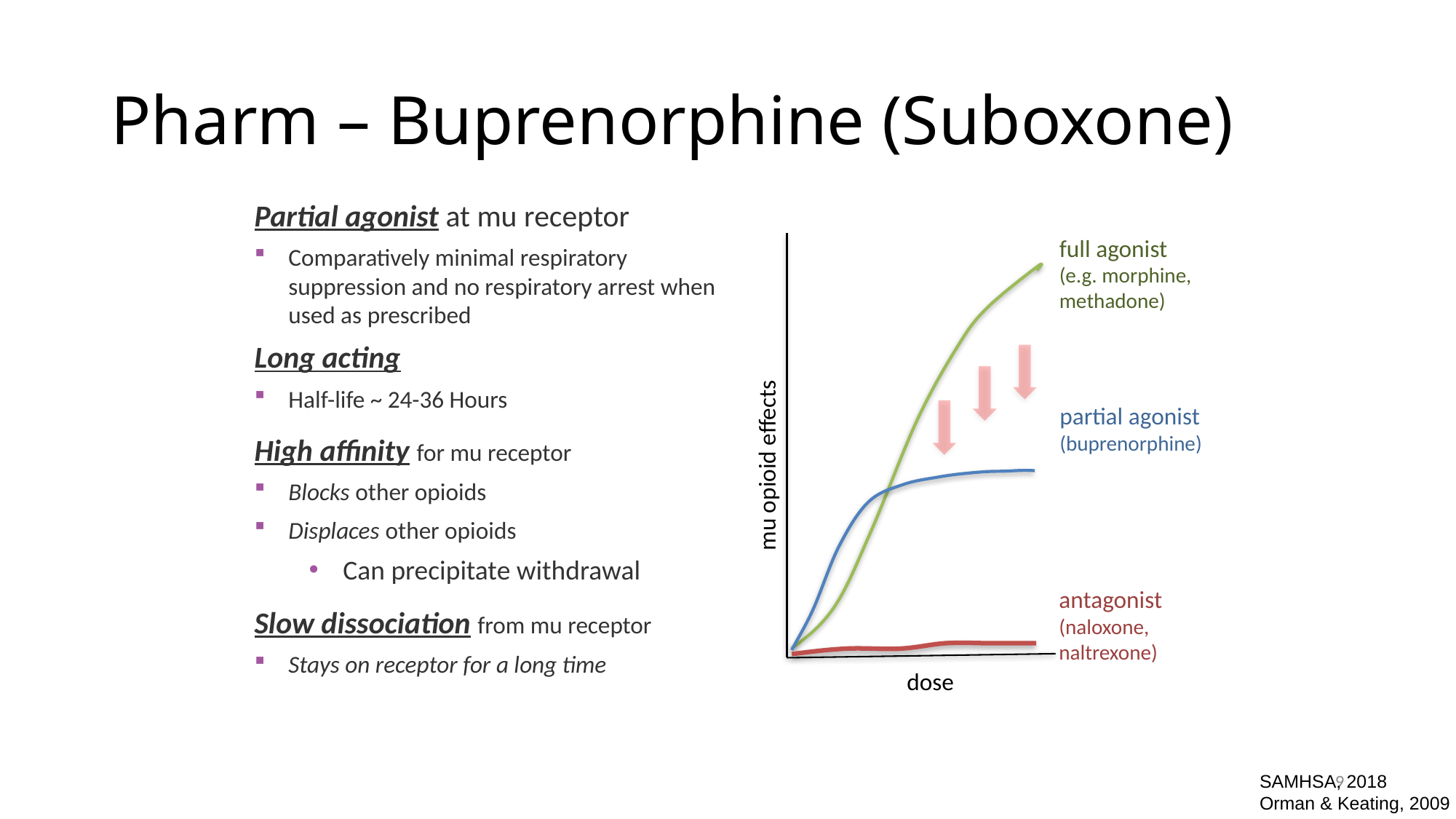

# Pharm – Buprenorphine (Suboxone)
Partial agonist at mu receptor
Comparatively minimal respiratory suppression and no respiratory arrest when used as prescribed
Long acting
Half-life ~ 24-36 Hours
High affinity for mu receptor
Blocks other opioids
Displaces other opioids
Can precipitate withdrawal
Slow dissociation from mu receptor
Stays on receptor for a long time
full agonist
(e.g. morphine,
methadone)
mu opioid effects
dose
partial agonist
(buprenorphine)
antagonist
(naloxone,
naltrexone)
9
SAMHSA, 2018
Orman & Keating, 2009

## Slide 10
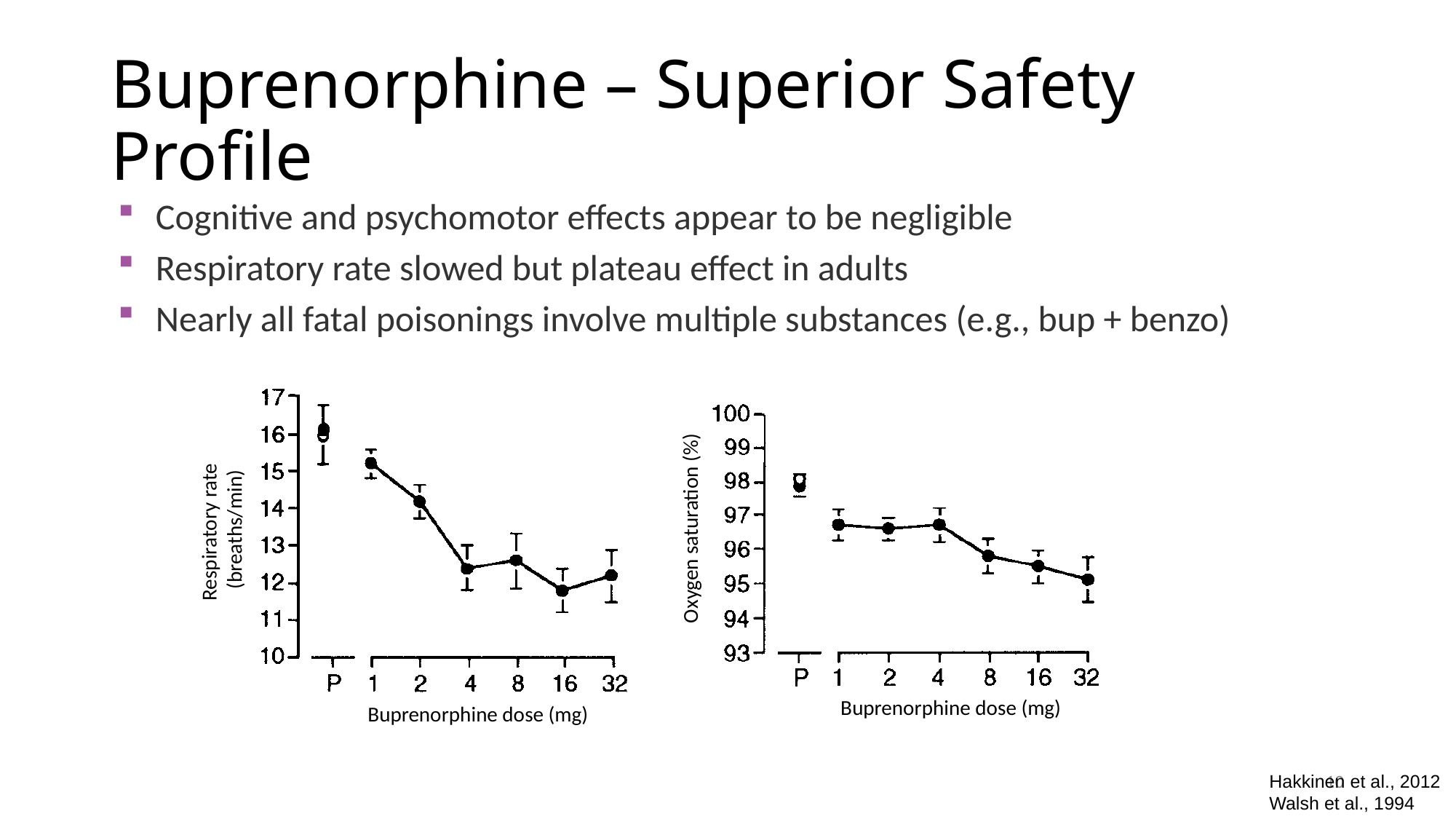

# Buprenorphine – Superior Safety Profile
Cognitive and psychomotor effects appear to be negligible
Respiratory rate slowed but plateau effect in adults
Nearly all fatal poisonings involve multiple substances (e.g., bup + benzo)
Respiratory rate
(breaths/min)
Buprenorphine dose (mg)
Oxygen saturation (%)
Buprenorphine dose (mg)
10
Hakkinen et al., 2012
Walsh et al., 1994

## Slide 11
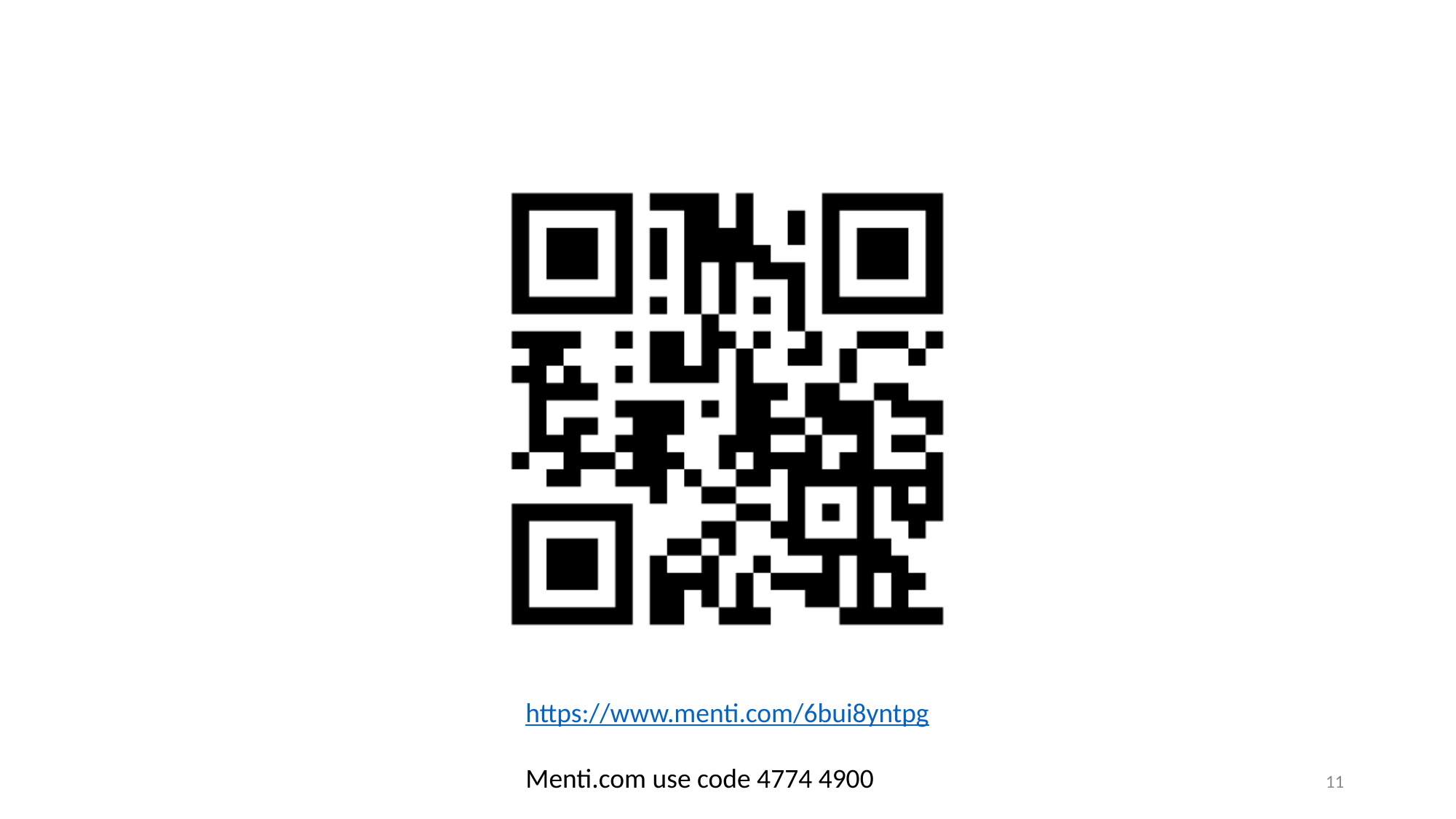

https://www.menti.com/6bui8yntpg
Menti.com use code 4774 4900
11

## Slide 12
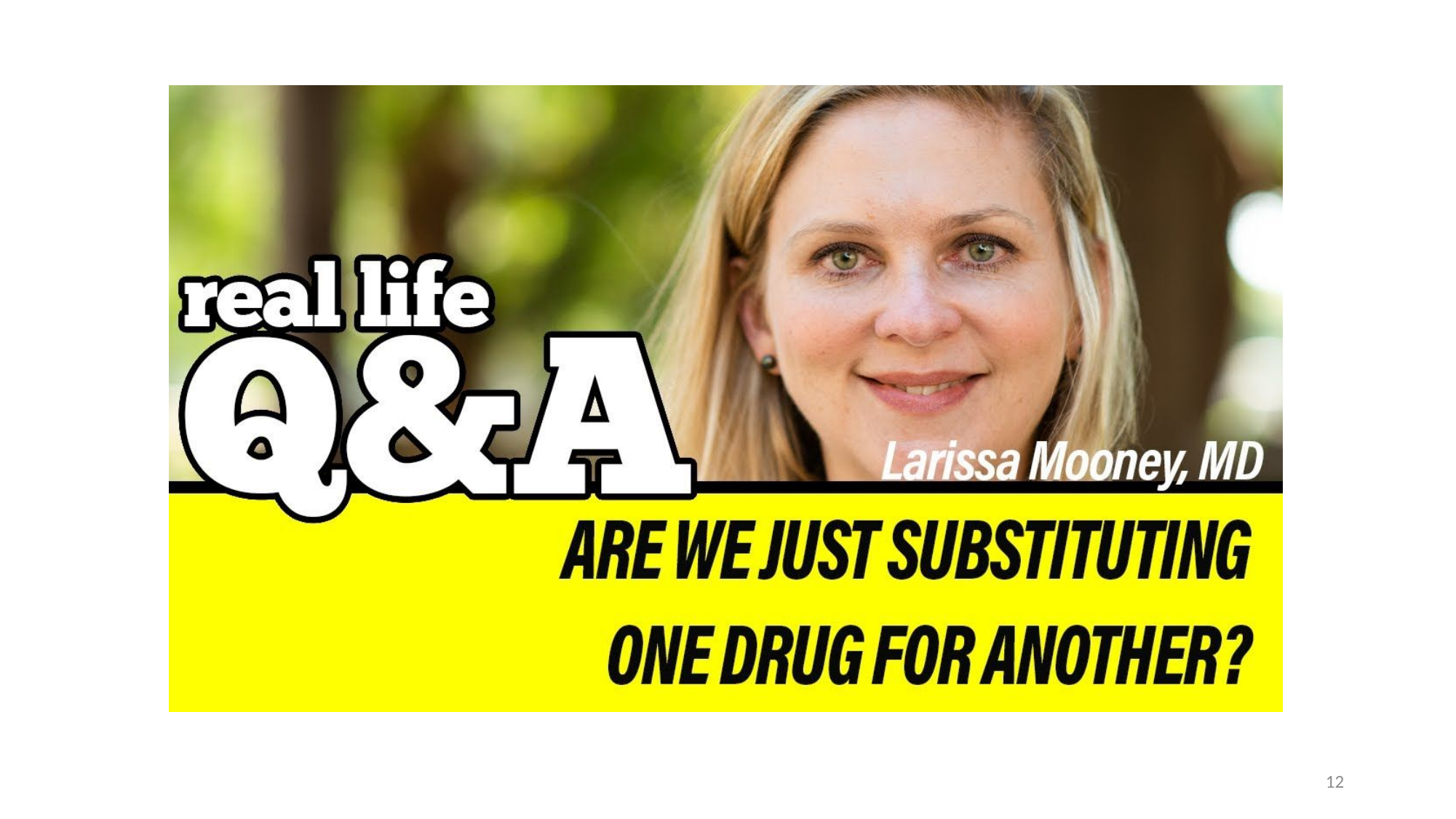

12

## Slide 13
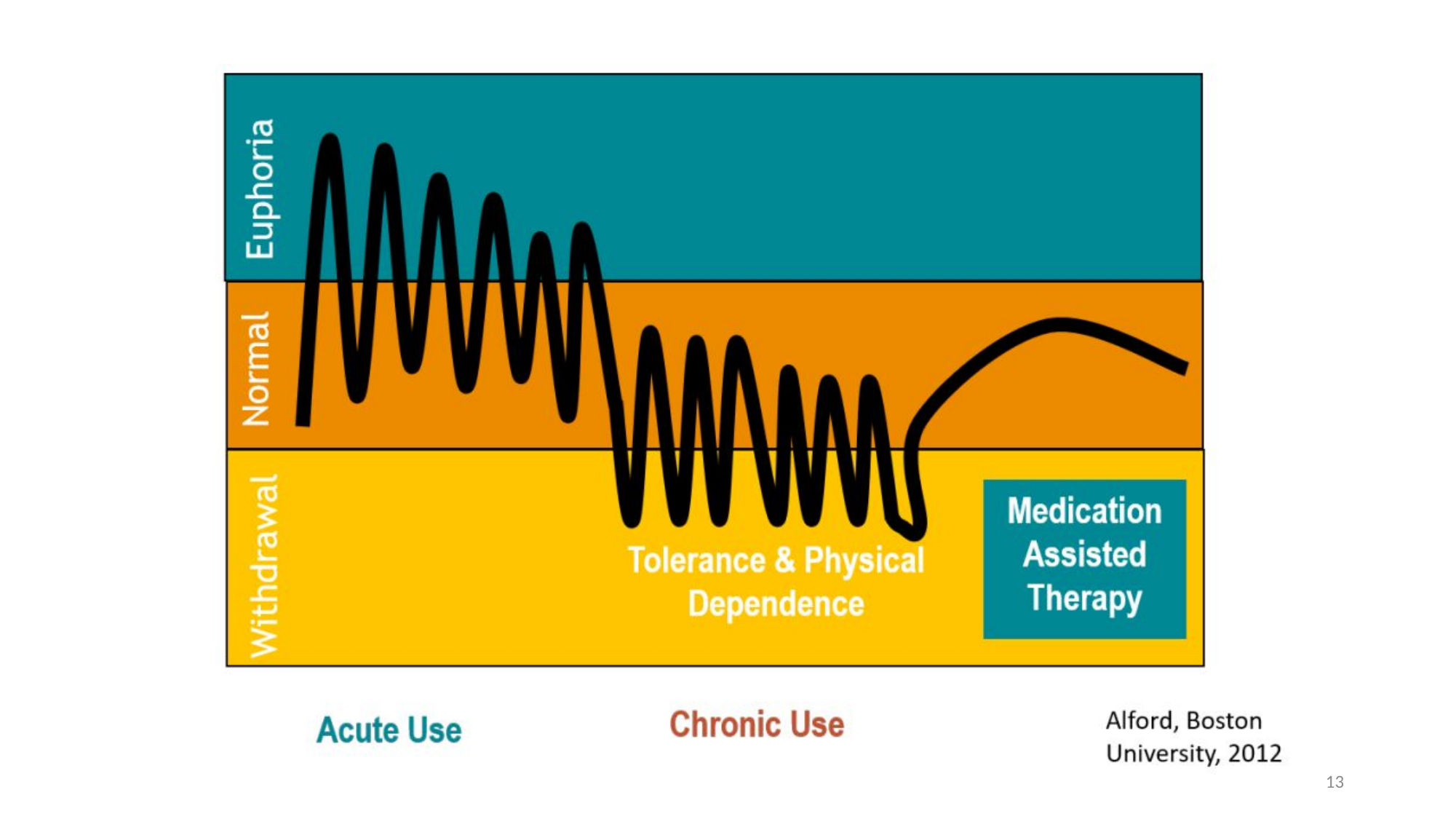

13

## Slide 14
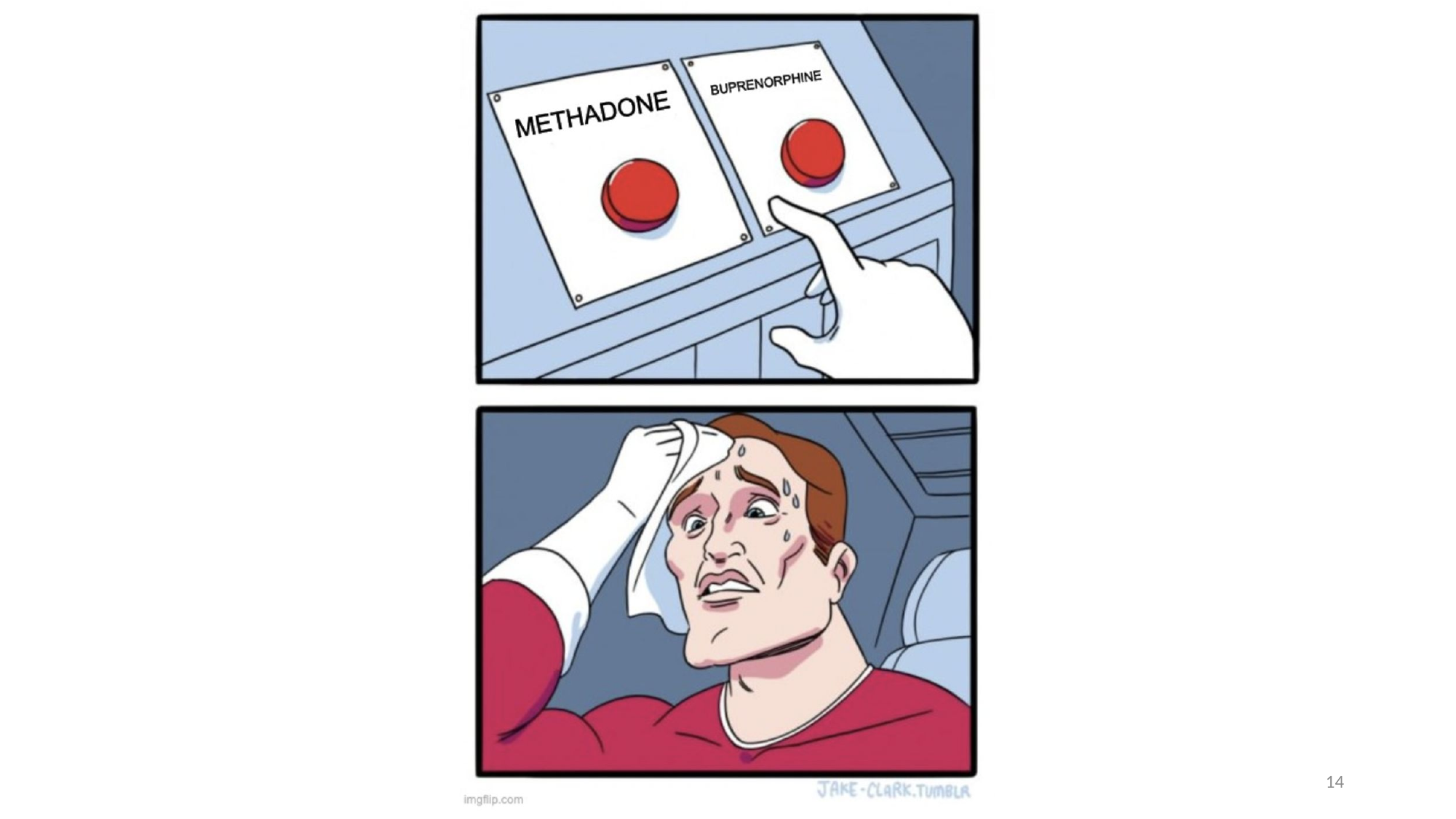

14

## Slide 15
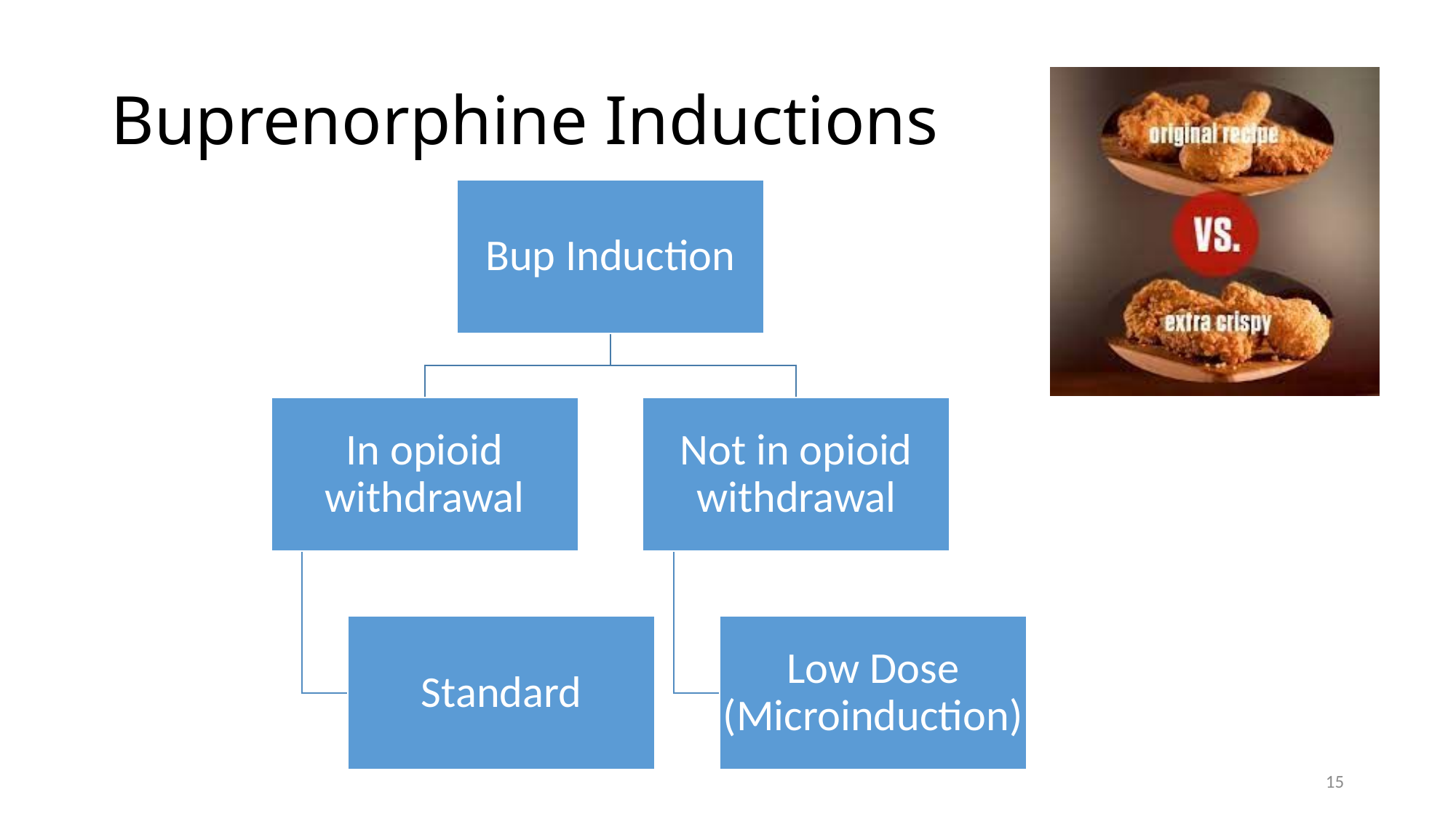

# Buprenorphine Inductions
15

## Slide 16
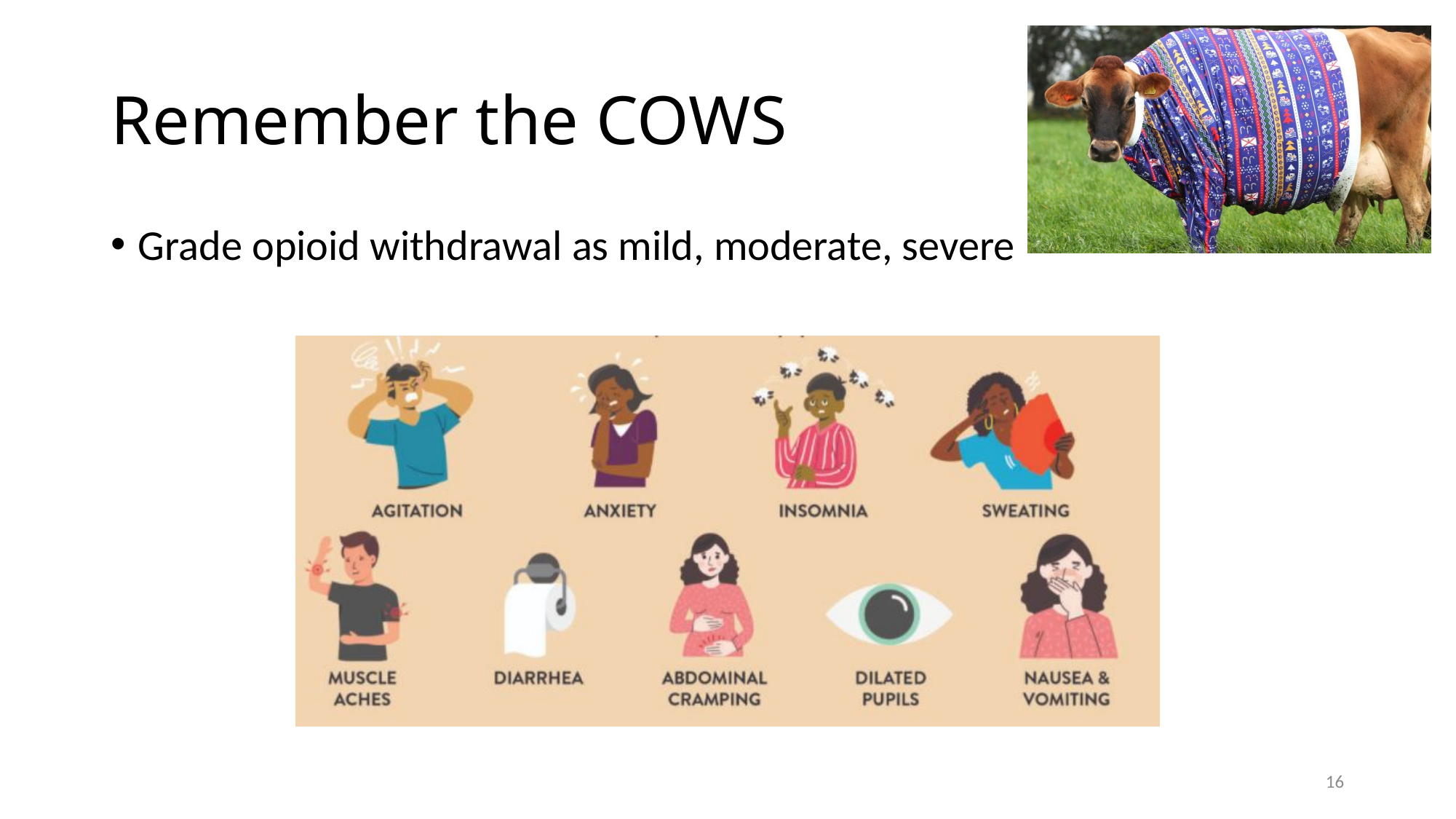

# Remember the COWS
Grade opioid withdrawal as mild, moderate, severe
16

## Slide 17
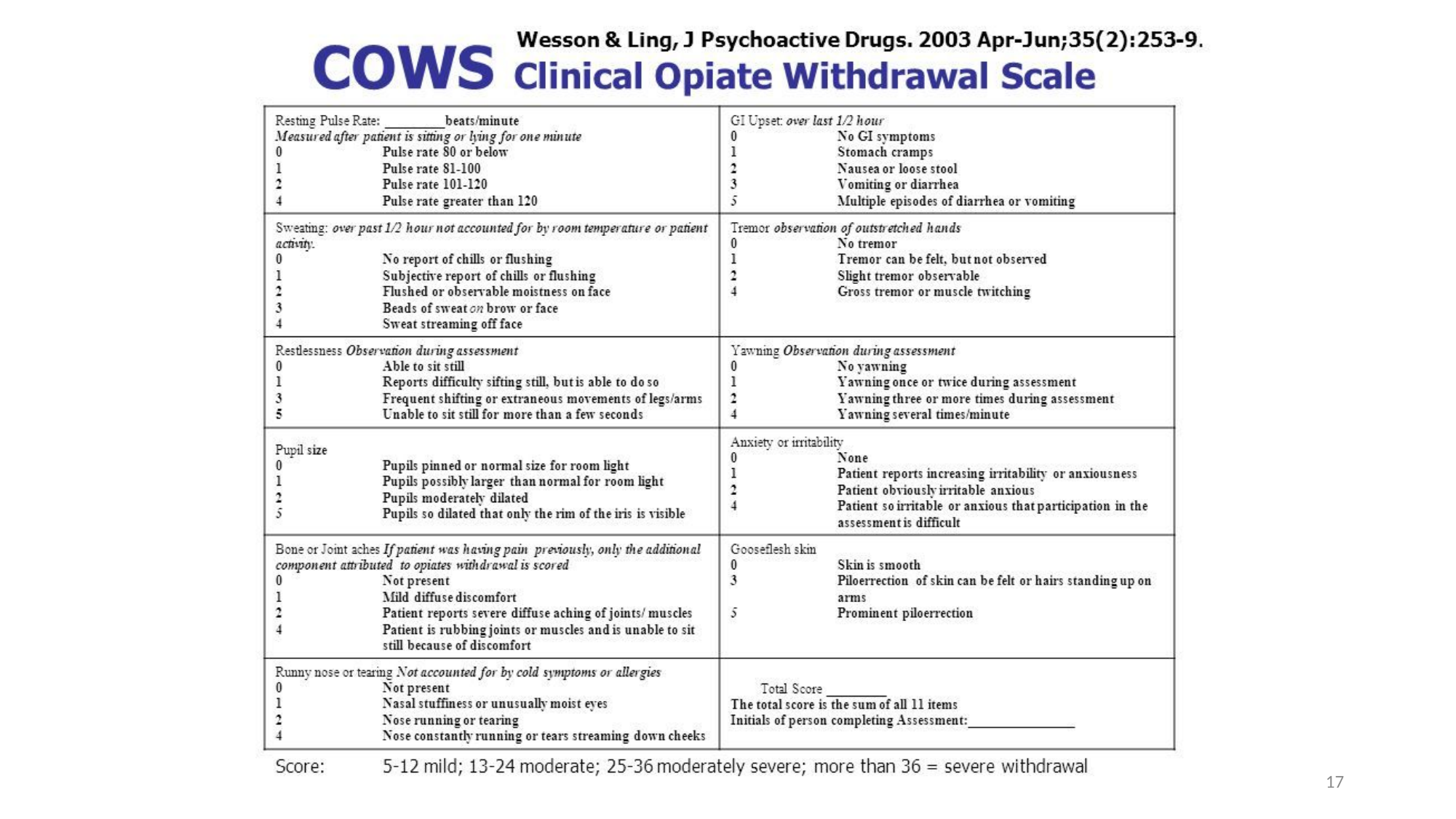

17

## Slide 18
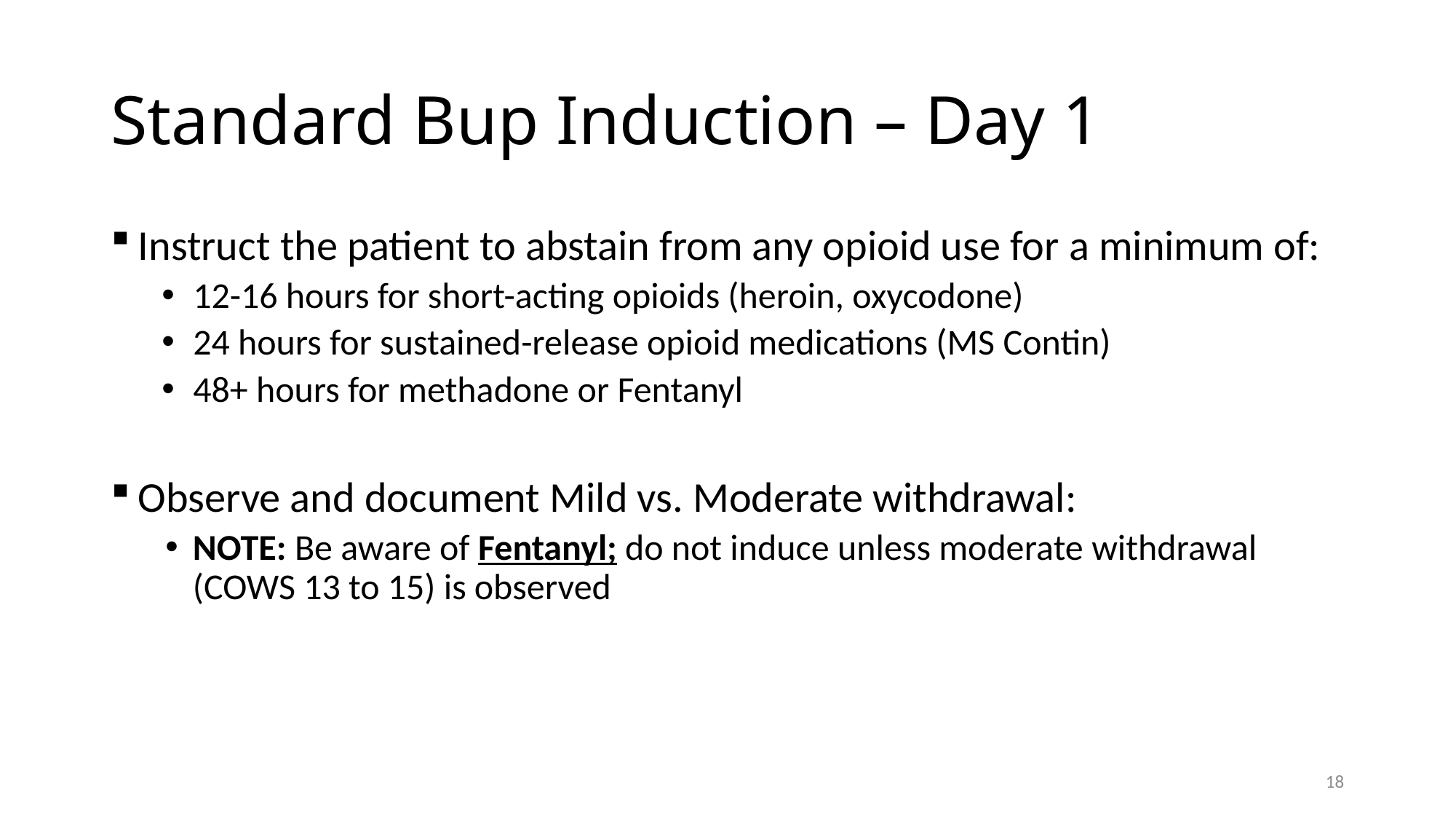

# Standard Bup Induction – Day 1
Instruct the patient to abstain from any opioid use for a minimum of:
12-16 hours for short-acting opioids (heroin, oxycodone)
24 hours for sustained-release opioid medications (MS Contin)
48+ hours for methadone or Fentanyl
Observe and document Mild vs. Moderate withdrawal:
NOTE: Be aware of Fentanyl; do not induce unless moderate withdrawal (COWS 13 to 15) is observed
18

## Slide 19
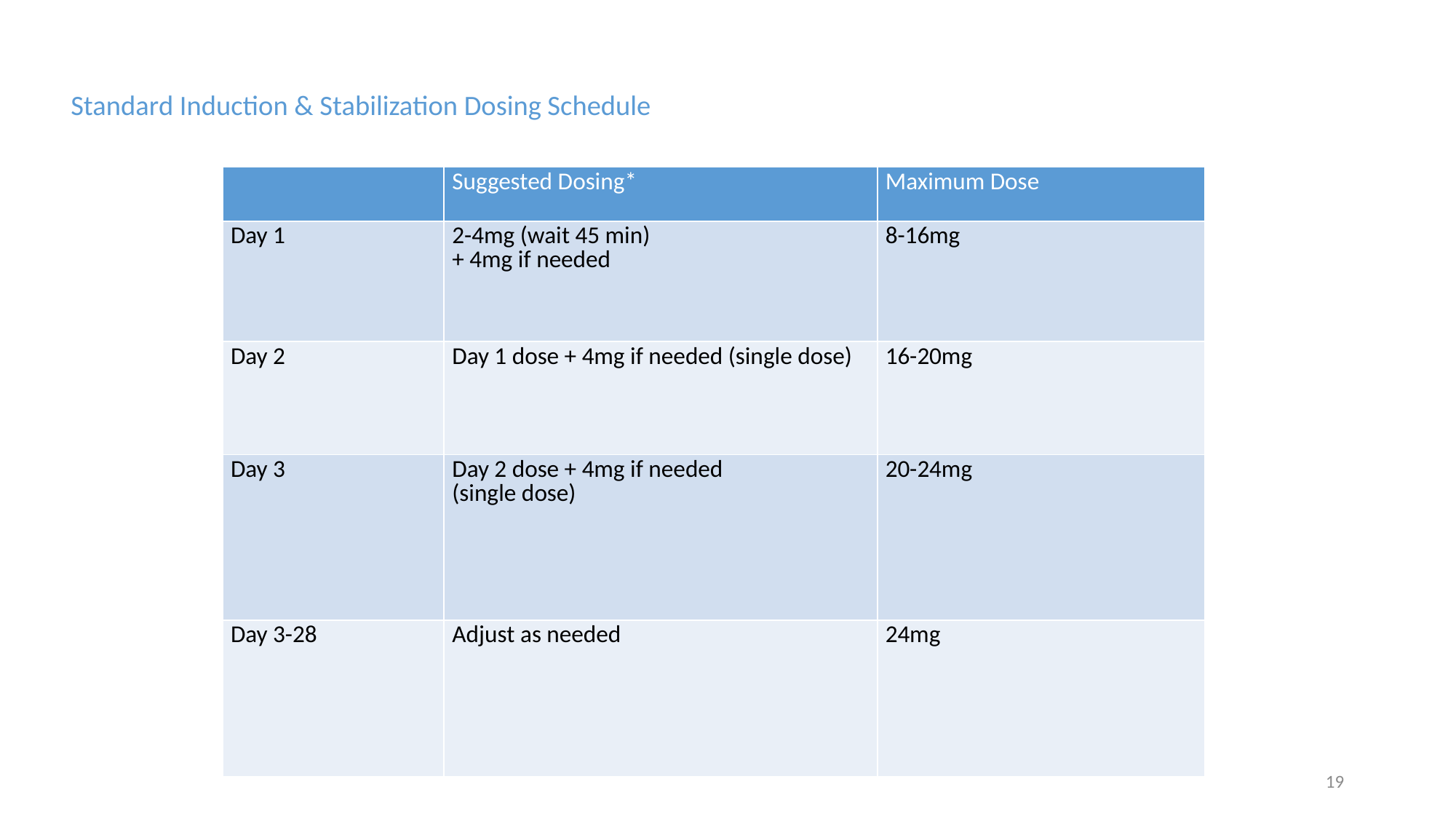

# Standard Induction & Stabilization Dosing Schedule
| | Suggested Dosing\* | Maximum Dose |
| --- | --- | --- |
| Day 1 | 2-4mg (wait 45 min) + 4mg if needed | 8-16mg |
| Day 2 | Day 1 dose + 4mg if needed (single dose) | 16-20mg |
| Day 3 | Day 2 dose + 4mg if needed (single dose) | 20-24mg |
| Day 3-28 | Adjust as needed | 24mg |
19

## Slide 20
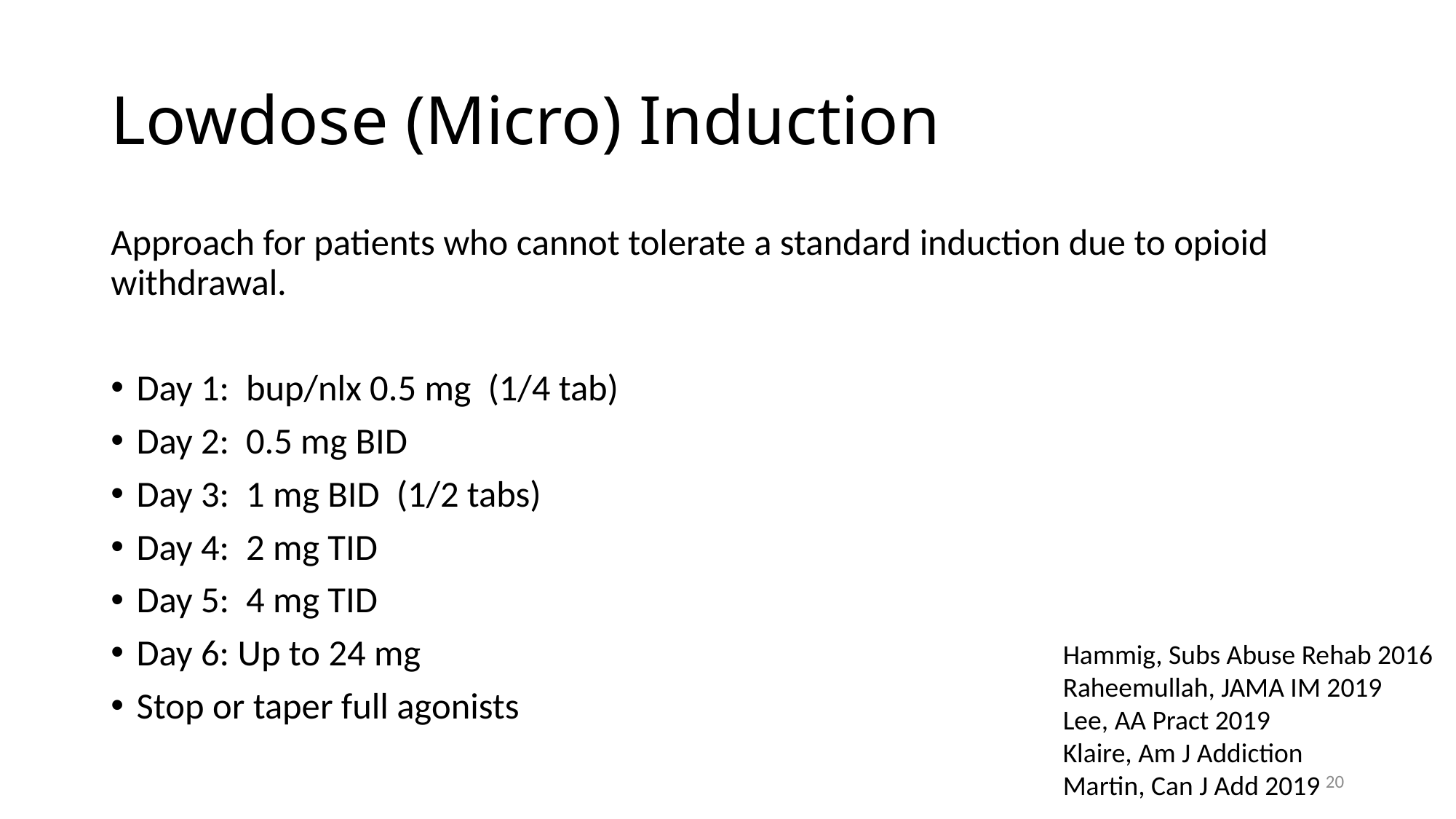

# Lowdose (Micro) Induction
Approach for patients who cannot tolerate a standard induction due to opioid withdrawal.
Day 1:  bup/nlx 0.5 mg  (1/4 tab)
Day 2:  0.5 mg BID
Day 3:  1 mg BID  (1/2 tabs)
Day 4:  2 mg TID
Day 5:  4 mg TID
Day 6: Up to 24 mg
Stop or taper full agonists
Hammig, Subs Abuse Rehab 2016
Raheemullah, JAMA IM 2019
Lee, AA Pract 2019
Klaire, Am J Addiction
Martin, Can J Add 2019
20

## Slide 21
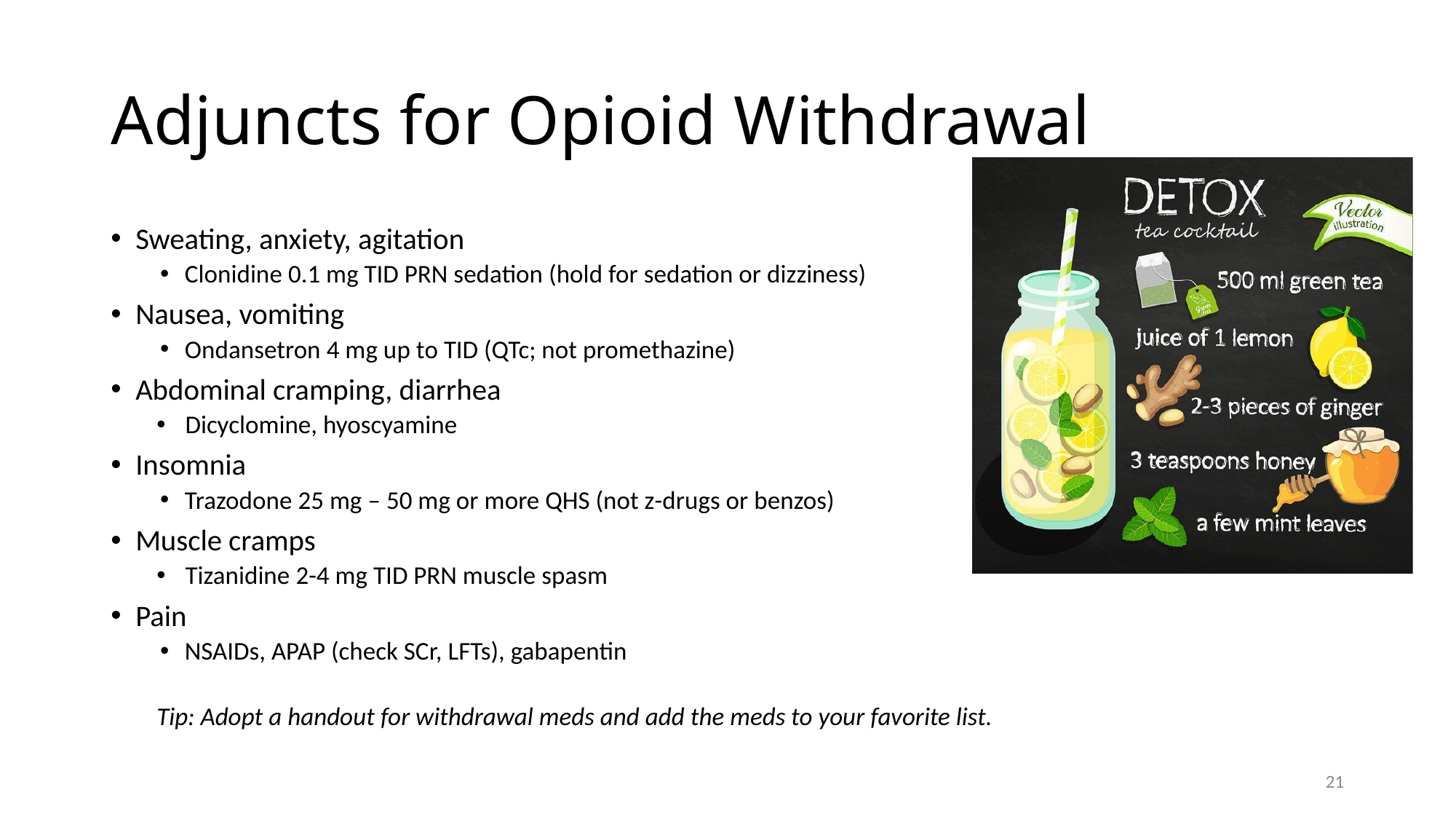

# Adjuncts for Opioid Withdrawal
Sweating, anxiety, agitation
Clonidine 0.1 mg TID PRN sedation (hold for sedation or dizziness)
Nausea, vomiting
Ondansetron 4 mg up to TID (QTc; not promethazine)
Abdominal cramping, diarrhea
Dicyclomine, hyoscyamine
Insomnia
Trazodone 25 mg – 50 mg or more QHS (not z-drugs or benzos)
Muscle cramps
Tizanidine 2-4 mg TID PRN muscle spasm
Pain
NSAIDs, APAP (check SCr, LFTs), gabapentin
Tip: Adopt a handout for withdrawal meds and add the meds to your favorite list.
21

## Slide 22
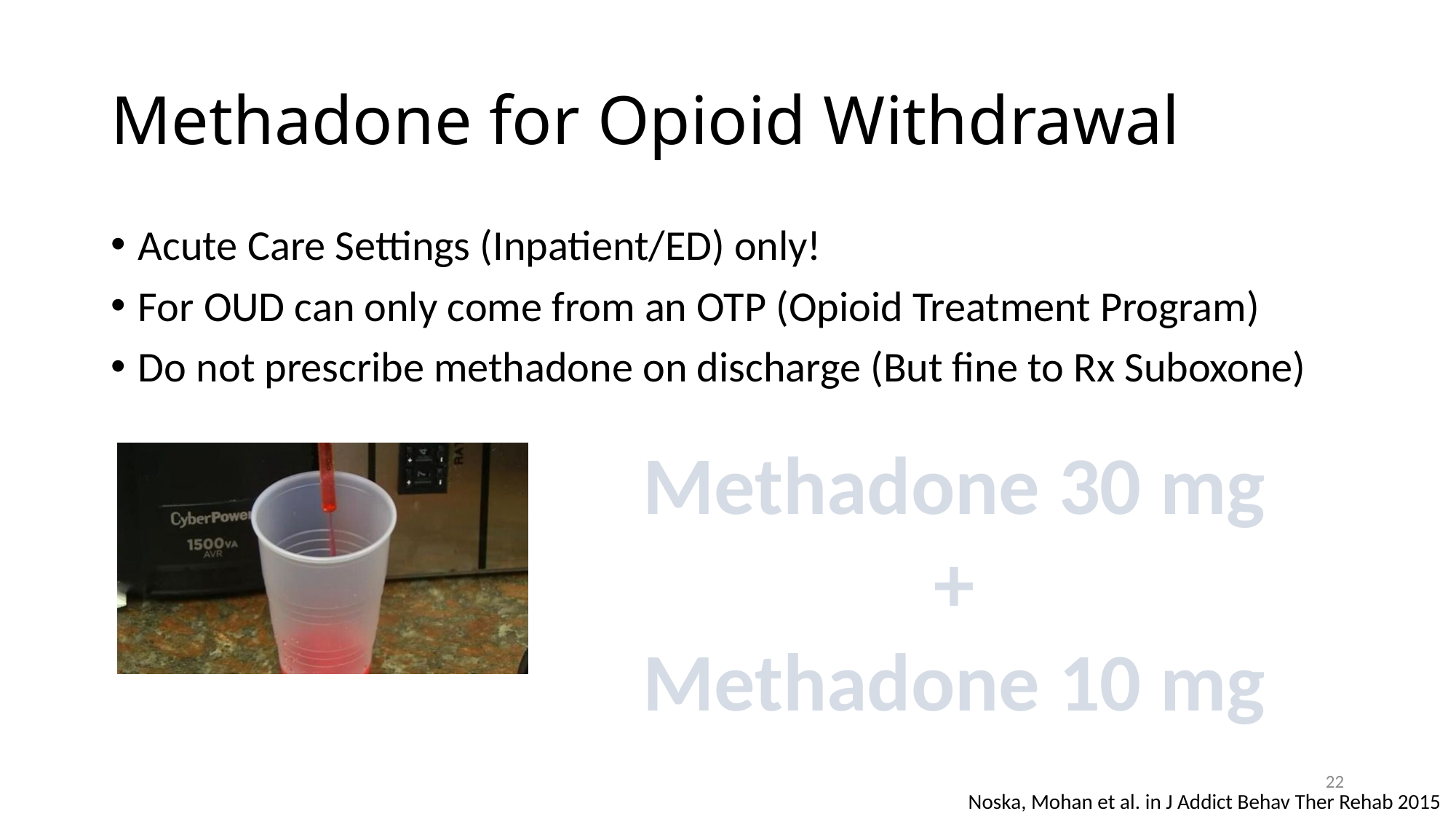

# Methadone for Opioid Withdrawal
Acute Care Settings (Inpatient/ED) only!
For OUD can only come from an OTP (Opioid Treatment Program)
Do not prescribe methadone on discharge (But fine to Rx Suboxone)
Methadone 30 mg
+
Methadone 10 mg
22
Noska, Mohan et al. in J Addict Behav Ther Rehab 2015

## Slide 23
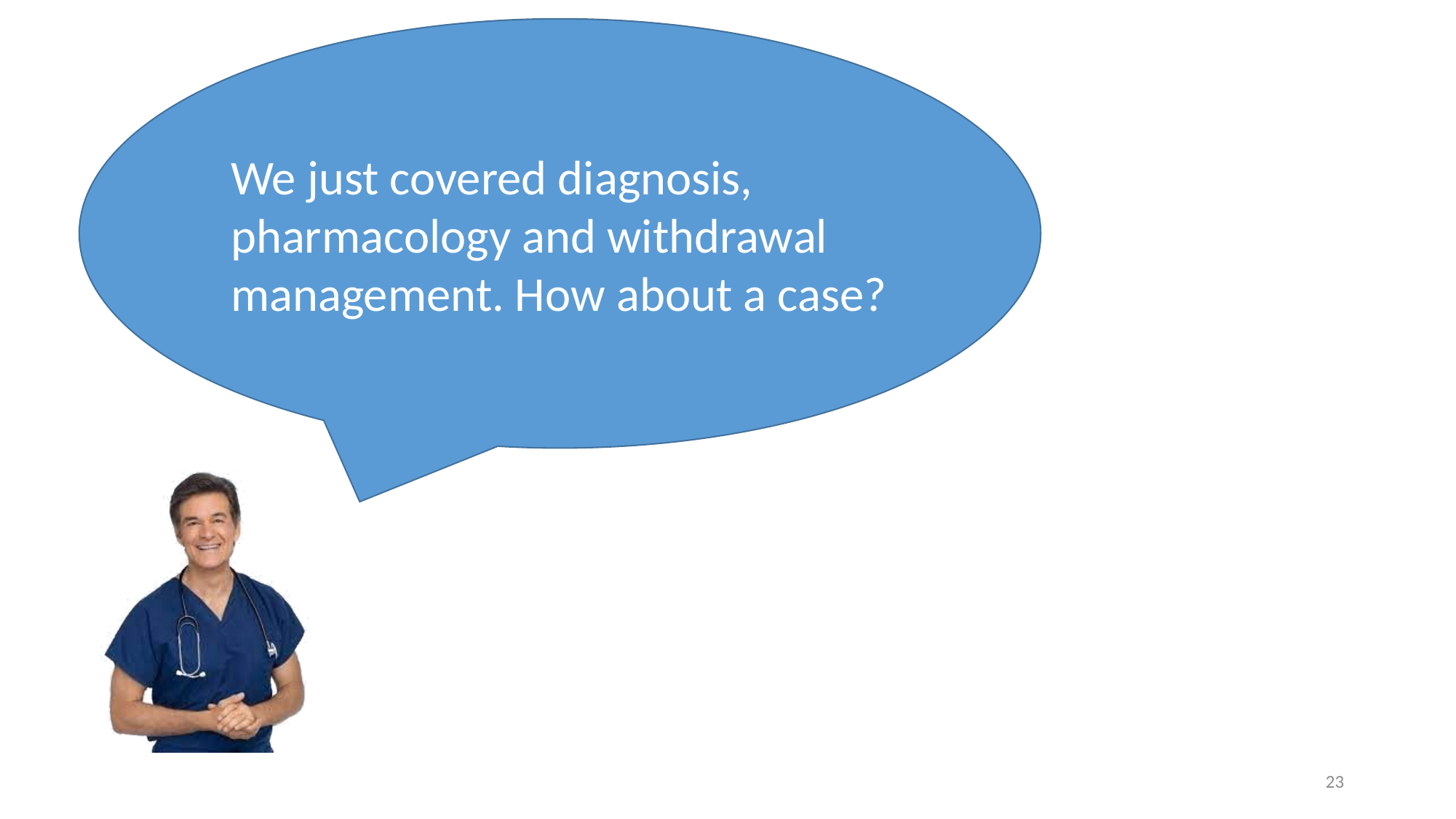

We just covered diagnosis, pharmacology and withdrawal management. How about a case?
23

## Slide 24
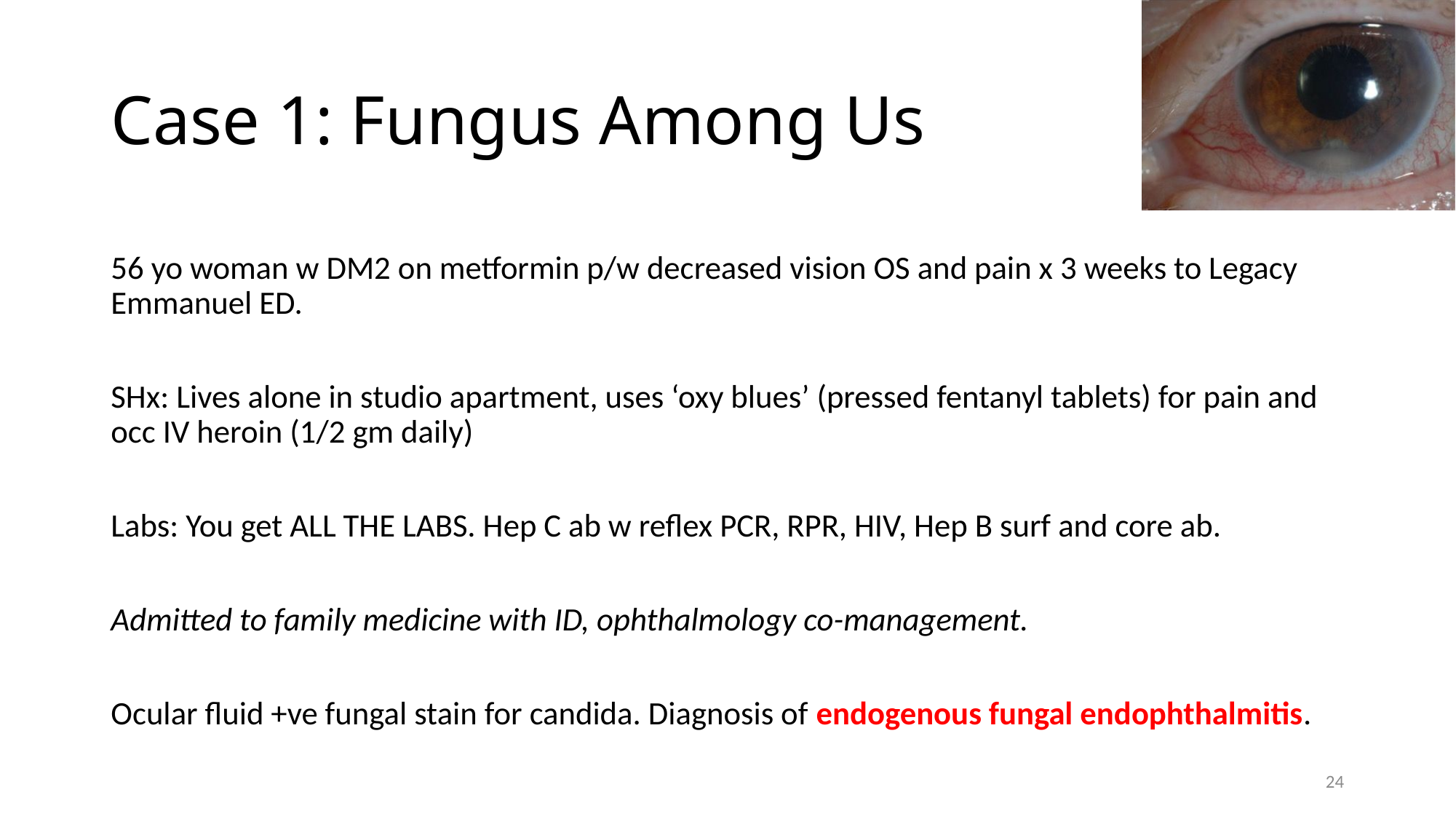

# Case 1: Fungus Among Us
56 yo woman w DM2 on metformin p/w decreased vision OS and pain x 3 weeks to Legacy Emmanuel ED.
SHx: Lives alone in studio apartment, uses ‘oxy blues’ (pressed fentanyl tablets) for pain and occ IV heroin (1/2 gm daily)
Labs: You get ALL THE LABS. Hep C ab w reflex PCR, RPR, HIV, Hep B surf and core ab.
Admitted to family medicine with ID, ophthalmology co-management.
Ocular fluid +ve fungal stain for candida. Diagnosis of endogenous fungal endophthalmitis.
24

## Slide 25
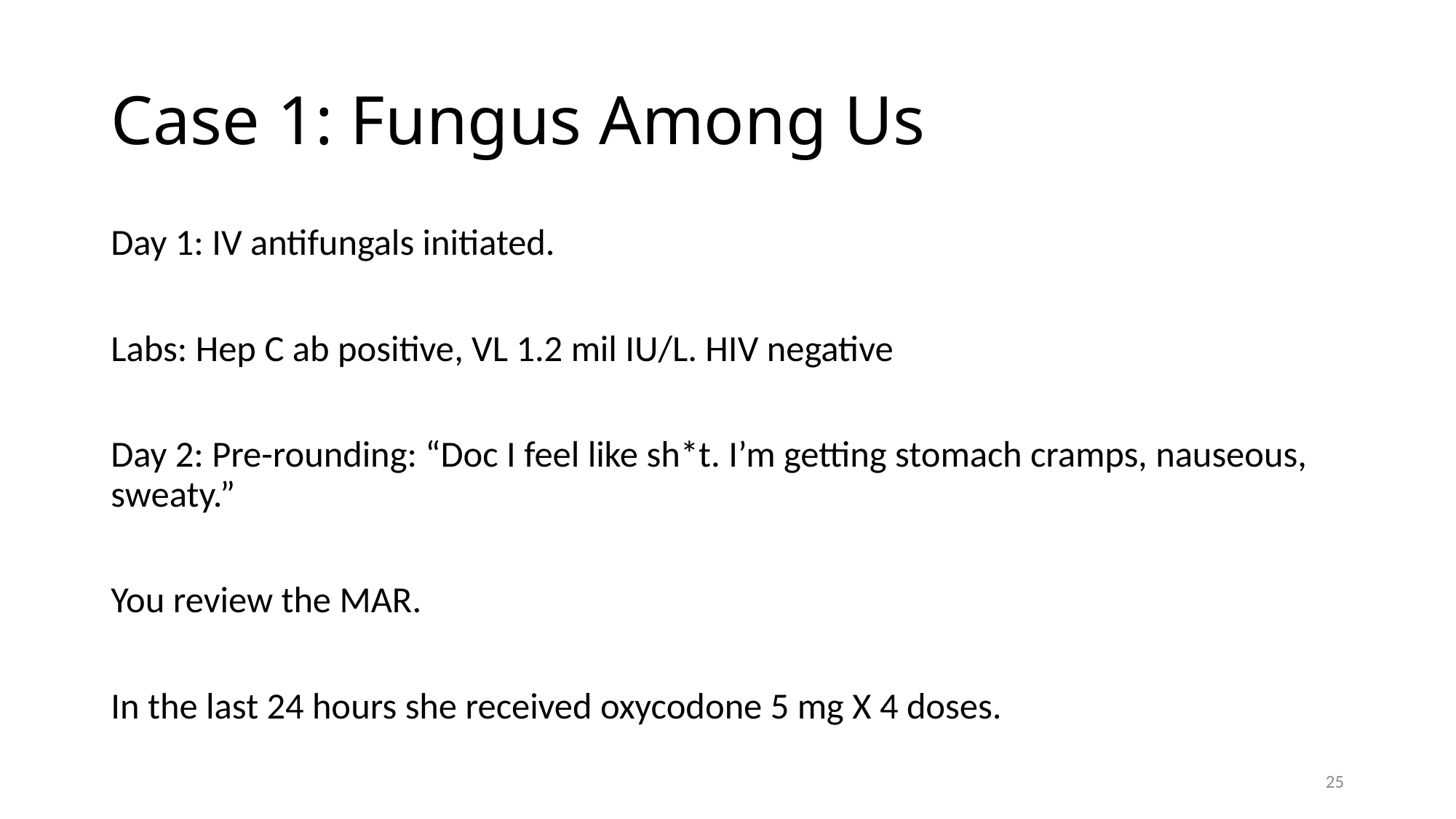

# Case 1: Fungus Among Us
Day 1: IV antifungals initiated.
Labs: Hep C ab positive, VL 1.2 mil IU/L. HIV negative
Day 2: Pre-rounding: “Doc I feel like sh*t. I’m getting stomach cramps, nauseous, sweaty.”
You review the MAR.
In the last 24 hours she received oxycodone 5 mg X 4 doses.
25

## Slide 26
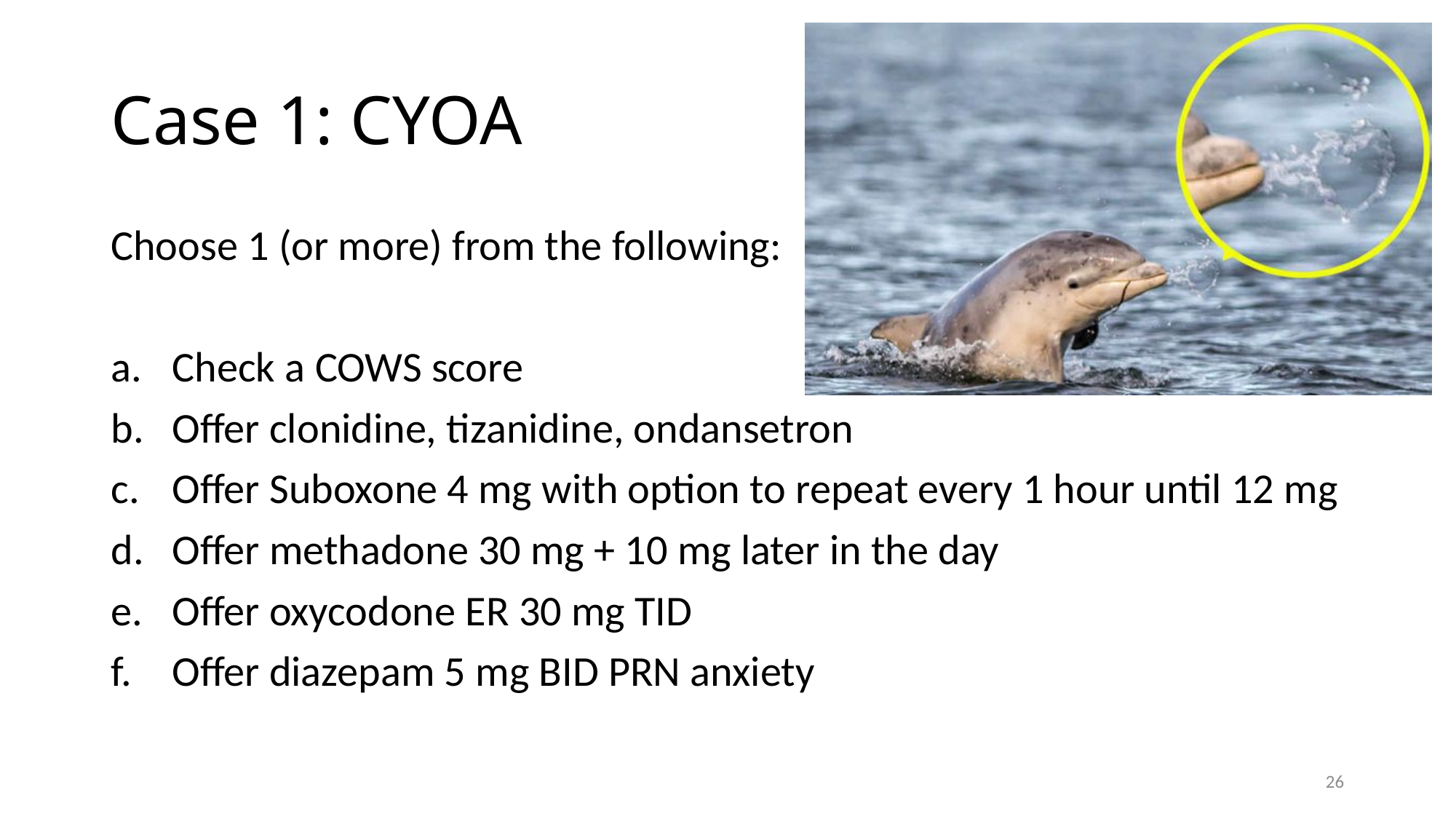

# Case 1: CYOA
Choose 1 (or more) from the following:
Check a COWS score
Offer clonidine, tizanidine, ondansetron
Offer Suboxone 4 mg with option to repeat every 1 hour until 12 mg
Offer methadone 30 mg + 10 mg later in the day
Offer oxycodone ER 30 mg TID
Offer diazepam 5 mg BID PRN anxiety
26

## Slide 27
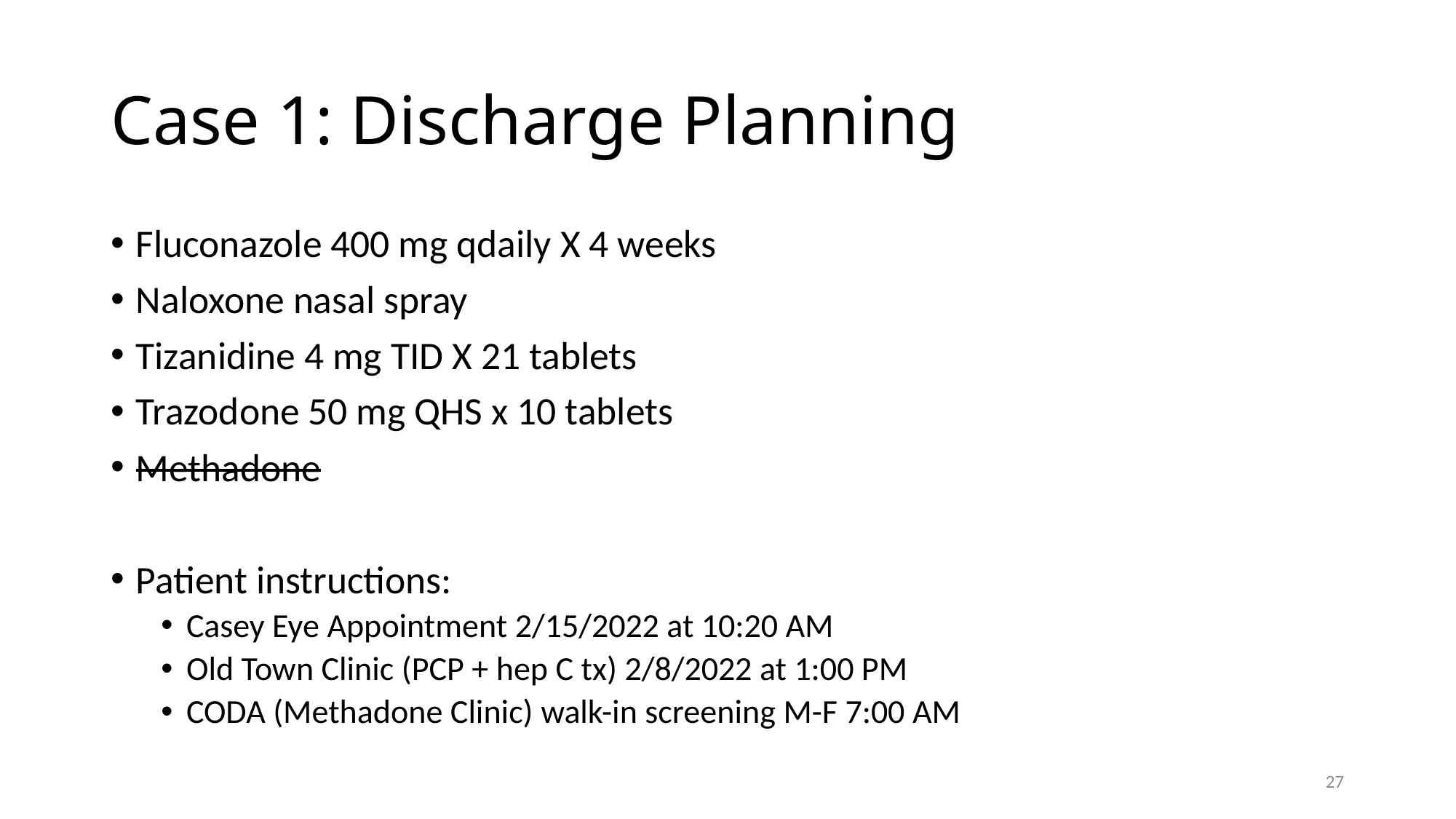

# Case 1: Discharge Planning
Fluconazole 400 mg qdaily X 4 weeks
Naloxone nasal spray
Tizanidine 4 mg TID X 21 tablets
Trazodone 50 mg QHS x 10 tablets
Methadone
Patient instructions:
Casey Eye Appointment 2/15/2022 at 10:20 AM
Old Town Clinic (PCP + hep C tx) 2/8/2022 at 1:00 PM
CODA (Methadone Clinic) walk-in screening M-F 7:00 AM
27

## Slide 28
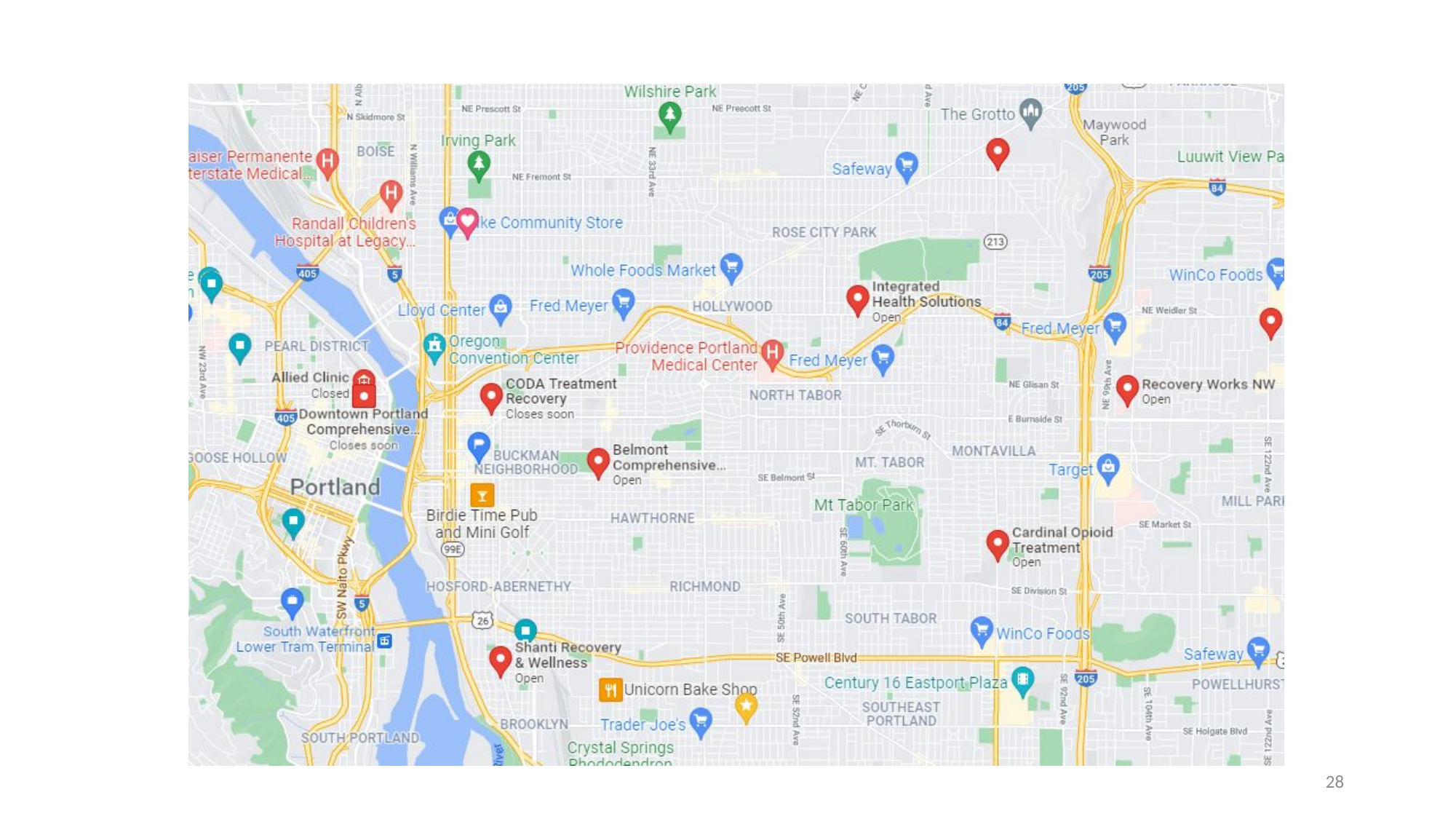

28

## Slide 29
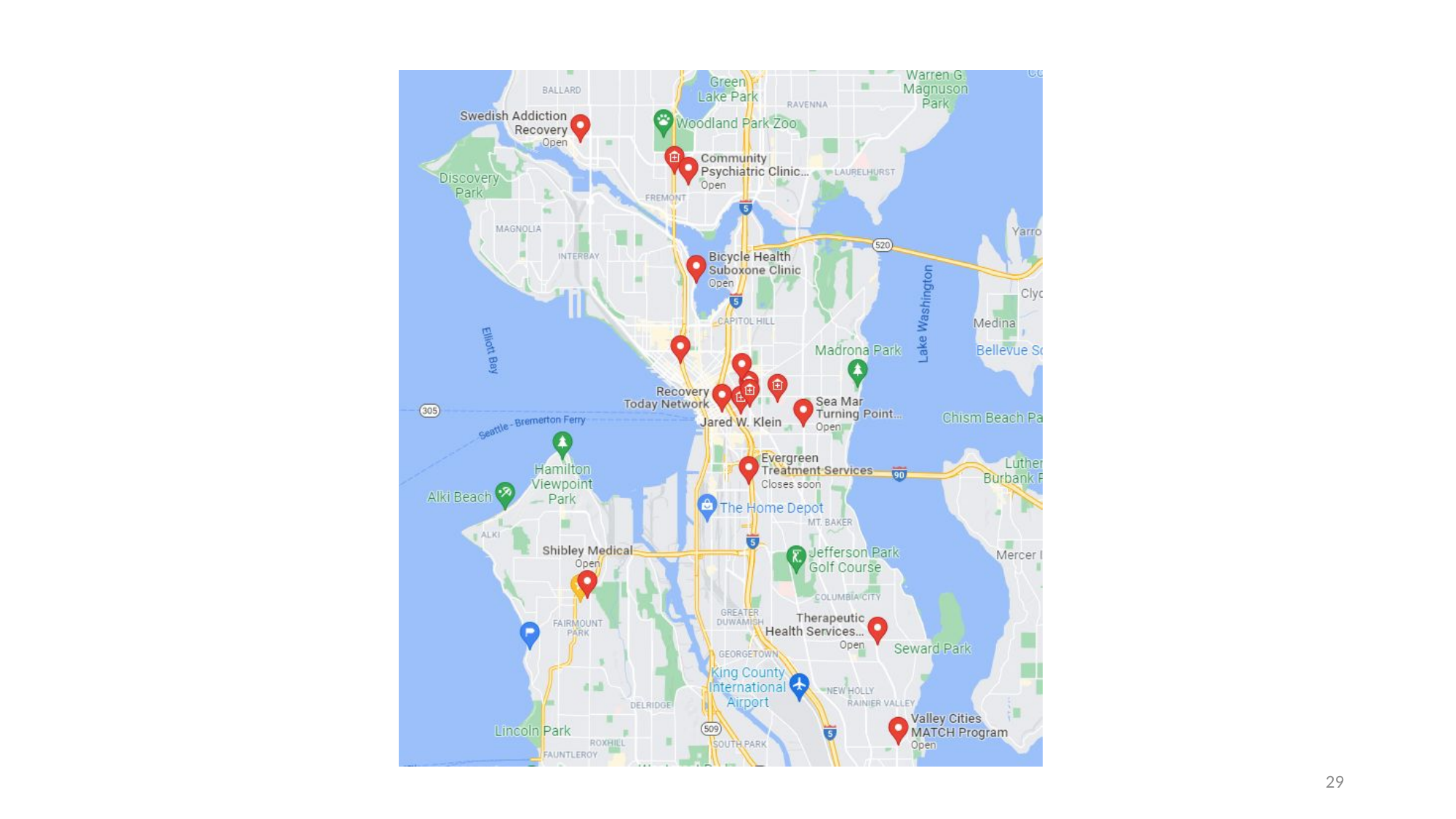

29

## Slide 30
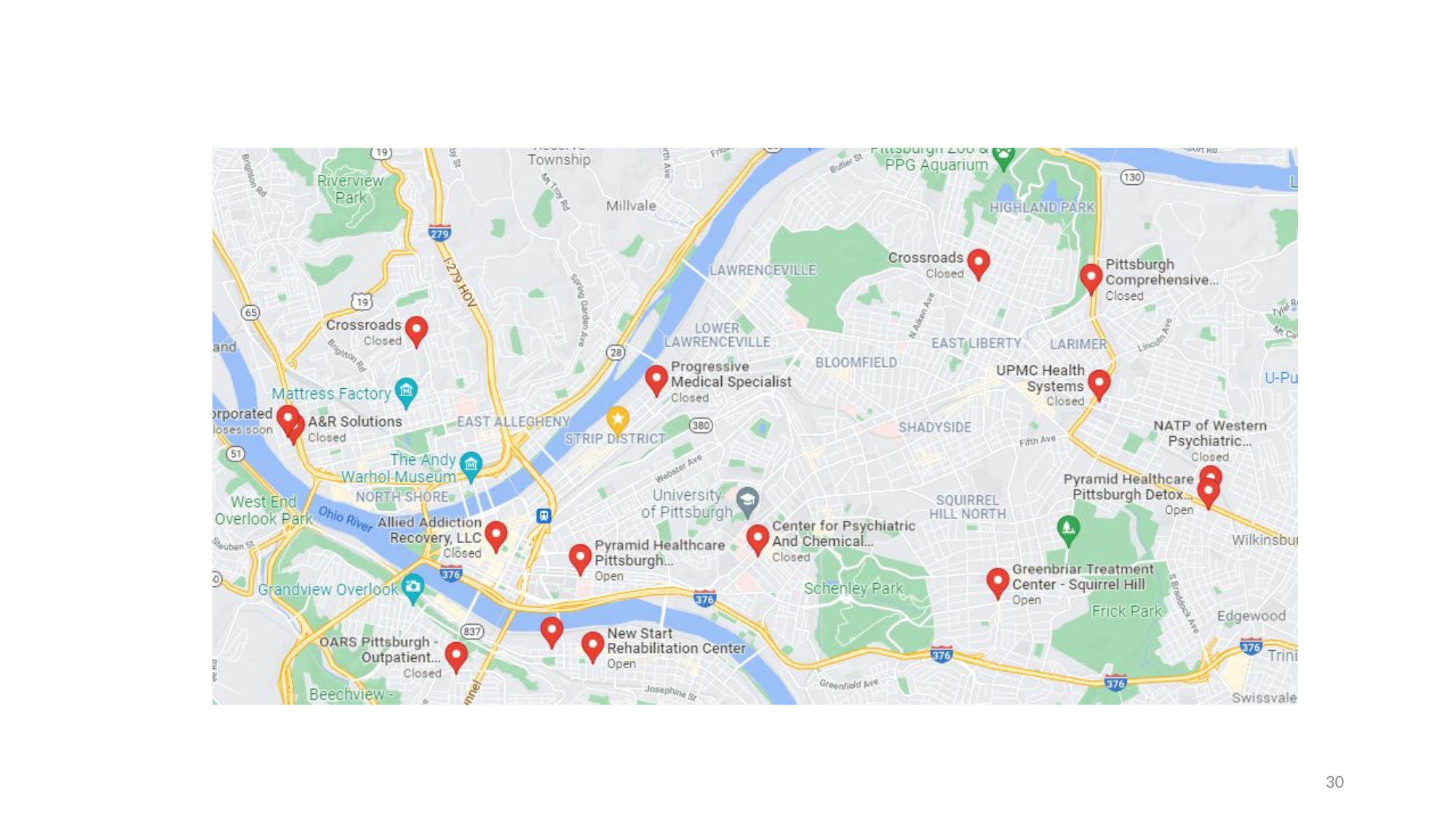

30

## Slide 31
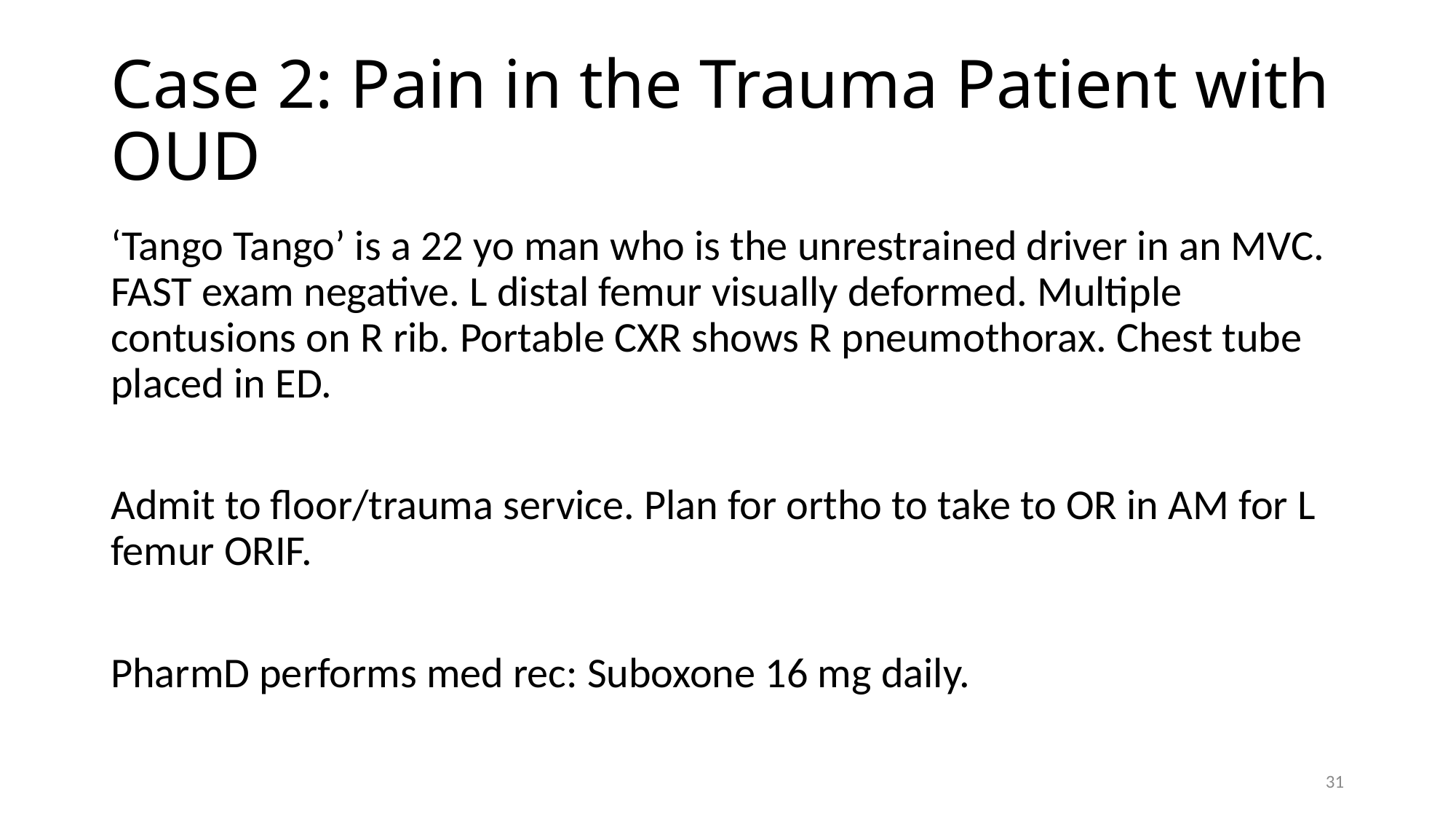

# Case 2: Pain in the Trauma Patient with OUD
‘Tango Tango’ is a 22 yo man who is the unrestrained driver in an MVC. FAST exam negative. L distal femur visually deformed. Multiple contusions on R rib. Portable CXR shows R pneumothorax. Chest tube placed in ED.
Admit to floor/trauma service. Plan for ortho to take to OR in AM for L femur ORIF.
PharmD performs med rec: Suboxone 16 mg daily.
31

## Slide 32
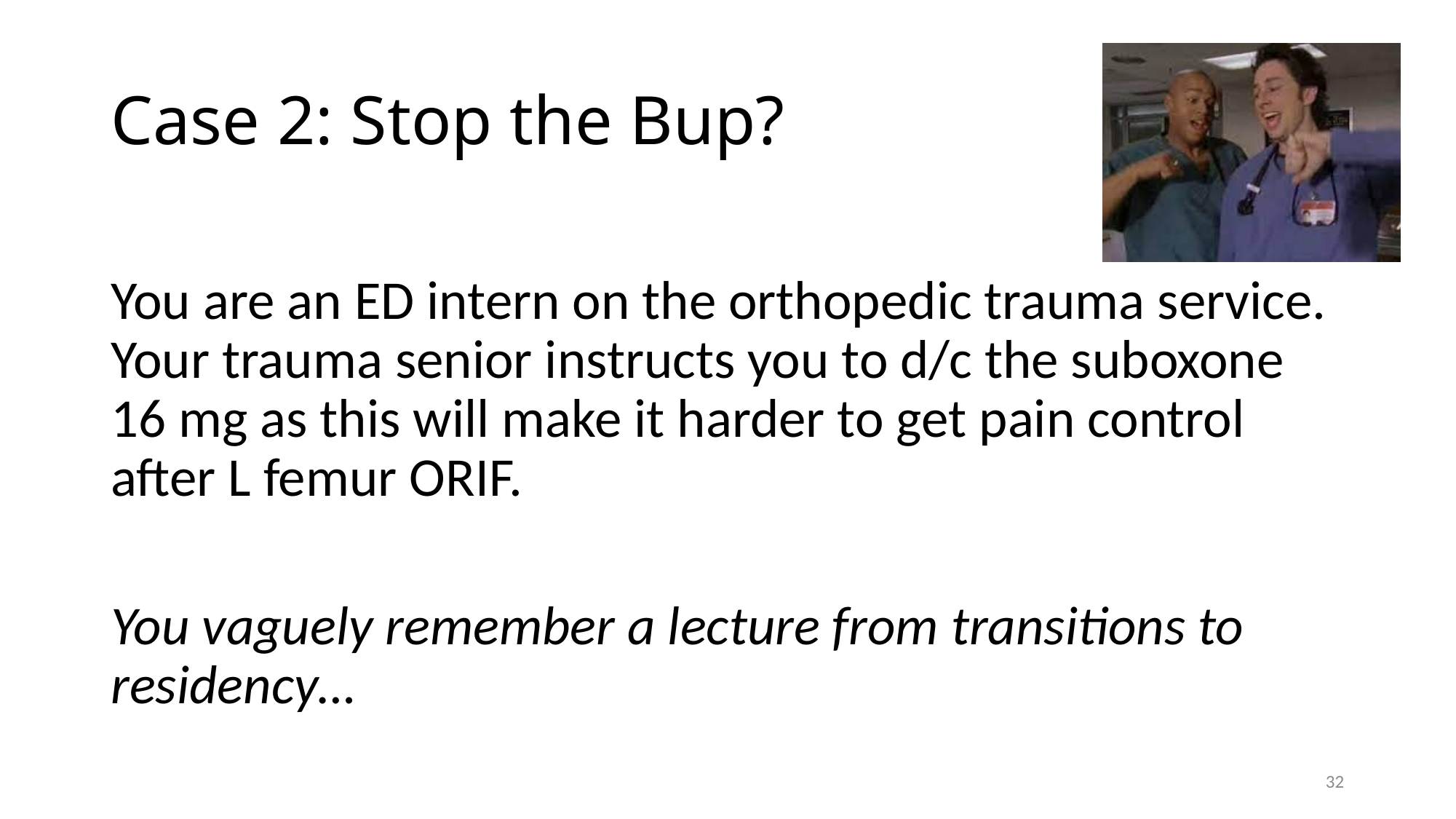

# Case 2: Stop the Bup?
You are an ED intern on the orthopedic trauma service. Your trauma senior instructs you to d/c the suboxone 16 mg as this will make it harder to get pain control after L femur ORIF.
You vaguely remember a lecture from transitions to residency…
32

## Slide 33
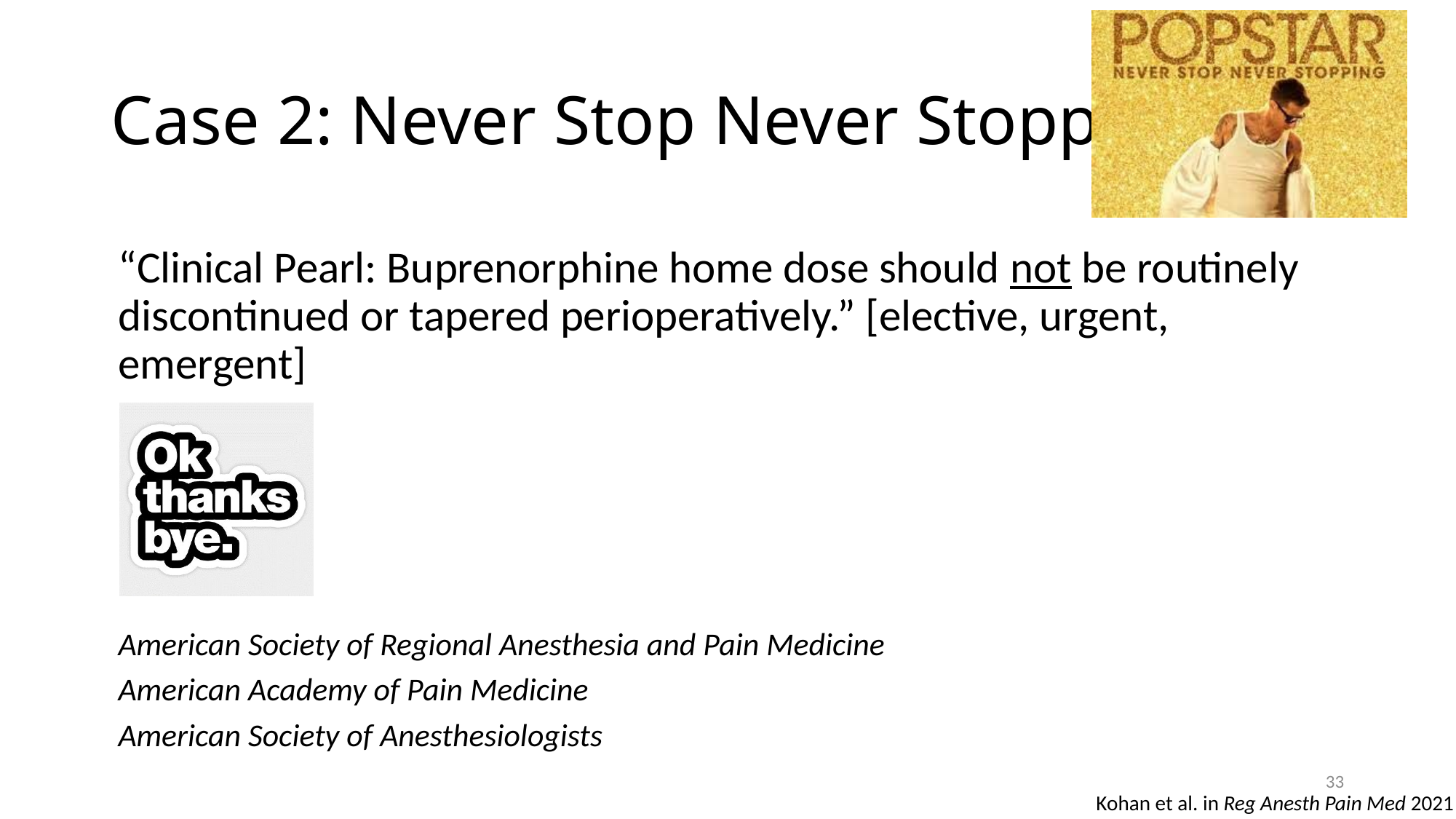

# Case 2: Never Stop Never Stopping
“Clinical Pearl: Buprenorphine home dose should not be routinely discontinued or tapered perioperatively.” [elective, urgent, emergent]
American Society of Regional Anesthesia and Pain Medicine
American Academy of Pain Medicine
American Society of Anesthesiologists
33
Kohan et al. in Reg Anesth Pain Med 2021

## Slide 34
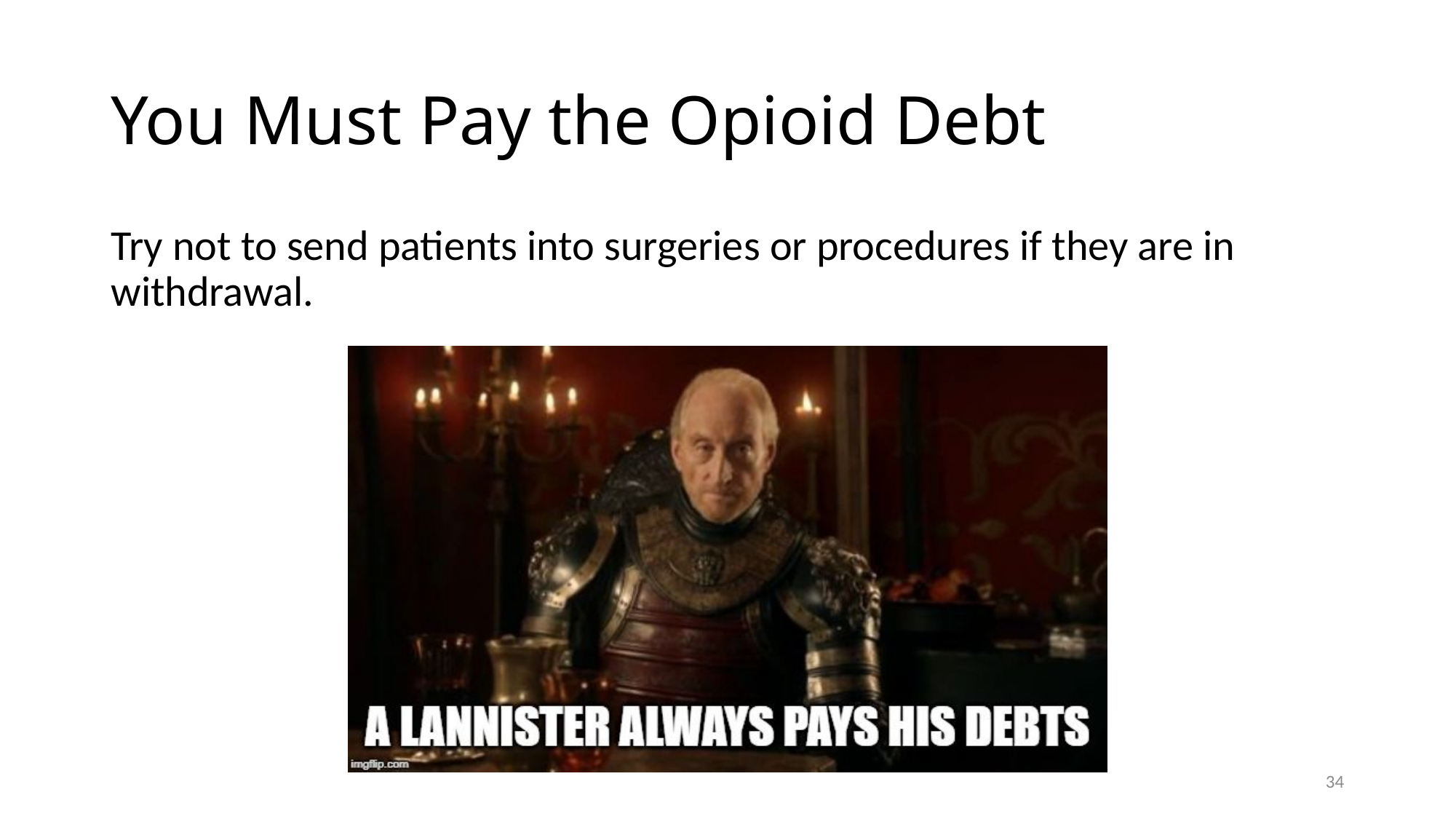

# You Must Pay the Opioid Debt
Try not to send patients into surgeries or procedures if they are in withdrawal.
34

## Slide 35
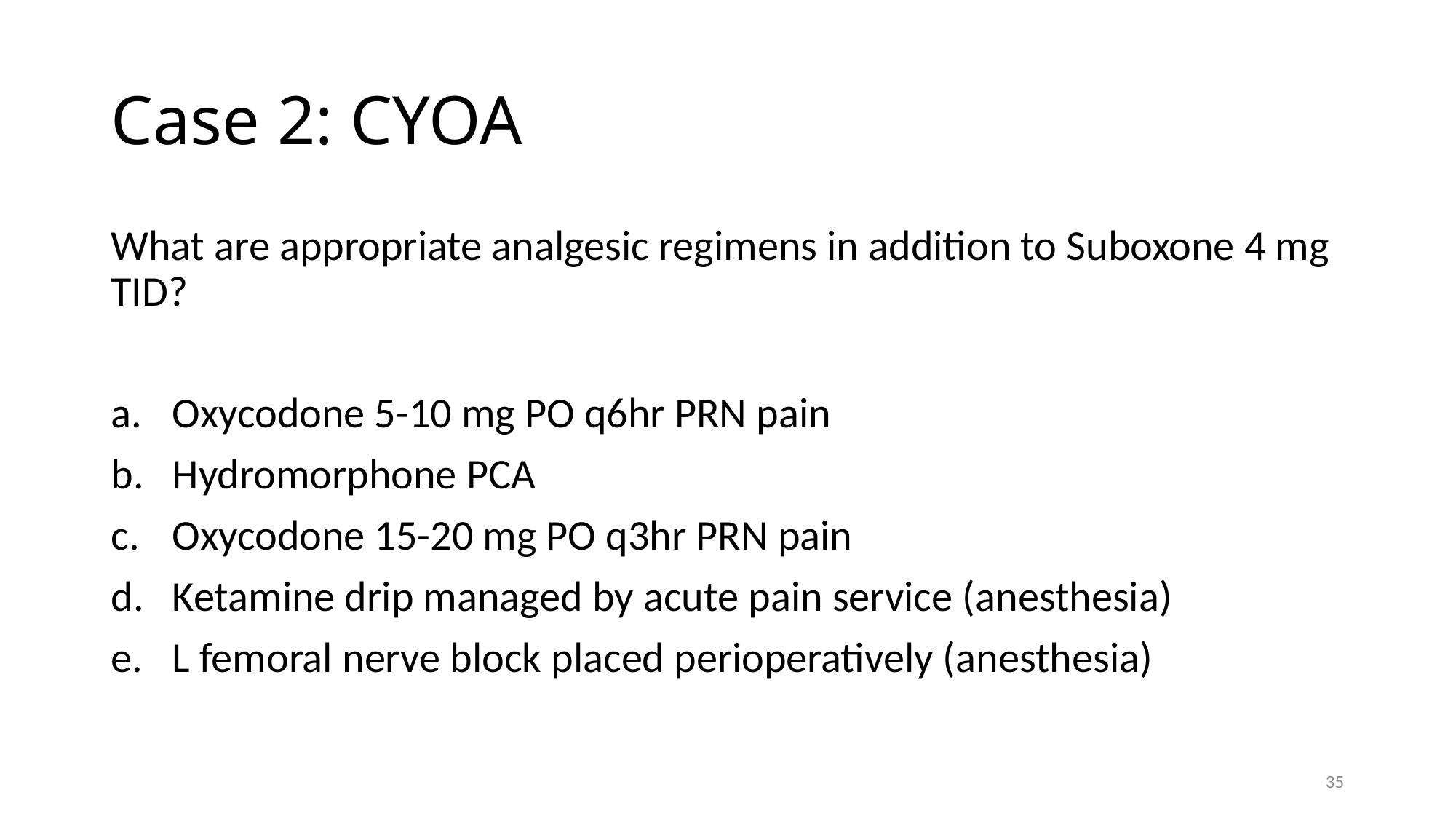

# Case 2: CYOA
What are appropriate analgesic regimens in addition to Suboxone 4 mg TID?
Oxycodone 5-10 mg PO q6hr PRN pain
Hydromorphone PCA
Oxycodone 15-20 mg PO q3hr PRN pain
Ketamine drip managed by acute pain service (anesthesia)
L femoral nerve block placed perioperatively (anesthesia)
35

## Slide 36
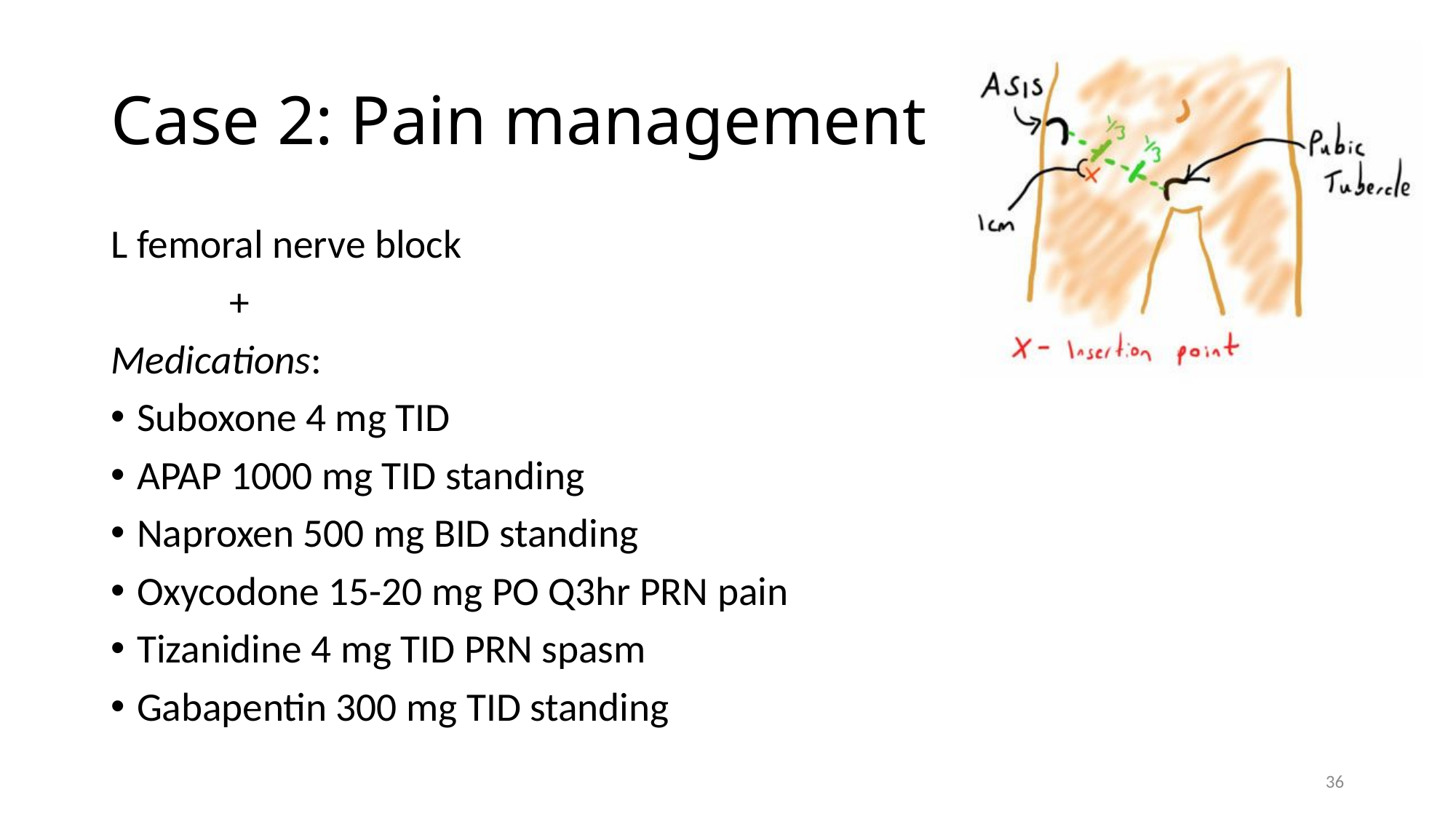

# Case 2: Pain management
L femoral nerve block
 +
Medications:
Suboxone 4 mg TID
APAP 1000 mg TID standing
Naproxen 500 mg BID standing
Oxycodone 15-20 mg PO Q3hr PRN pain
Tizanidine 4 mg TID PRN spasm
Gabapentin 300 mg TID standing
36

## Slide 37
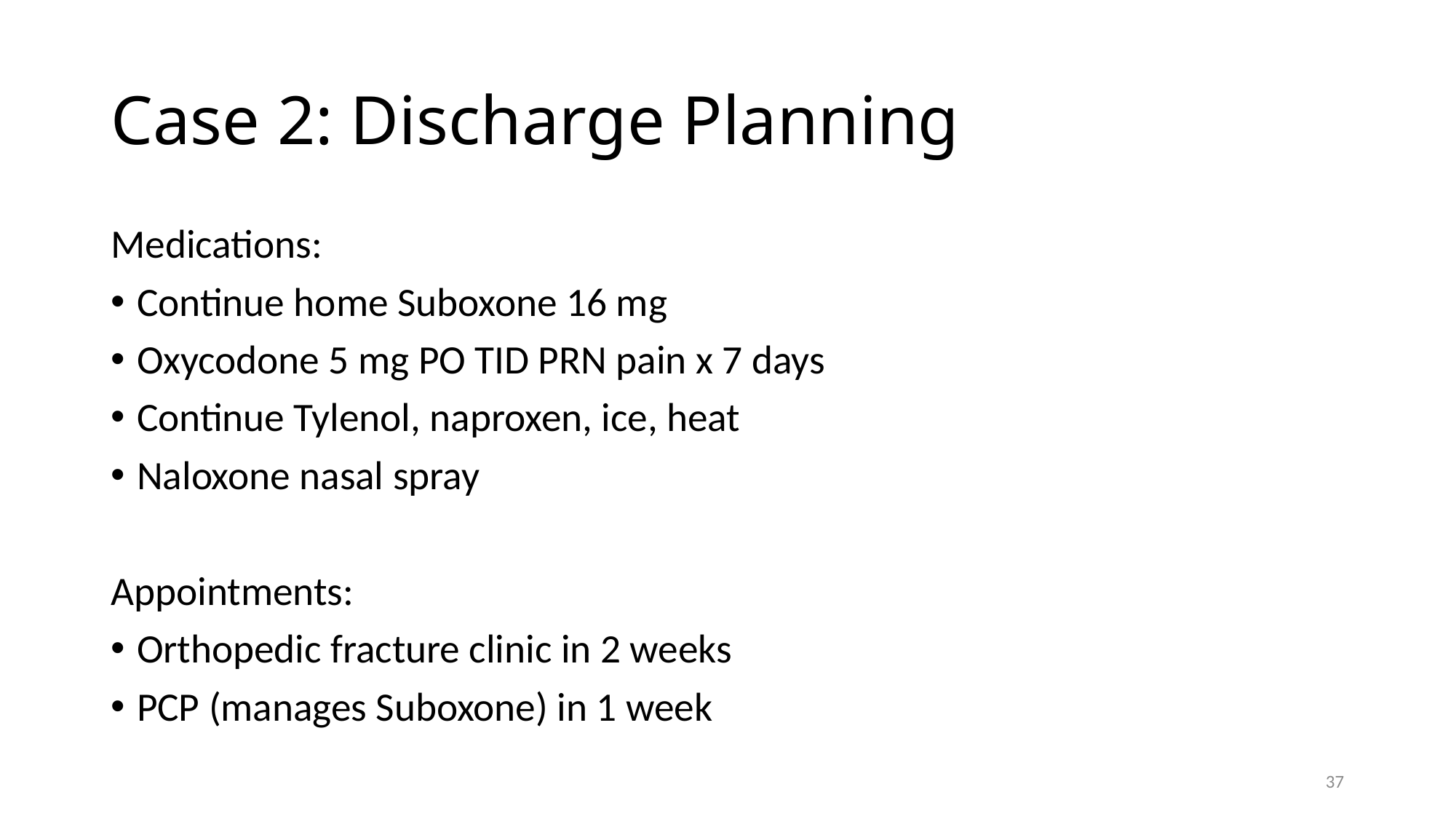

# Case 2: Discharge Planning
Medications:
Continue home Suboxone 16 mg
Oxycodone 5 mg PO TID PRN pain x 7 days
Continue Tylenol, naproxen, ice, heat
Naloxone nasal spray
Appointments:
Orthopedic fracture clinic in 2 weeks
PCP (manages Suboxone) in 1 week
37

## Slide 38
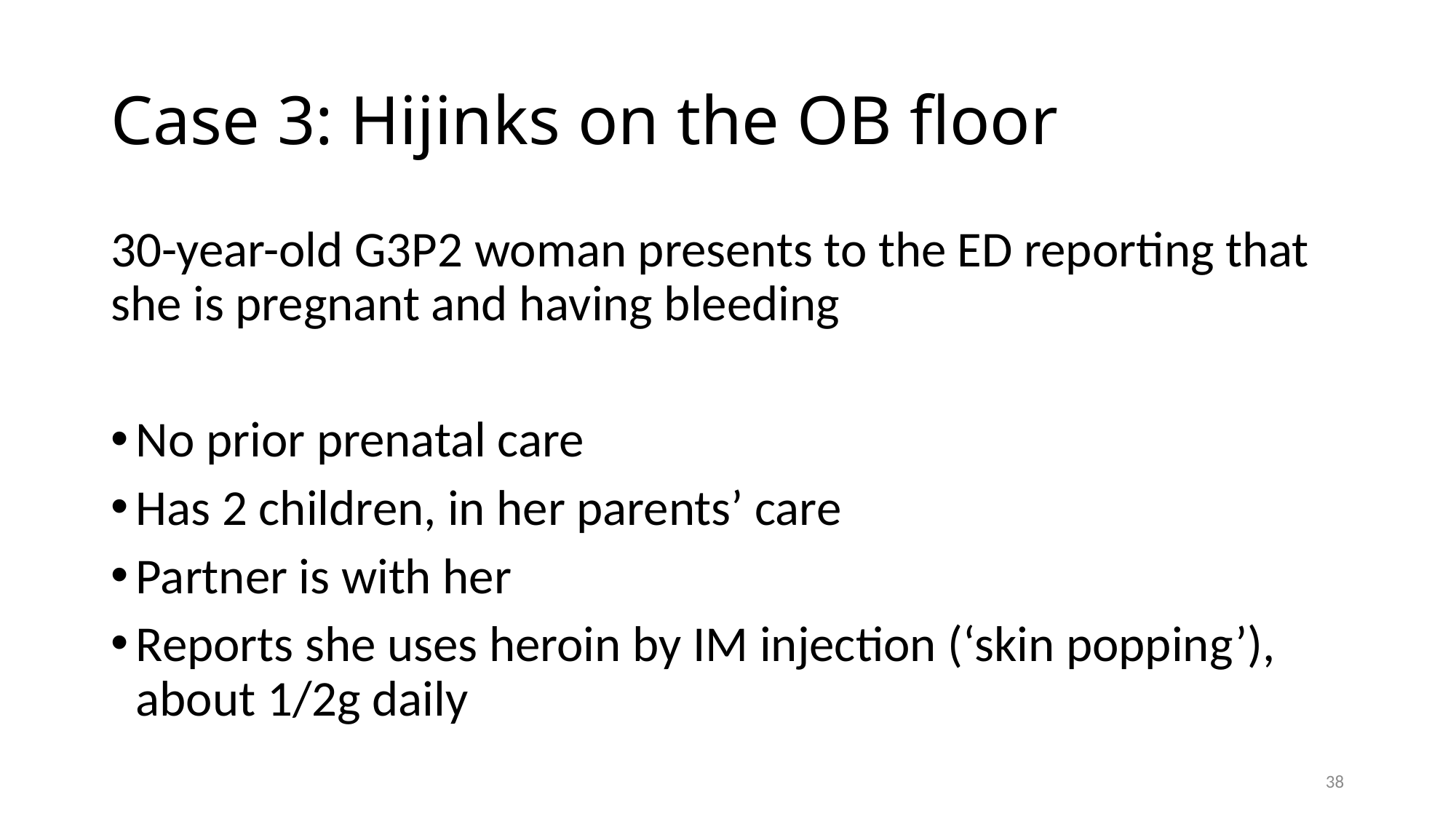

# Case 3: Hijinks on the OB floor
30-year-old G3P2 woman presents to the ED reporting that she is pregnant and having bleeding
No prior prenatal care
Has 2 children, in her parents’ care
Partner is with her
Reports she uses heroin by IM injection (‘skin popping’), about 1/2g daily
38

## Slide 39
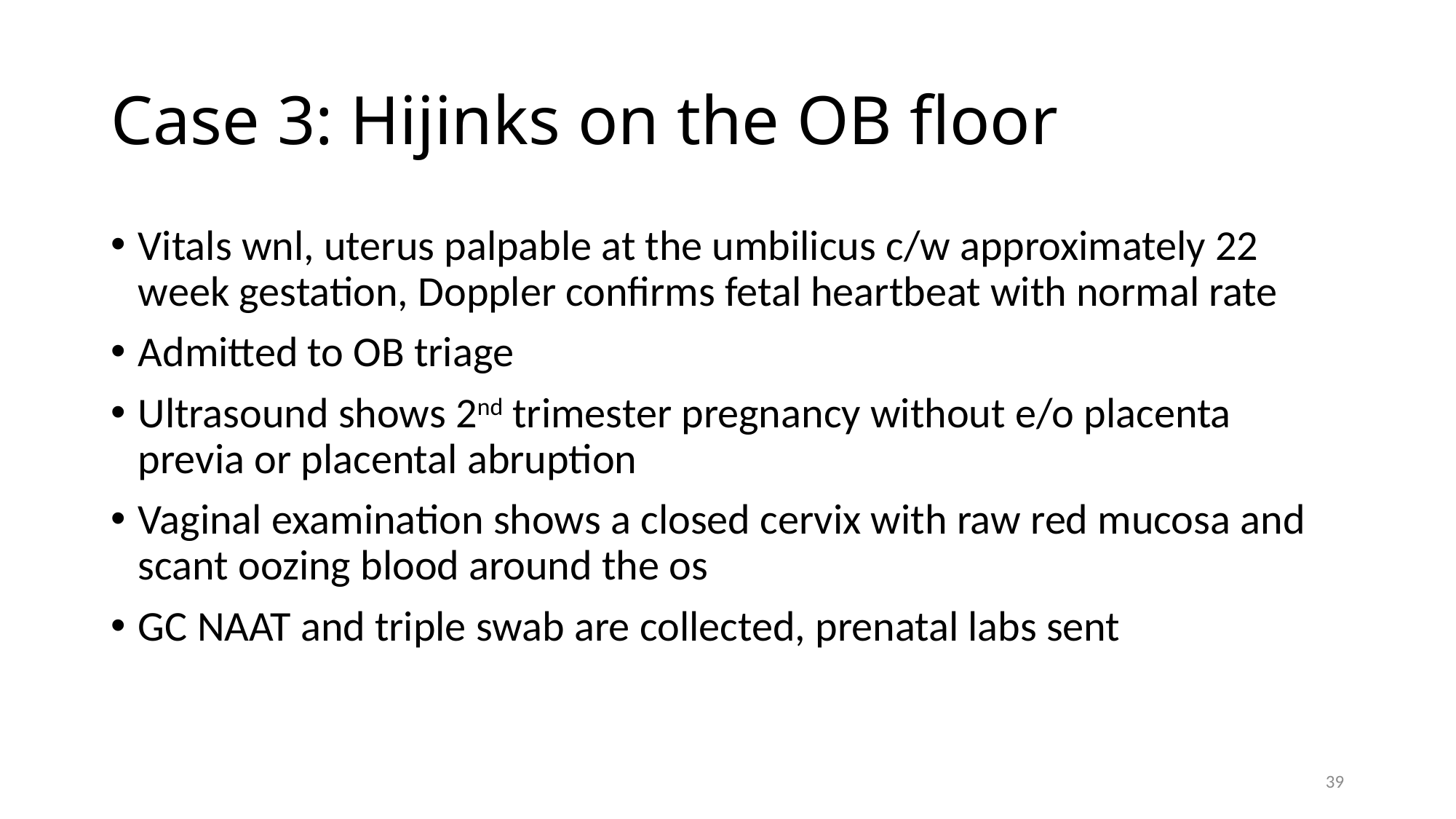

# Case 3: Hijinks on the OB floor
Vitals wnl, uterus palpable at the umbilicus c/w approximately 22 week gestation, Doppler confirms fetal heartbeat with normal rate
Admitted to OB triage
Ultrasound shows 2nd trimester pregnancy without e/o placenta previa or placental abruption
Vaginal examination shows a closed cervix with raw red mucosa and scant oozing blood around the os
GC NAAT and triple swab are collected, prenatal labs sent
39

## Slide 40
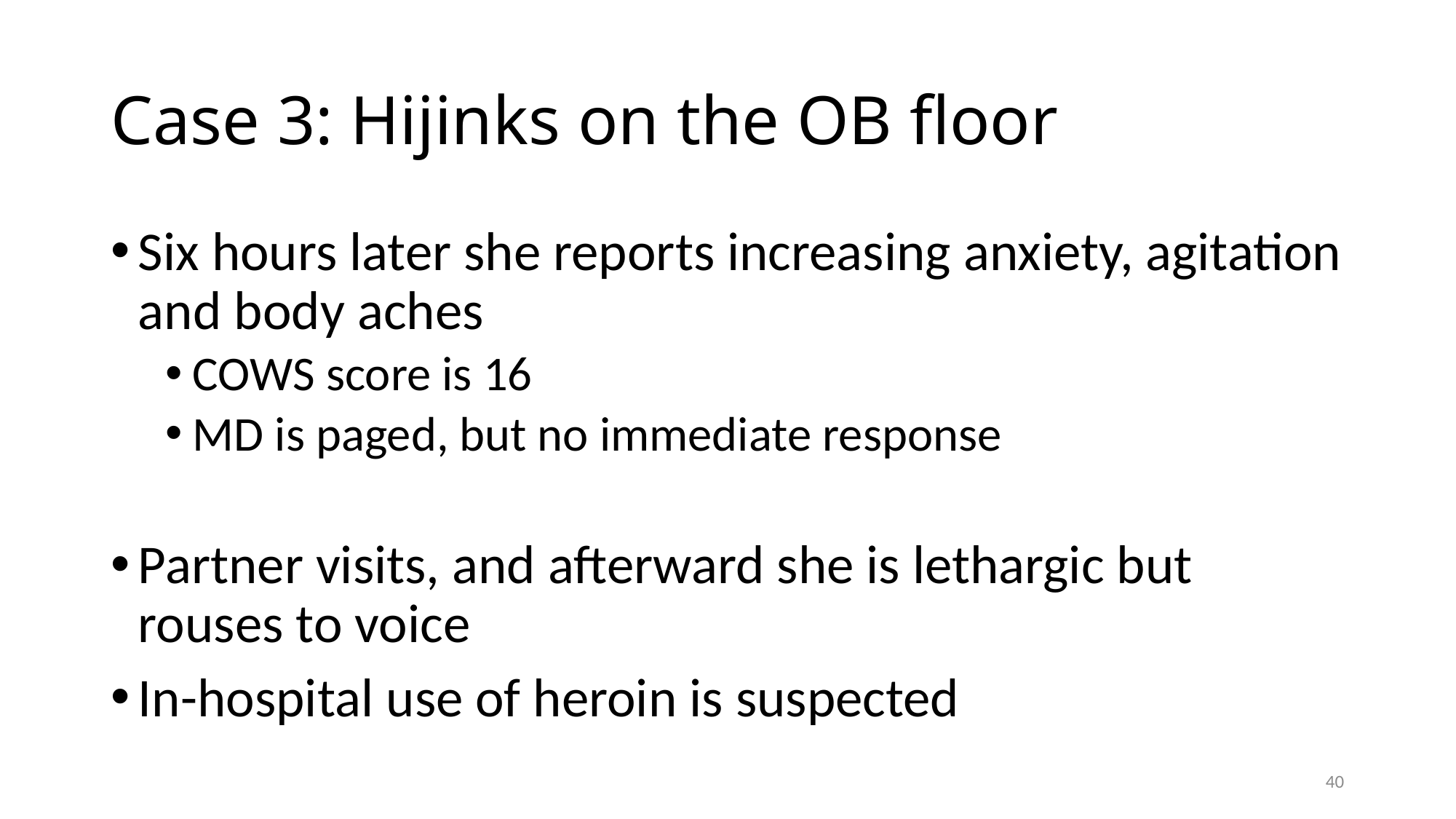

# Case 3: Hijinks on the OB floor
Six hours later she reports increasing anxiety, agitation and body aches
COWS score is 16
MD is paged, but no immediate response
Partner visits, and afterward she is lethargic but rouses to voice
In-hospital use of heroin is suspected
40

## Slide 41
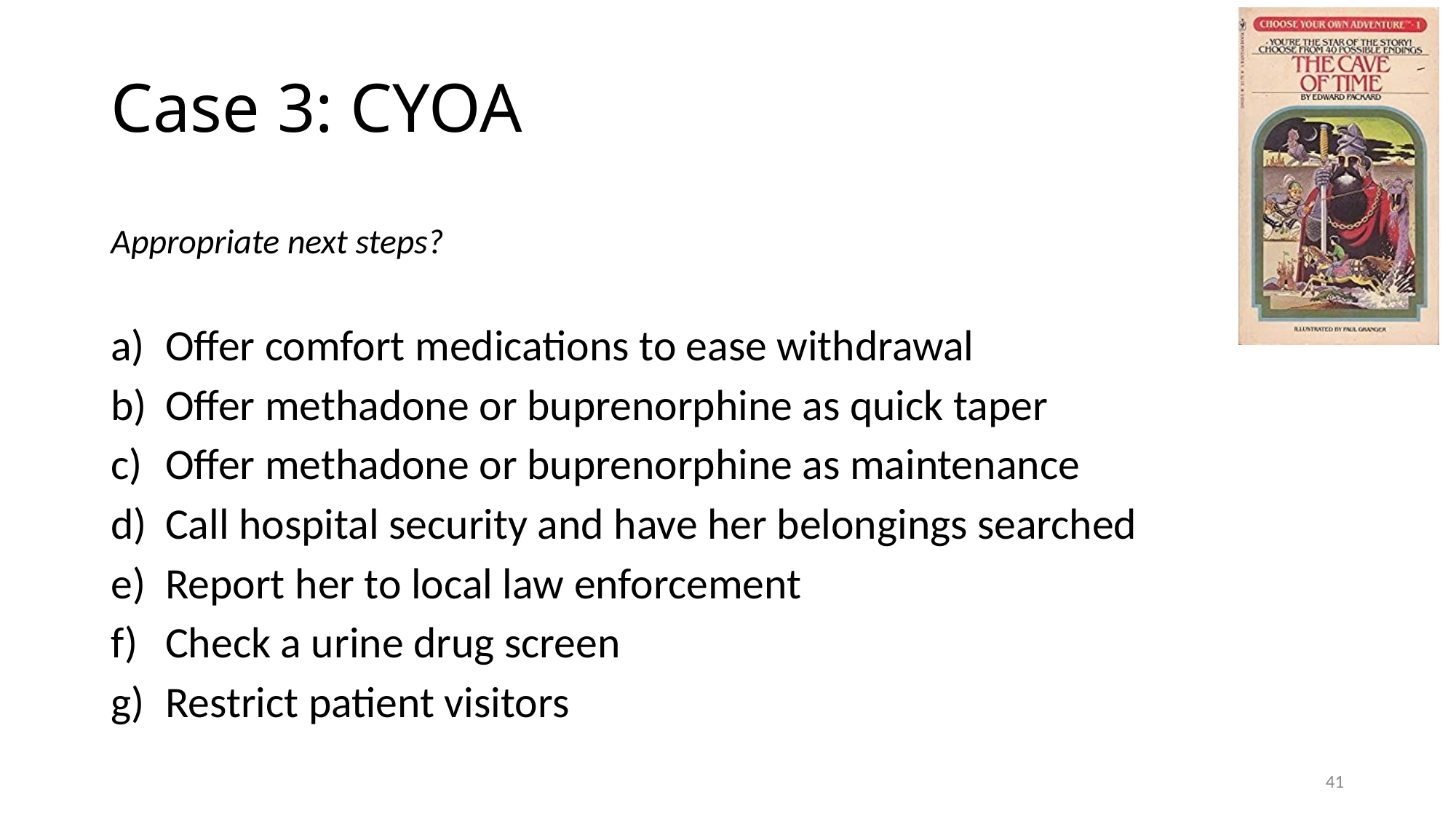

# Case 3: CYOA
Appropriate next steps?
Offer comfort medications to ease withdrawal
Offer methadone or buprenorphine as quick taper
Offer methadone or buprenorphine as maintenance
Call hospital security and have her belongings searched
Report her to local law enforcement
Check a urine drug screen
Restrict patient visitors
41

## Slide 42
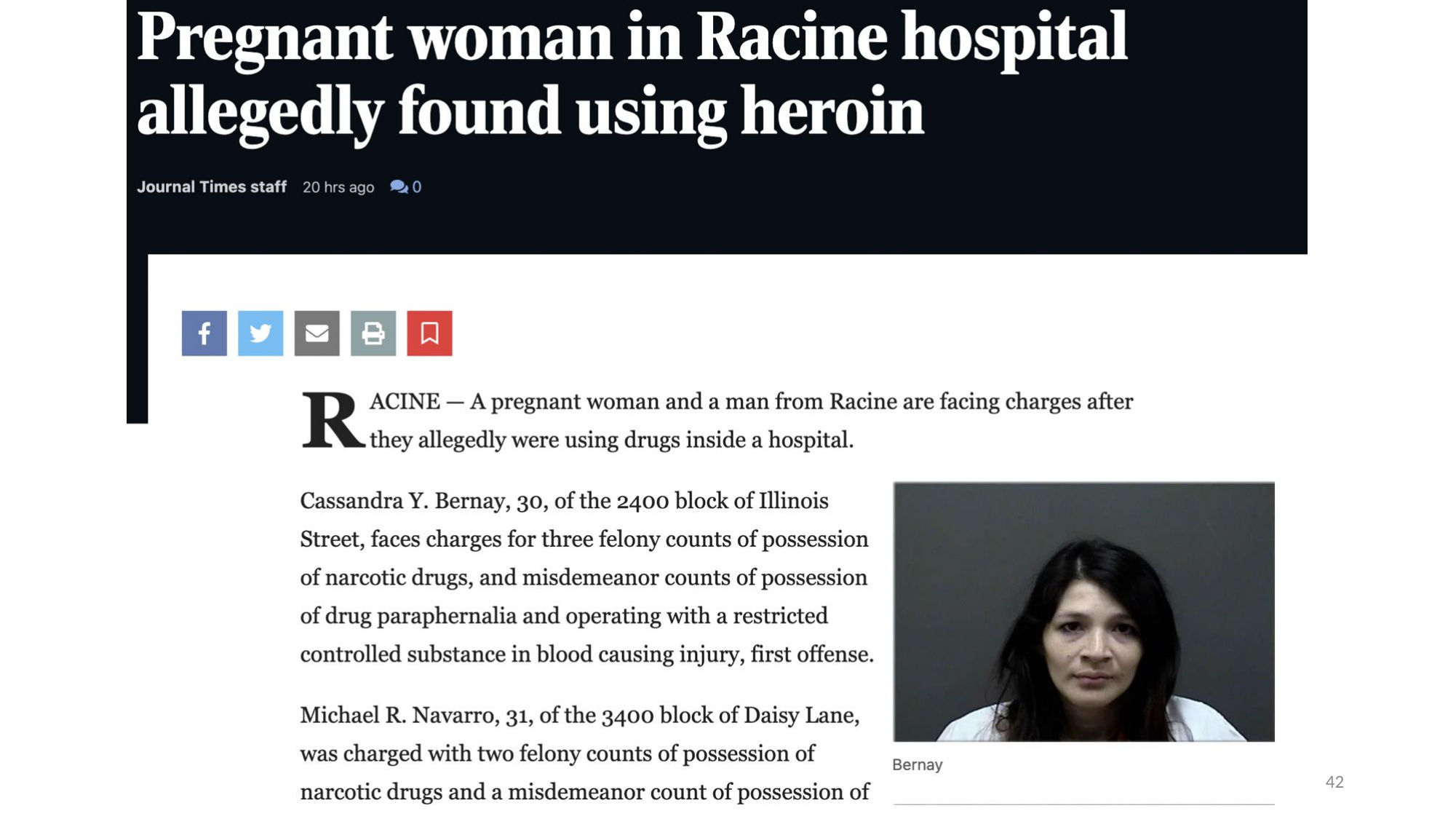

42

## Slide 43
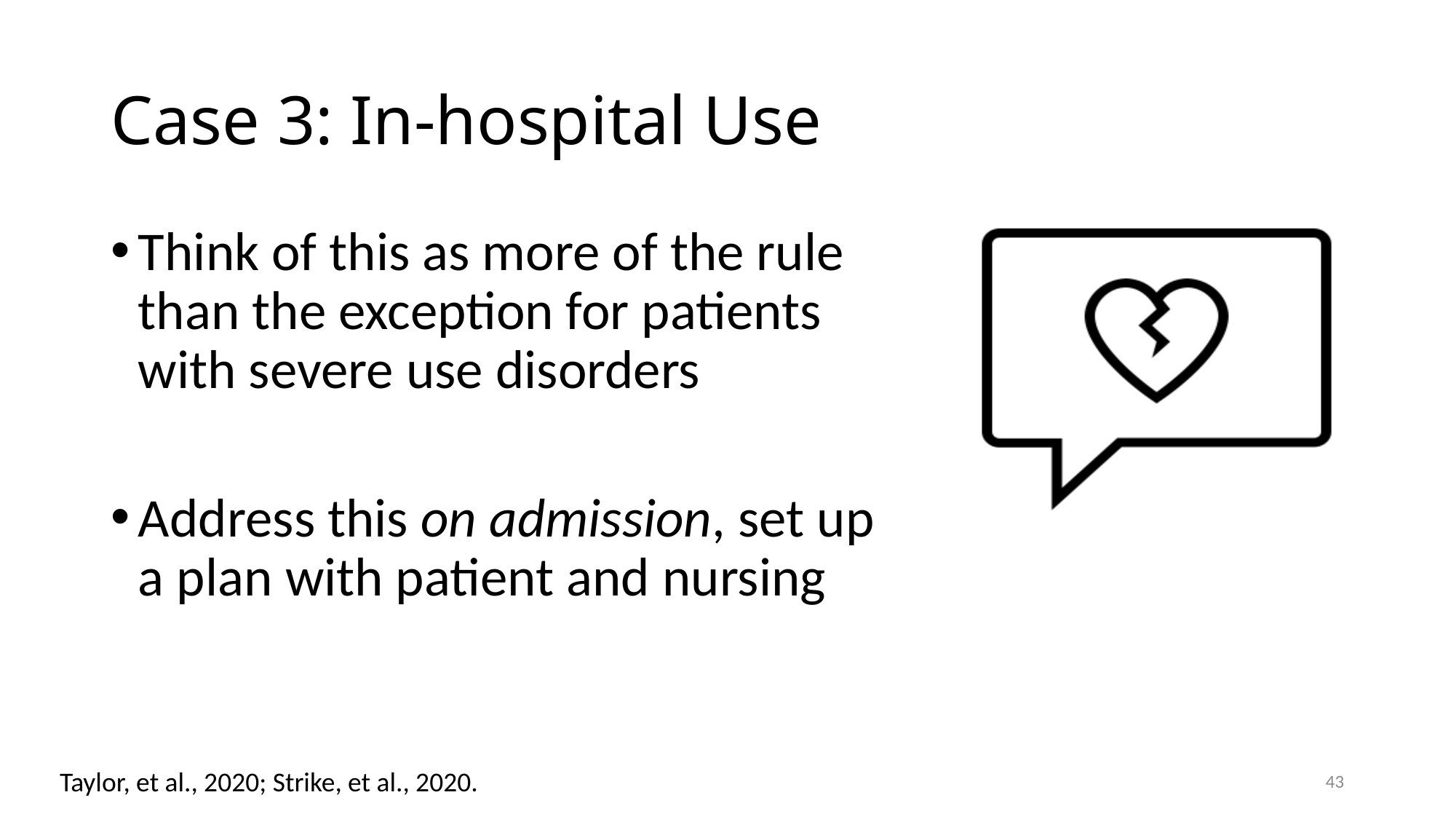

# Case 3: In-hospital Use
Think of this as more of the rule than the exception for patients with severe use disorders
Address this on admission, set up a plan with patient and nursing
Taylor, et al., 2020; Strike, et al., 2020.
43

## Slide 44
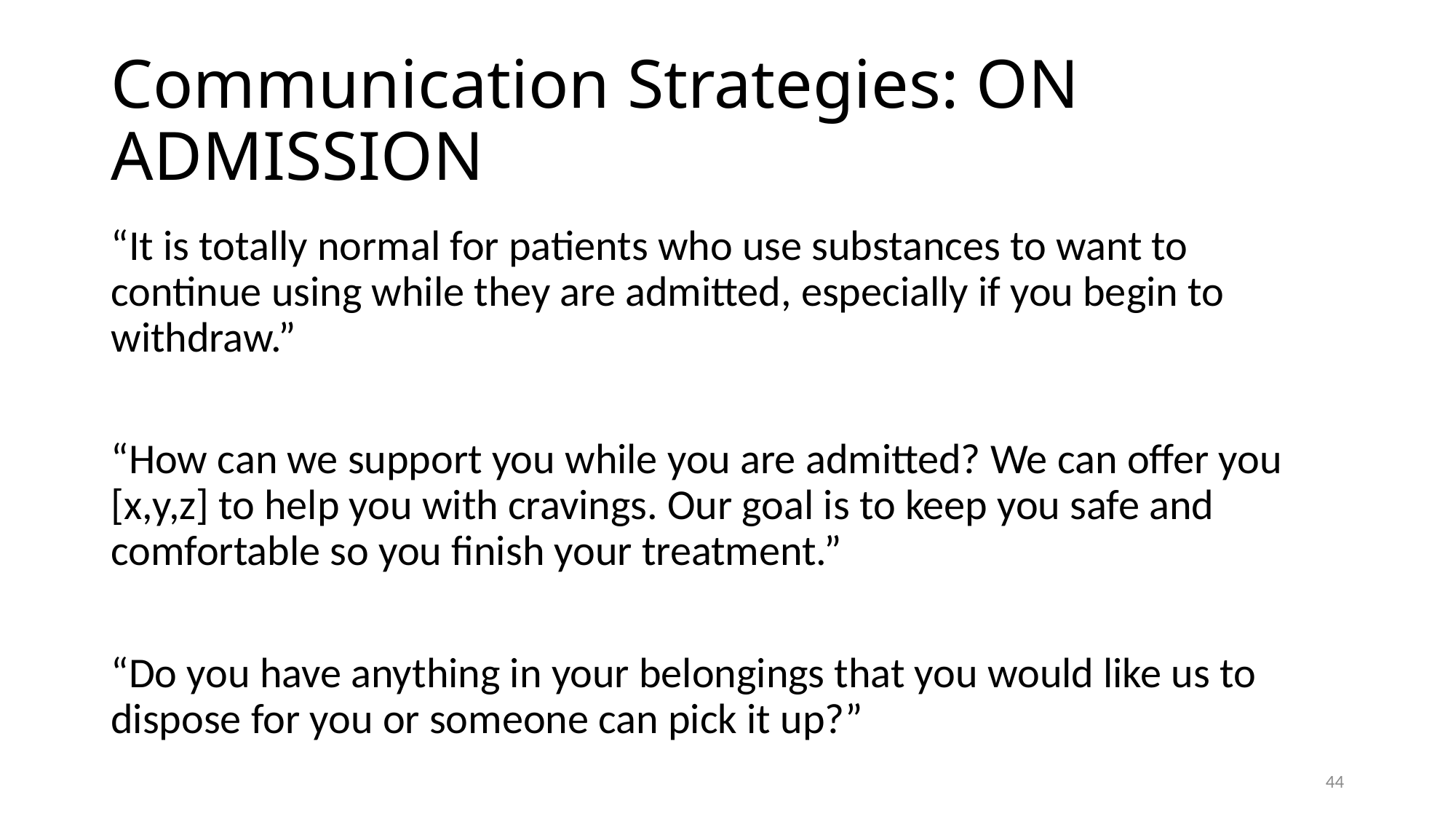

# Communication Strategies: ON ADMISSION
“It is totally normal for patients who use substances to want to continue using while they are admitted, especially if you begin to withdraw.”
“How can we support you while you are admitted? We can offer you [x,y,z] to help you with cravings. Our goal is to keep you safe and comfortable so you finish your treatment.”
“Do you have anything in your belongings that you would like us to dispose for you or someone can pick it up?”
44

## Slide 45
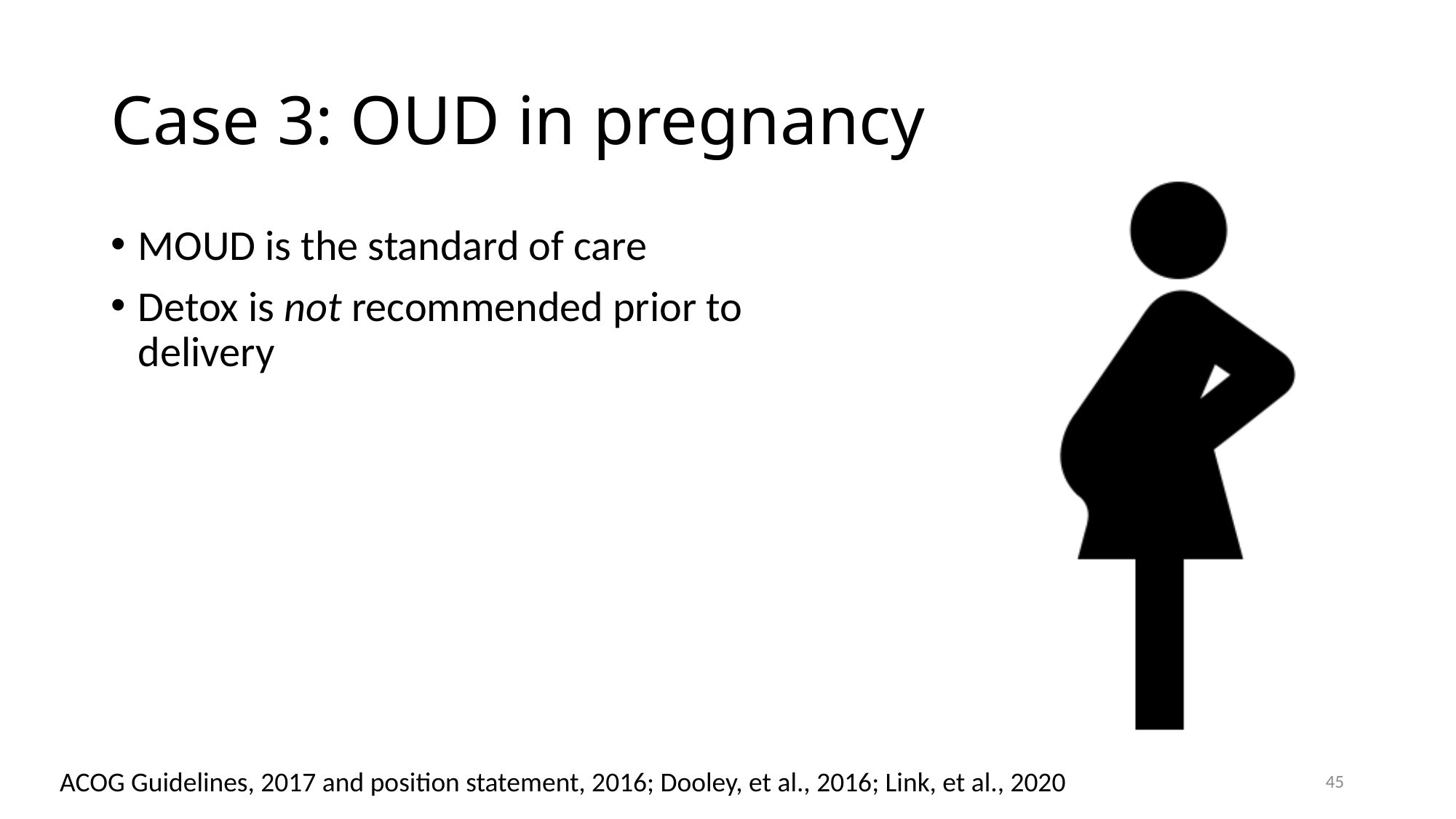

# Case 3: OUD in pregnancy
MOUD is the standard of care
Detox is not recommended prior to delivery
ACOG Guidelines, 2017 and position statement, 2016; Dooley, et al., 2016; Link, et al., 2020
45

## Slide 46
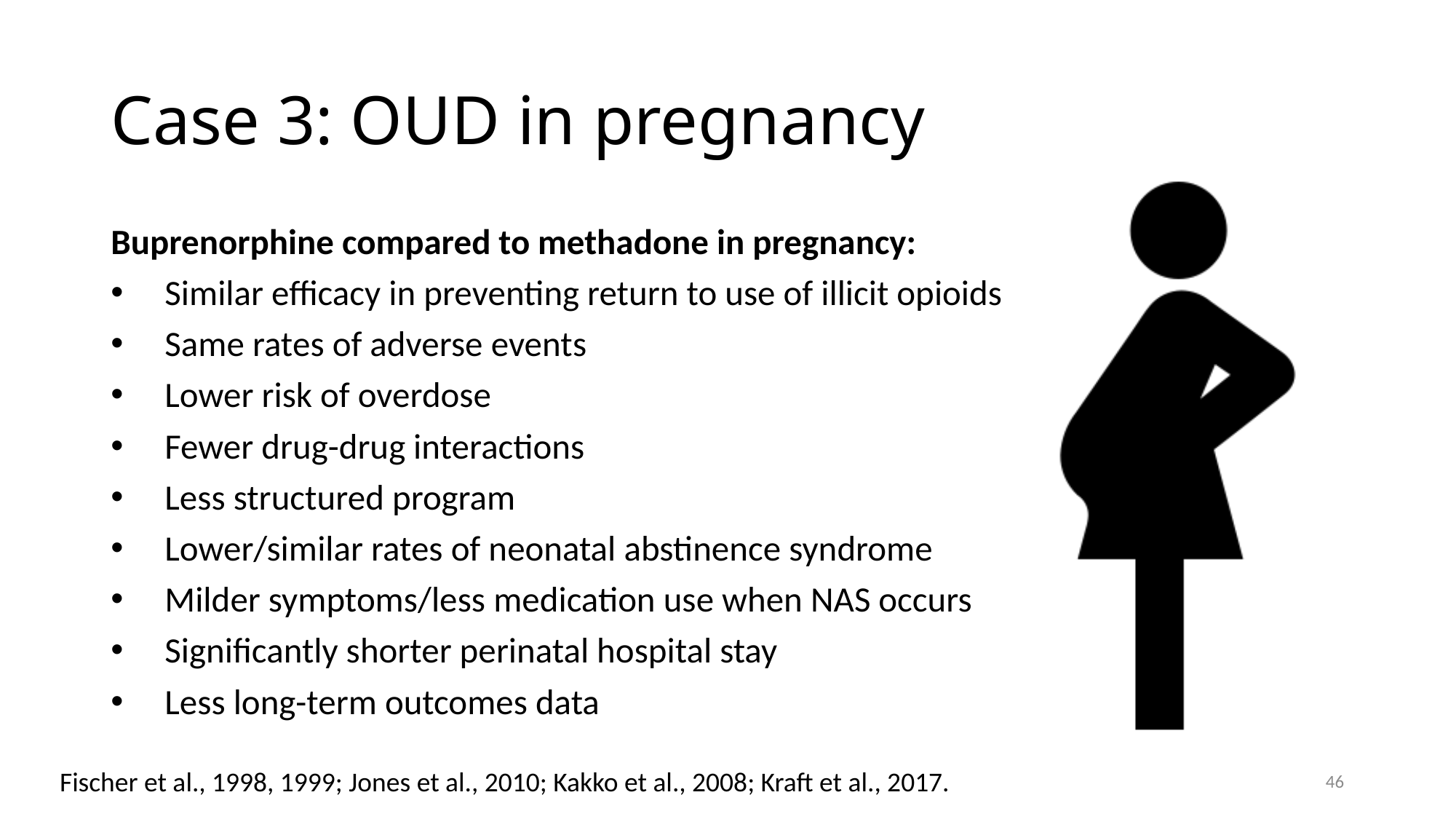

# Case 3: OUD in pregnancy
Buprenorphine compared to methadone in pregnancy:
Similar efficacy in preventing return to use of illicit opioids
Same rates of adverse events
Lower risk of overdose
Fewer drug-drug interactions
Less structured program
Lower/similar rates of neonatal abstinence syndrome
Milder symptoms/less medication use when NAS occurs
Significantly shorter perinatal hospital stay
Less long-term outcomes data
Fischer et al., 1998, 1999; Jones et al., 2010; Kakko et al., 2008; Kraft et al., 2017.
46

## Slide 47
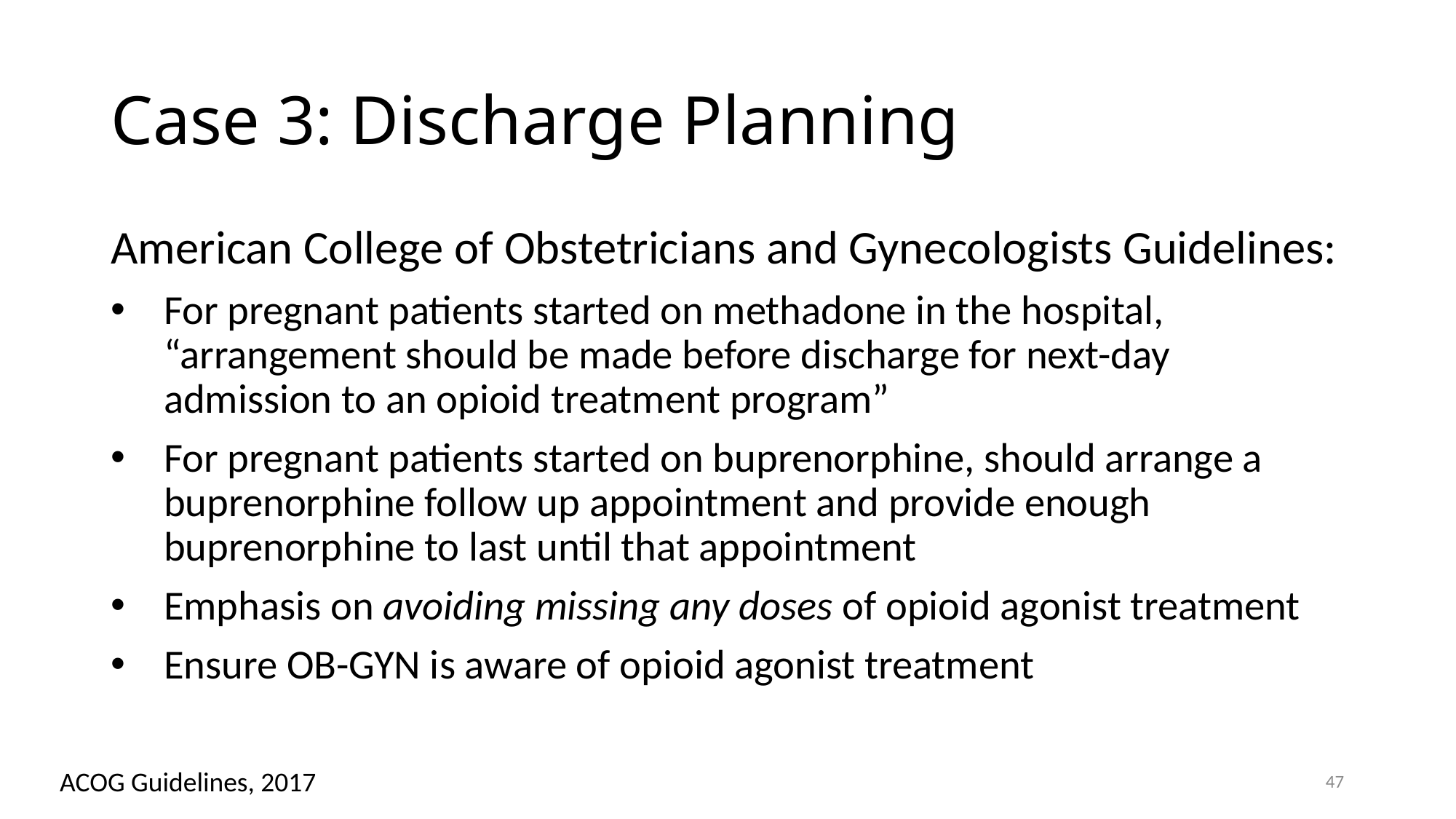

# Case 3: Discharge Planning
American College of Obstetricians and Gynecologists Guidelines:
For pregnant patients started on methadone in the hospital, “arrangement should be made before discharge for next-day admission to an opioid treatment program”
For pregnant patients started on buprenorphine, should arrange a buprenorphine follow up appointment and provide enough buprenorphine to last until that appointment
Emphasis on avoiding missing any doses of opioid agonist treatment
Ensure OB-GYN is aware of opioid agonist treatment
ACOG Guidelines, 2017
47

## Slide 48
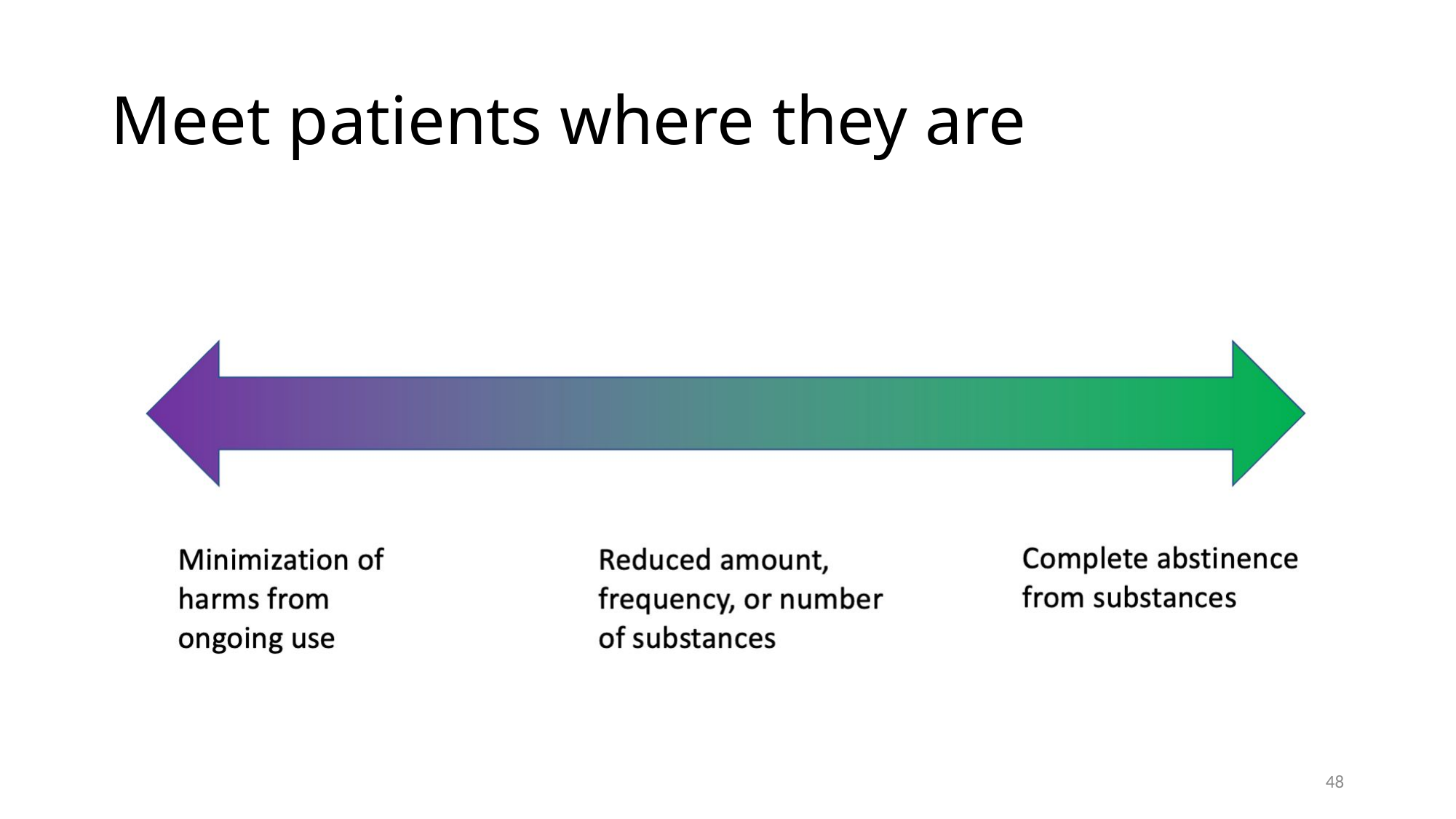

# Meet patients where they are
48

## Slide 49
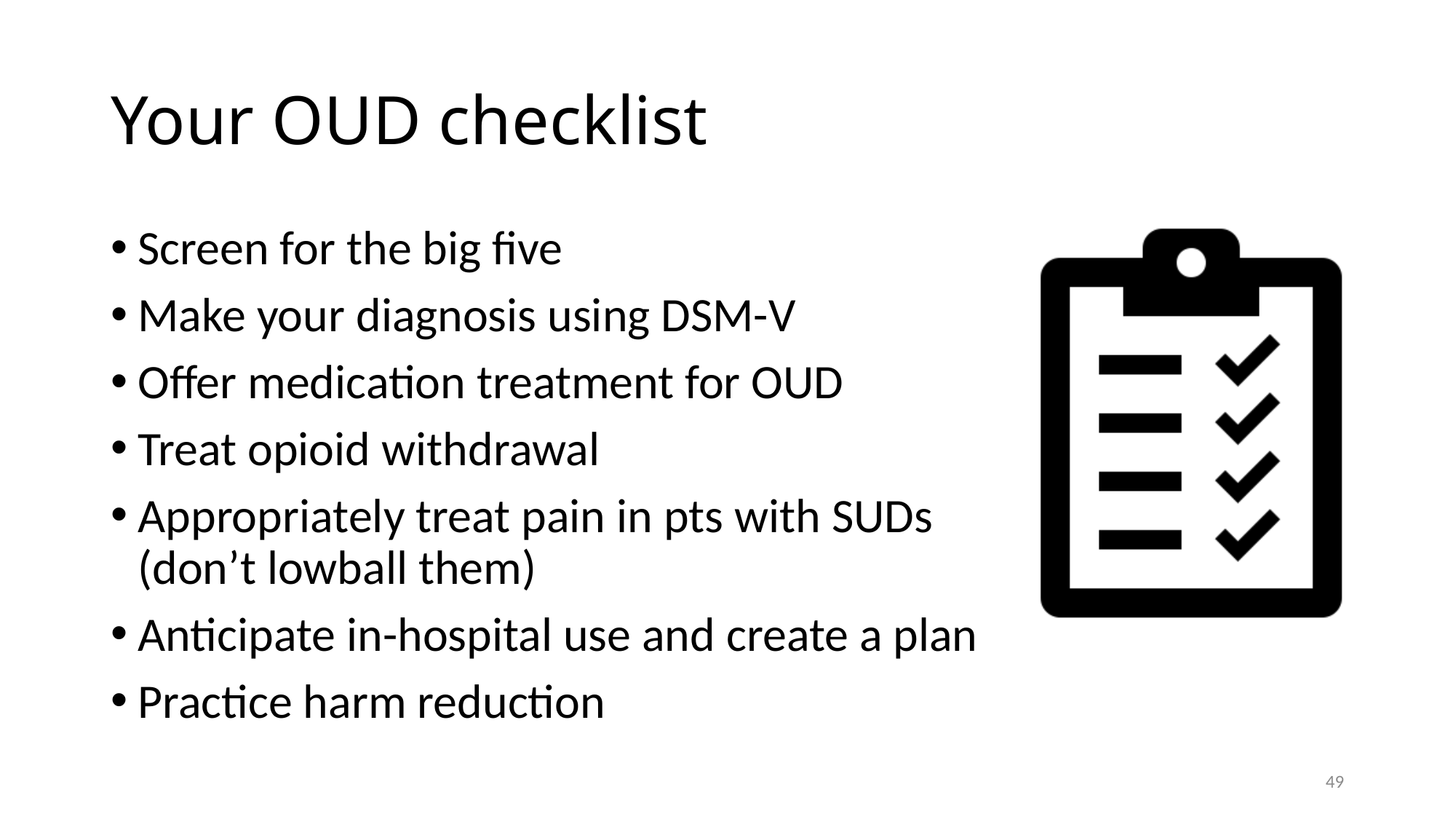

# Your OUD checklist
Screen for the big five
Make your diagnosis using DSM-V
Offer medication treatment for OUD
Treat opioid withdrawal
Appropriately treat pain in pts with SUDs (don’t lowball them)
Anticipate in-hospital use and create a plan
Practice harm reduction
49

## Slide 50
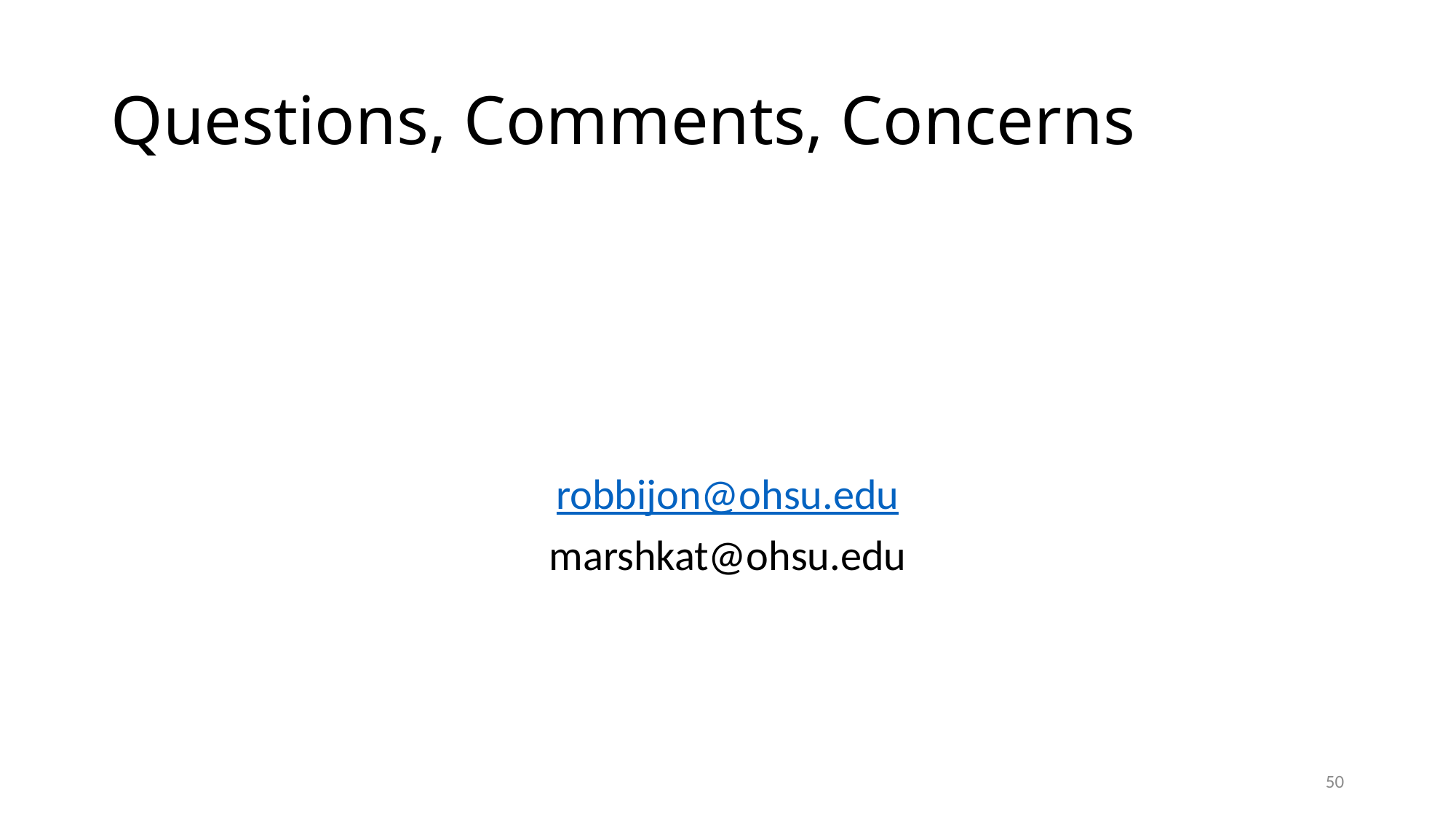

# Questions, Comments, Concerns
robbijon@ohsu.edu
marshkat@ohsu.edu
50
